# The multi-scale nature of the solar wind

**DOI:** 10.1007/s41116-019-0021-0

**Published:** 2019-12-09

**Authors:** Daniel Verscharen, Kristopher G. Klein, Bennett A. Maruca

**Affiliations:** 1 Mullard Space Science Laboratory, University College London, Dorking, RH5 6NT UK; 2 Space Science Center, University of New Hampshire, Durham, NH 03824 USA; 3 Lunar and Planetary Laboratory and Department of Planetary Sciences, University of Arizona, Tucson, AZ 85719 USA; 4 Bartol Research Institute, Department of Physics and Astronomy, University of Delaware, Newark, DE 19716 USA

**Keywords:** Solar wind, Spacecraft measurements, Coulomb collisions, Plasma waves and turbulence, Kinetic instabilities

## Abstract

The solar wind is a magnetized plasma and as such exhibits collective plasma behavior associated with its characteristic spatial and temporal scales. The characteristic length scales include the size of the heliosphere, the collisional mean free paths of all species, their inertial lengths, their gyration radii, and their Debye lengths. The characteristic timescales include the expansion time, the collision times, and the periods associated with gyration, waves, and oscillations. We review the past and present research into the multi-scale nature of the solar wind based on in-situ spacecraft measurements and plasma theory. We emphasize that couplings of processes across scales are important for the global dynamics and thermodynamics of the solar wind. We describe methods to measure in-situ properties of particles and fields. We then discuss the role of expansion effects, non-equilibrium distribution functions, collisions, waves, turbulence, and kinetic microinstabilities for the multi-scale plasma evolution.

**Electronic supplementary material:**

The online version of this article (10.1007/s41116-019-0021-0) contains supplementary material, which is available to authorized users.

## Introduction

The solar wind is a continuous magnetized plasma outflow that emanates from the solar corona. This extension of the Sun’s outer atmosphere propagates through interplanetary space. Its existence was first conjectured based on its interaction with planetary bodies in the solar system. Although the connection between solar activity and disturbances in the Earth’s magnetic field had been established in the nineteenth century (Sabine 1851, 1852; Hodgson 1859; Stewart 1861), the connection of these events with “corpuscular radiation” was not made until the early twentieth century (Birkeland 1914; Chapman 1917). The arguably first appearance of the notion of a continuous “swarm of ions proceeding from the Sun” in the literature dates back to a footnote by Eddington (1910) as an explanation for the observed shape of cometary tails. Later, Hoffmeister (1943) summarized multiple comet observations and suggested that some form of solar corpuscular radiation is responsible for the observed lag of comet ion tails with respect to the heliocentric radius vector (for the link between solar activity and comet tails, see also Ahnert 1943). Biermann (1951) revisited the relation between comet tails and solar corpuscular radiation by quantifying the momentum transfer from the solar wind to cometary ions. He especially noted that the solar radiation pressure is insufficient to explain the observed structures (Milne 1926) and that the corpuscular radiation is more variable than the electromagnetic radiation emitted by the Sun. The origin of the solar corpuscular radiation, however, remained unclear until Parker (1958) showed that a hot solar corona cannot maintain a hydrostatic equilibrium. Instead, the pressure-gradient force overcomes gravity and leads to a radial acceleration of the coronal plasma to supersonic velocities, which Parker called “solar wind” in contrast to a subsonic “solar breeze” (Chamberlain 1961), which was later found to be unstable (Velli 1994). Soon after this prediction, the solar wind was measured in situ by spacecraft (Gringauz et al. 1960; Neugebauer and Snyder 1962). For the last four decades, the solar wind has been monitored almost continuously in situ. Parker’s underlying concept is the mainstream paradigm for the acceleration of the solar wind, but many questions remain unresolved. For example, we still have not identified the mechanisms that heat the solar corona to temperatures orders of magnitude higher than the photospheric temperature, albeit this discovery was made some 80 years ago (Grotrian 1939; Edlén 1943). As we discuss the observed features of the solar wind in this review, we will encounter further deficiencies in our understanding that require more detailed analyses beyond Parker’s model. In this process, we will find many observational facts that models of coronal heating and solar-wind acceleration must explain in order to achieve a realistic and consistent description of the physics of the solar wind.

In the first section of this review, we lay out the various characteristic length and timescales in the solar wind and motivate our thesis that this multi-scale nature defines the evolution of the solar wind. We then introduce the observed large-scale, global features and the microphysical, kinetic features of the solar wind as well as the mathematical basis to describe the related processes.

**Table 1:** The multiple characteristic plasma parameters (top), length scales (middle), and timescales (bottom) in the solar wind

Symbol	Solar wind	(Upper) Corona	Definition
$n_{\mathrm {p}}$, $n_{\mathrm {e}}$	$3\,\mathrm {cm}^{-3}$	$10^6\,\mathrm {cm}^{-3}$	Proton and electron number density
$T_{\mathrm {p}}$, $T_{\mathrm {e}}$	$10^5\,\mathrm {K}$	$10^6\,\mathrm {K}$	Proton and electron temperature
*B*	$3\times 10^{-5}\,\mathrm {G}$	1 G	Magnetic field strength
$\lambda _{\mathrm {mfp,p}}$	3 au	100 Mm	Proton collisional mean free path
*L*	1 au	100 Mm	Characteristic size of the system
$d_{\mathrm {p}}$	140 km	230 m	Proton inertial length
$\rho _{\mathrm {p}}$	160 km	13 m	Proton gyration radius
$d_{\mathrm {e}}$	3 km	5 m	Electron inertial length
$\rho _{\mathrm {e}}$	2 km	30 cm	Electron gyration radius
$\lambda _{\mathrm {p}}$, $\lambda _{\mathrm {e}}$	12 m	7 cm	Proton and electron Debye lengths
$\varPi _{\nu _{\mathrm {c}}}$	120 d	2 h	Proton collision time
$\tau$	2.4 d	10 min	Expansion time
$\varPi _{\varOmega _{\mathrm {p}}}$	26 s	$660\,\mu \mathrm {s}$	Proton gyration period
$\varPi _{\omega _{\mathrm {pp}}}$	$3\,\mathrm {ms}$	$5\,\mu \mathrm {s}$	Proton plasma period
$\varPi _{\varOmega _{\mathrm {e}}}$	14 ms	360 ns	Electron gyration period
$\varPi _{\omega _{\mathrm {pe}}}$	$70\,\mu \mathrm {s}$	110 ns	Electron plasma period

This table shows typical parameters in the solar wind at 1 au and in the upper solar corona ($\sim$ 100 Mm above photosphere). For each angular frequency $\omega$, the associated timescale is given by $\varPi _{\omega }\equiv 2\pi /|\omega |$

**Fig. 1:**
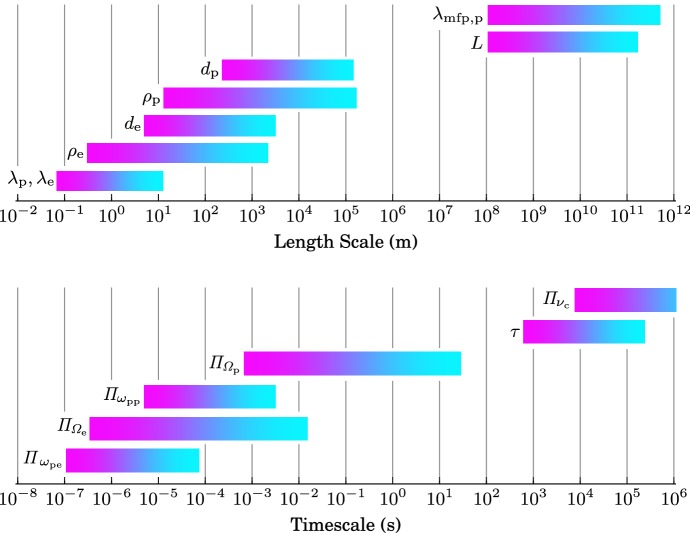
Graphical representation of the characteristic length scales (top) and timescales (bottom) in the solar wind. The bar lengths represent the typical range for each scale given in Table 1. The magenta end of each bar indicates the typical coronal value, and the cyan end of each bar indicates the typical value at 1 au

### The characteristic scales in the solar wind

Table 1 lists typical values for the characteristic plasma parameters and scales in the solar wind at 1 au and in the upper solar corona that we introduce and define in this section. It is important to remember that all of these quantities vary widely in time and may differ significantly between thermal and superthermal particle populations. We illustrate the broad range of the characteristic length scales and timescales in Fig. 1.

The solar wind expands to a heliocentric distance of about 90 au, where it transitions to a subsonic flow by crossing the solar-wind termination shock (Stone et al. 2005; Burlaga et al. 2008). Although we do not expound upon the physics of the outer heliosphere and the interaction of the solar wind with the interstellar medium, this is the largest spatial scale in the supersonic solar wind. Considering the inner heliosphere (i.e., the spherical volume centered around the Sun within Earth’s orbit), we identify the characteristic *size of the system* as $L\sim 1\,\mathrm {au}$. For a typical radial solar-wind flow speed $U_r$ in the range of 300 km/s to 800 km/s (Lopez and Freeman 1986), we find an *expansion time* of

1 $$\begin{aligned} \tau \sim \frac{L}{U_r}\sim 2.4\,\mathrm {d} \end{aligned}$$

for the solar wind from the Sun to 1 au. The Sun’s siderial rotation period at its equator,

2 $$\begin{aligned} \tau _{\mathrm {rot}}\sim 25\,\mathrm {d}, \end{aligned}$$

introduces another characteristic global timescale.

In addition to the outer size of the system, a plasma has multiple characteristic scales due to the interactions of its free charges with electric and magnetic fields. In a homogeneous and constant magnetic field $\mathbf{B}_0$, a plasma particle with charge $q_j$ and mass $m_j$ (where *j* denotes the particle species) experiences a continuous deflection of its trajectory due to the Lorentz force. The frequency associated with this helical motion is given by the *gyro-frequency*^1^ (also called the *cyclotron frequency*)

3 $$\begin{aligned} \varOmega _j\equiv \frac{q_jB_0}{m_jc}, \end{aligned}$$

where *c* is the speed of light in vacuum. The timescale for one closed loop around the magnetic field is then given by the *gyro-period* $\varPi _{\varOmega _j}\equiv 2\pi /|\varOmega _j|$. In the solar wind at 1 au, $\varPi _{\varOmega _{\mathrm {p}}}\sim 26\,\mathrm {s}$ and $\varPi _{\varOmega _{\mathrm {e}}}\sim 14\,\mathrm {ms}$, where the index $\mathrm {p}$ represents protons and the index $\mathrm {e}$ represents electrons. On the other hand, in the upper corona (about 100 Mm above the photosphere), where the magnetic field is much stronger than in the solar wind, $\varPi _{\varOmega _{\mathrm {p}}}\sim 660\,\mu \mathrm {s}$ and $\varPi _{\varOmega _{\mathrm {e}}}\sim 360\,\mathrm {ns}$. Aside from protons, $\alpha$-particles (i.e., fully ionized helium atoms) are also dynamically important in the solar wind since they account for $\lesssim \,20\%$ of the mass density.

We define the perpendicular thermal speed as

4 $$\begin{aligned} w_{\perp j}\equiv \sqrt{\frac{2k_{\mathrm {B}}T_{\perp j}}{m_j}} \end{aligned}$$

and the parallel thermal speed as

5 $$\begin{aligned} w_{\parallel j}\equiv \sqrt{\frac{2k_{\mathrm {B}}T_{\parallel j}}{m_j}}, \end{aligned}$$

where $T_{\perp j}$ ($T_{\parallel j}$) is the temperature of particle species *j* in the direction perpendicular (parallel) to $\mathbf{B}_0$ and $k_{\mathrm {B}}$ is the Boltzmann constant. We define the concept of temperatures perpendicular and parallel to $\mathbf{B}_0$ in Eqs. (38) and (39). Assuming a thermal distribution of particles with a perpendicular thermal speed $w_{\perp j}$, the characteristic size of the gyration orbit is given by the *gyro-radius*

6 $$\begin{aligned} \rho _j\equiv \frac{w_{\perp j}}{\left| \varOmega _j\right| }. \end{aligned}$$

At 1 au, solar-wind gyro-radii are typically $\rho _{\mathrm {p}}\sim 160\,\mathrm {km}$ and $\rho _{\mathrm {e}}\sim 2\,\mathrm {km}$. In the upper corona, the gyro-radii are smaller: $\rho _{\mathrm {p}}\sim 13\,\mathrm {m}$ and $\rho _{\mathrm {e}}\sim 30\,\mathrm {cm}$.

The *plasma frequency*

7 $$\begin{aligned} \omega _{\mathrm {p}j}\equiv \sqrt{\frac{4\pi n_{0j}q_j^2}{m_j}}, \end{aligned}$$

where $n_{0j}$ is the background number density of species *j*, corresponds to the characteristic timescale for electrostatic interactions in the plasma: $\varPi _{\omega _{\mathrm {p}j}}\equiv 2\pi /\omega _{\mathrm {p}j}$. In the solar wind at 1 au, $\varPi _{\omega _{\mathrm {pp}}}\sim 3\,\mathrm {ms}$ and $\varPi _{\omega _{\mathrm {pe}}}\sim 70\,\mu \mathrm {s}$. These timescales are even shorter in the corona: $\varPi _{\omega _{\mathrm {pp}}}\sim 5\,\mu \mathrm {s}$ and $\varPi _{\omega _{\mathrm {pe}}}\sim 110\,\mathrm {ns}$. A reduction of the local electron number density (e.g., through a spatial displacement of a number of electrons with respect to the ions) leads to an oscillation of the electrons with respect to the ions, in which the electrostatic force due to the displaced charge serves as the restoring force. This *plasma oscillation* occurs with a frequency $\sim \omega _{\mathrm {pe}}$. In addition, light waves cannot propagate at frequencies $\lesssim \,\omega _{\mathrm {pe}}$ in a plasma as the free plasma charges shield the wave’s electromagnetic fields so that the wave amplitude drops off exponentially with distance when the wave frequency is $\lesssim \,\omega _{\mathrm {pe}}$. The exponential decay length associated with this shielding is given by the skin-depth $d_{\mathrm {e}}\equiv c/\omega _{\mathrm {pe}}$.

More generally, we define the *skin-depth* (also called the *inertial length*) of species *j* as

8 $$\begin{aligned} d_j\equiv \frac{c}{\omega _{\mathrm {p}j}}=\frac{v_{{\mathrm {A}}j}}{|\varOmega _j|}, \end{aligned}$$

where

9 $$\begin{aligned} v_{{\mathrm {A}}j}\equiv \frac{B_{0}}{\sqrt{4\pi n_{0j} m_j}} \end{aligned}$$

is the *Alfvén speed* of species *j*. In the solar wind at 1 au, $d_{\mathrm {p}}\sim 140 \,\mathrm {km}$ and $d_{\mathrm {e}}\sim 3\,\mathrm {km}$. In the upper corona, on the other hand, $d_{\mathrm {p}}\sim 230 \,\mathrm {m}$ and $d_{\mathrm {e}}\sim 5\,\mathrm {m}$. In processes that occur on length scales greater than $d_{\mathrm {p}}$ and timescales greater than $\varPi _{\varOmega _{\mathrm {p}}}$, protons exhibit a *magnetized behavior*, which means that their trajectory is closely tied to the magnetic field lines, following a quasi-helical gyration pattern with the frequency given in Eq. (3). Likewise, electrons exhibit magnetized behavior in processes that occur on length scales greater than $d_{\mathrm {e}}$ and timescales greater than $\varPi _{\varOmega _{\mathrm {e}}}$.

An important length scale associated with electrostatic effects is the *Debye length*

10 $$\begin{aligned} \lambda _j\equiv \sqrt{\frac{k_{\mathrm {B}}T_j}{4\pi n_{0j} q_j^2}}, \end{aligned}$$

where $T_j$ is the (scalar, isotropic) temperature of species *j*. We note that $\lambda _{\mathrm {p}}\sim \lambda _{\mathrm {e}}$ through much of the heliosphere, which makes the Debye length unique among the scales we discuss. The total Debye length

11 $$\begin{aligned} \lambda _{\mathrm {D}}\equiv \left( \sum \limits _j\frac{1}{\lambda _j}\right) ^{-1} \end{aligned}$$

is the characteristic exponential decay length for a time-independent global electrostatic potential in a plasma. In the solar wind at 1 au, $\lambda _{\mathrm {p}}\sim \lambda _{\mathrm {e}}\sim 12\,\mathrm {m}$, while the plasma in the upper corona exhibits $\lambda _{\mathrm {p}}\sim \lambda _{\mathrm {e}}\sim 7\,\mathrm {cm}$. Collective plasma processes (i.e., particles behaving as if they only interact with a smooth macroscopic electromagnetic field rather than with individual moving charges) become important if the number of particles within a sphere of radius $\lambda _{\mathrm {D}}$ is large,

12 $$\begin{aligned} n_{0\mathrm {e}}\lambda _{\mathrm {D}}^3\gg 1, \end{aligned}$$

and if

13 $$\begin{aligned} \lambda _{\mathrm {D}}\ll L. \end{aligned}$$

Equations (12) and (13) guarantee that electrostatic single-particle effects are shielded by neighboring charges from the surrounding plasma (known as *Debye shielding*). If one or both of these conditions are not fulfilled, common plasma-physics methods do not apply and a material is merely an ionized gas rather than a plasma. The solar wind, however, satisfies both of these conditions and, therefore, is a plasma.

In addition to these collective plasma length scales and timescales, collisional effects are associated with their own characteristic scales, which depend on the type of collisional interaction under consideration (e.g., temperature equilibration or isotropization) and on different combinations of plasma parameters. We discuss these effects and the associated timescales in Sect. 3.

Comparing the coronal electron Debye length as the smallest plasma length scale of the solar wind with the size of the system reveals that the solar wind covers over twelve orders of magnitude in its characteristic length scales (neglecting length scales associated with collisions, which can be even greater than *L*). Similarly, comparing the corona’s electron plasma period with the solar wind’s expansion time reveals that the solar wind also covers over twelve orders of magnitude in its characteristic timescales (again neglecting timescales associated with collisions, which can be even greater than $\tau$). These ratios demonstrate the *intrinsically multi-scale nature of the solar wind*. The broad range of scales also illustrates the difficulty in treating the solar wind and all related physics processes numerically since complete numerical simulations would need to resolve this entire range of scales.

This review describes plasma processes that depend upon or modify the multi-scale nature of the solar wind. As a truly Living Review, its first edition is limited to small-scale processes that affect the large-scale evolution of the plasma. In a later major update, we will describe how large-scale processes affect the small-scale structure of the plasma such as expansion effects on particle properties, wave reflection and the creation of turbulence, streaming interactions, mixing from different solar sources in co-rotating interaction regions, and magnetic focusing effects, as well as the impact of these processes on global solar-wind modeling. Although every plasma process is conceivably a multi-scale process, we, by practical necessity, only address the physics processes we consider most relevant to the multi-scale evolution of the solar wind. The most prominent processes *not* covered in this review include detailed discussions of reconnection (Pontin 2011; Gosling 2012; Paschmann et al. 2013), shock waves (Balogh et al. 1995; Chashei and Shishov 1997; Lepping 2000; Rice and Zank 2003), the physics of the outer heliosphere (pick-up ions, energetic neutral atoms, etc., Zank et al. 1995; Gloeckler and Geiss 1998; Zank 1999; Richardson et al. 2004; McComas et al. 2012; Zank et al. 2018), interplanetary dust (Krüger et al. 2007; Mann et al. 2010), interactions with planetary bodies (Grard et al. 1991; Kivelson and Bagenal 2007; Gardini et al. 2011; Bagenal 2013), eruptive events such as coronal mass ejections (Zurbuchen and Richardson 2006; Howard and Tappin 2009; Webb and Howard 2012), solar energetic particles (Ryan et al. 2000; Mikić and Lee 2006; Klein and Dalla 2017), and (anomalous) cosmic rays (Heber et al. 2006; Potgieter 2008; Giacalone et al. 2012; Potgieter 2013). We also limit our discussion of minor-ion physics.

### Global structure of the solar wind

**Fig. 2:**
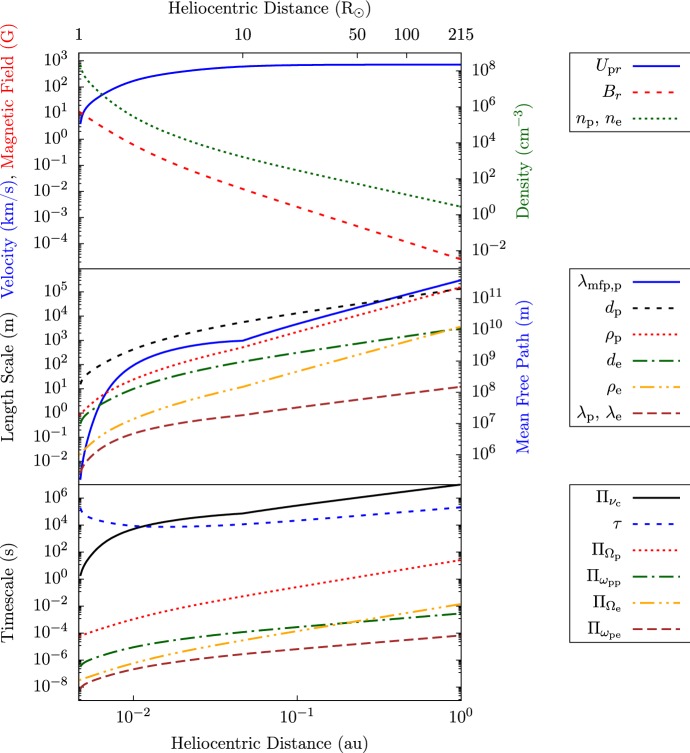
Characteristic average quantities, length scales, and timescales as functions of distance from the Sun in the inner heliosphere for typical fast-solar-wind conditions. We calculate these scales based on typical radial profiles of the solar-wind magnetic-field strength, density, and velocity (shown in the top panel). The profiles for the magnetic field and the density are taken from Smith et al. (2012) for a radial polar flux tube. The radial velocity profile then follows from flux conservation, $n_{j}U_{jr}/B_r=\mathrm {constant}$. The electron temperature is taken from a fit to measurements at $r< 10\,R_{\odot }$ (Cranmer et al. 1999) and then connected to a power-law with a power index corresponding to the radial temperature profiles observed with Helios in the fast solar wind (Štverák et al. 2015). We take $T_{\mathrm {p}}\approx T_{\mathrm {e}}$ for simplicity

At heliocentric distances greater than a few solar radii $R_{\odot }$, the solar wind’s expansion is, to first order, radial, which creates large-scale radial gradients in most of the plasma parameters. For this discussion of the global structure, we concentrate only on long-term averages of the plasma quantities and neglect their frequent—and, as we will see later, sometimes comparable to order unity—variations. Figure 2 illustrates these average quantities as functions of distance in the inner heliosphere and demonstrates the resulting profiles for the characteristic length scales and timescales. Beyond a distance of about $10\,R_{\odot }$, the average radial velocity stays approximately constant. Continuity under steady-state conditions requires that

14 $$\begin{aligned} \nabla \cdot \left( n_{j}\mathbf{U}_{j}\right) = 0, \end{aligned}$$

where $\mathbf{U}_j$ is the bulk velocity of species *j*. In spherical coordinates and under the assumption that $\mathbf{U}_j\approx U_{jr} \hat{\mathbf{e}}_r\approx \mathrm {constant}$, the average density then decreases $\propto r^{-2}$. In the acceleration region and in regions of super-radial expansion connected to coronal holes, continuity requires steeper gradients closer to the Sun as confirmed by white-light polarization measurements (Cranmer and van Ballegooijen 2005). In addition, the deceleration of streaming $\alpha$-particles leads to a small deviation from the $r^{-2}$ density profile (Verscharen et al. 2015).

To first order, the average magnetic field follows the Parker spiral in the plane of the ecliptic (Parker 1958; Levy 1976; Behannon 1978; Mariani et al. 1978, 1979) as a result of the frozen-in condition of ideal magnetohydrodynamics (MHD; see Sect. 1.4.2) and the rotation of the Sun. We define

15 $$\begin{aligned} \beta _{j}\equiv \frac{8\pi n_{j} k_{\mathrm {B}}T_j}{B^2}, \end{aligned}$$

where *B* is the magnetic field, as the ratio between the thermal pressure of species *j* and the magnetic pressure. In the solar corona, $\beta _j\ll 1$, so that the magnetic field constraints the plasma to co-rotate with the Sun. However, the magnetic field’s torque on the plasma decreases with distance from the Sun until the plasma outflow dominates the evolution of the magnetic field and convects the field into interplanetary space (Weber and Davis 1967). In the Parker model, the *Parker angle* $|\phi _{Br}|$ between the direction of the magnetic field and the radial direction increases with distance *r* from the Sun,

16 $$\begin{aligned} \tan \,\phi _{Br}=\frac{B_{\phi }}{B_r}=\frac{\varOmega _{\odot }\sin \theta }{U_{\mathrm {p} r}}\left( r_{\mathrm {eff}}-r\right) , \end{aligned}$$

where $B_{\phi }$ and $B_{r}$ are the azimuthal and radial components of the magnetic field, $\varOmega _{\odot }$ is the angular speed of the Sun’s rotation, $\theta$ is the polar angle, and $r_{\mathrm {eff}}$ is the effective co-rotation radius. In our sign and coordinate convention, $\phi _{Br}\le 0$ if $B_r>0$ since the Sun rotates in the ${+}\,\hat{\mathbf{e}}_{\phi }$-direction, which differs from Parker’s (1958) original choice. The radius $r_{\mathrm {eff}}$ is an auxiliary quantity to describe the heliospheric distance beyond which the solar wind behaves as if it were co-rotating for $r\le r_{\mathrm {eff}}$ (Hollweg and Lee 1989). Observations indicate that $r_{\mathrm {eff}}\sim 10\,R_{\odot }$ in the fast wind and $r_{\mathrm {eff}}\sim 20\,R_{\odot }$ in the slow wind (Bruno and Bavassano 1997). The Parker angle $|\phi _{Br}|$ increases from $0^{\circ }$ at $r_{\mathrm {eff}}$ to about $45^{\circ }$ at $r=1\,\mathrm {au}$. This trend continues into the outer heliosphere as shown by observations (Thomas and Smith 1980; Forsyth et al. 2002). The magnitude of the *Parker field* decreases with distance as

17 $$\begin{aligned} B_0\propto \frac{\sqrt{1+\tan ^2\,\phi _{Br}}}{r^2}, \end{aligned}$$

which is $\propto r^{-2}$ in the limit $\tan ^2\phi _{Br}\ll 1$ at small *r* and $\propto r^{-1}$ in the limit $\tan ^2\phi _{Br}\gg 1$ at large *r*. We note that the original Parker model is not completely torque-free, although a torque-free treatment leads to only minor modifications (Verscharen et al. 2015). Further details about the heliospheric magnetic field can be found in the review by Owens and Forsyth (2013).

### Categorization of solar wind

Traditionally, the solar wind has been categorized into three groups (Srivastava and Schwenn 2000):

1. *fast wind* with bulk velocities between about 500 km/s and 800 km/s,
2. *slow wind* with bulk velocities between about 300 km/s and 500 km/s, and
3. variable/eruptive events such as *coronal mass ejections* with speeds from a few hundreds up to 2000 km/s.

**Fig. 3:**
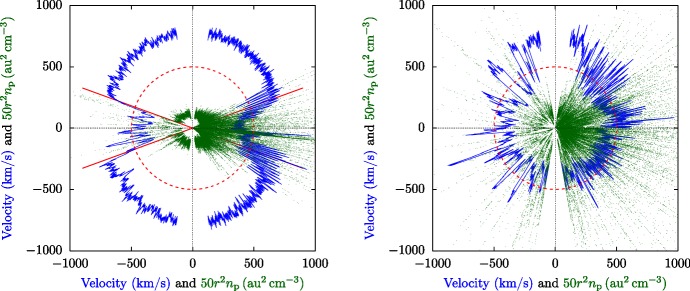
Ulysses/SWOOP observations of the solar-wind proton radial velocity and density at different heliographic latitudes. The distance from the center in each of these polar plots indicates the velocity (blue) and density (green). The polar angle represents the heliographic latitude. Since these measurements were taken at varying distances from the Sun, we compensate for the density’s radial decrease by multiplying $n_{\mathrm {p}}$ with $r^2$. The red circle represents $U_{\mathrm {p}r}=500\,\mathrm {km/s}$ and $r^2n_{\mathrm {p}}=10\,\mathrm {au}^2\,\mathrm {cm}^{-3}$. The straight red lines indicate the sector boundaries at $\pm 20^{\circ }$ latitude. Left panel: Ulysses’ first polar orbit during solar minimum (1990-12-20 through 1997-12-15). Right panel: Ulysses’ second polar orbit during solar maximum (1997-12-15 through 2004-02-22). After McComas et al. (2000) and McComas et al. (2008)

Measurements from the Ulysses spacecraft during solar minimum dramatically demonstrate that the fast wind emerges predominantly from polar coronal holes and the slow wind from the streamer belt at the solar equator (Phillips et al. 1995; McComas et al. 1998b, 2000, 2003; Ebert et al. 2009). The left-hand panel in Fig. 3 illustrates the clear sector boundary between fast and slow wind during solar minimum. During solar maximum, however, fast and slow wind emerge from neighboring patches everywhere in the corona. The right-hand panel in Fig. 3 shows that the occurrence of fast and slow wind streams does not strongly correlate with heliographic latitude during solar maximum. On average, fast polar wind exhibits both a lower density and less variation in density than slow wind. The association of different wind streams with different source regions suggests that the magnetic-field configuration in the corona plays a crucial role in determining the properties of the wind streams. In addition to the differences in speed and density, fast and slow wind exhibit further distinguishing marks. Fast wind, relative to slow wind, generally is more steady, is more Alfvénic (i.e., it exhibits a higher correlation or anti-correlation between fluctuations in vector velocity and vector magnetic field; see Sect. 4 and Tu and Marsch 1995), and has a higher proton temperature (Neugebauer 1976; Wilson et al. 2018). Importantly for its multi-scale evolution, fast wind is also less collisional (both in terms of the local collisional relaxation times and the cumulative time for collisions to act) than slow wind (Marsch et al. 1982b; Marsch and Goldstein 1983; Livi et al. 1986; Kasper et al. 2008; Bourouaine et al. 2011; Ďurovcová et al. 2017), which allows for more kinetic non-equilibrium features to survive the thermalizing action of Coulomb collisions. Fast wind, therefore, exhibits more non-Maxwellian structure in its distribution functions (Marsch 2006, 2018) as we discuss in the next section.

The elemental composition and the heavy-ion charge states also differ between fast and slow wind (Bame et al. 1975; Ogilvie and Coplan 1995; von Steiger et al. 1995; Bochsler 2000; von Steiger et al. 2000; Aellig et al. 2001b; Zurbuchen et al. 2002; Kasper et al. 2007, 2012; Lepri et al. 2013). Elements with a low *first ionization potential (FIP)* such as magnesium, silicon, and iron exhibit enhanced abundances in the solar corona and in the solar wind with respect to their photospheric abundances (Gloeckler and Geiss 1989; Raymond 1999; Laming 2015). Conversely, elements with a high FIP such as oxygen, neon, and helium have much lower enhancements or even depletions with respect to their photospheric abundances. This FIP fractionation bias also varies with wind speed and is generally smaller in fast wind than in slow wind (Zurbuchen et al. 1999; Bochsler 2007). Since the elemental composition of a plasma parcel does not change as it propagates through the heliosphere unless it mixes with neighboring parcels, composition measurements are a reliable method to distinguish solar-wind source regions. Moreover, studies of heavy ions constrain proposed models of solar-wind acceleration and heating. For instance, proposed acceleration and heating scenarios must explain the observed preferential heating of minor ions. In the solar wind, most heavy ion species *i* exhibit $T_i/T_{\mathrm {p}}\approx 1.35 m_i/m_{\mathrm {p}}$ (Tracy et al. 2015; Heidrich-Meisner et al. 2016; Tracy et al. 2016).

Lately, the traditional classification of wind streams by speed has experienced some major criticism (e.g., Maruca et al. 2013; Xu and Borovsky 2015; Camporeale et al. 2017). Speed alone does not fully classify the properties of the wind, and there is a smooth transition in the distribution of wind speeds. At times, fast solar wind shows properties traditionally associated with slow wind and vice versa, such as collisionality, Alfvénicity, FIP-bias, anisotropy, beam structures, etc. Although these atypical behaviors suggest a false dichotomy between fast and slow wind, we retain the traditional nomenclature, albeit defining “fast wind” as wind with the typical fast-wind properties and “slow wind” as wind with the typical slow-wind properties under consideration instead of relying on the flow speeds alone. Nevertheless, we expressly caution the reader against assuming wind speed alone as a reasonable indication of wind type.

### Kinetic properties of the solar wind

Kinetic plasma physics describes the statistical properties of a plasma by means of the *particle velocity distribution functions* $f_j(\mathbf{x},\mathbf{v},t)$ for each plasma species *j*. We define and normalize the distribution function so that

18 $$\begin{aligned} f_j(\mathbf{x},\mathbf{v},t)\,\mathrm {d}^3\mathbf{x}\,\mathrm {d}^3\mathbf{v} \end{aligned}$$

represents the number of particles of species *j* in the phase-space volume $\mathrm {d}^3\mathbf{x}\,\mathrm {d}^3\mathbf{v}$ centered on the phase-space coordinates $(\mathbf{x},\mathbf{v})$ at time *t*. The distribution function relates to the bulk properties (i.e., density, bulk velocity, temperature,...) through its velocity moments as described in Sect. 1.4.1. A continuous definition of $f_j$ is appropriate when Eq. (12) is fulfilled.

The central equation in kinetic physics is the *Boltzmann equation*,

19 $$\begin{aligned} \frac{\partial f_j}{\partial t}+\mathbf{v}\cdot \frac{\partial f_j}{\partial \mathbf{x}}+\mathbf{a}\cdot \frac{\partial f_j}{\partial \mathbf{v}}=\left( \frac{\delta f_j}{\delta t}\right) _{\mathrm {c}}, \end{aligned}$$

where $\mathbf{a}$ is the acceleration of a *j*-particle due to macroscopic forces, and the right-hand side describes the temporal change in $f_j$ due to particle collisions, which are mediated by microscopic electric forces among individual particles (see also Sect. 3.2 of this review; Lifshitz and Pitaevskii 1981). We use the term *macroscopic fields* to indicate that these are locally averaged to remove the rapidly fluctuating Coulomb electric fields due to individual charges, which are responsible for Coulomb collisions. The applicability of this mean-field approach is a key quality of a plasma and distinguishes it from other types of ionized gases, in which Eq. (12) is not fulfilled. Without the collision term, the Boltzmann equation represents a fluid continuity equation for the density in phase space. It is thus related to *Liouville’s theorem* and describes the conservation of the phase-space density along trajectories in the absence of collisions.^2^ In this case, and when using only macroscopic electromagnetic forces in the acceleration term, we obtain the *Vlasov equation*,

20 $$\begin{aligned} \frac{\partial f_j}{\partial t}+\mathbf{v}\cdot \frac{\partial f_j}{\partial \mathbf{x}}+\frac{q_j}{m_j}\left( \mathbf{E}+\frac{1}{c}\mathbf{v}\times \mathbf{B}\right) \cdot \frac{\partial f_j}{\partial \mathbf{v}}=0, \end{aligned}$$

which is the fundamental equation of collisionless kinetic plasma physics. These macroscopic electric and magnetic fields obey *Maxwell’s equations*,

21 $$\begin{aligned} \nabla \cdot \mathbf{E}&=4\pi \rho _{\mathrm {c}}, \end{aligned}$$

22 $$\begin{aligned} \nabla \cdot \mathbf{B}&=0, \end{aligned}$$

23 $$\begin{aligned} \nabla \times \mathbf{E}&=-\frac{1}{c}\frac{\partial \mathbf{B}}{\partial t}, \end{aligned}$$

and

24 $$\begin{aligned} \nabla \times \mathbf{B}=\frac{4\pi }{c}\mathbf{j}+\frac{1}{c}\frac{\partial \mathbf{E}}{\partial t}, \end{aligned}$$

where the charge density $\rho _{\mathrm {c}}$ and the current density $\mathbf{j}$ are given by integrals over the distribution functions as

25 $$\begin{aligned} \rho _{\mathrm {c}}=\sum \limits _j q_j \int f_j\,\mathrm {d}^3\mathbf{v} \end{aligned}$$

and

26 $$\begin{aligned} \mathbf{j}=\sum \limits _j q_j \int \mathbf{v} f_j\,\mathrm {d}^3\mathbf{v}. \end{aligned}$$

Equations (20) through (26) form a closed set of integro-differential equations in six-dimensional phase space and time that fully describe the evolution of collisionless plasma.

#### Fluid moments and fluid equations

Although the distribution functions $f_j$ contain all of the microphysical properties of the plasma, it is often sufficient to rely on a reduced set of macrophysical parameters that only depend on time and three-dimensional configuration space (versus time and six-dimensional phase space). These parameters are called *bulk parameters* and correspond to the velocity moments as integrals over the full velocity space of the distribution function. Certain velocity moments represent named fluid bulk parameters. For instance, the zeroth velocity moment corresponds to the *number density*

27 $$\begin{aligned} n_j=\int f_j\,\mathrm {d}^3\mathbf{v}. \end{aligned}$$

Using $n_j$, the first velocity moment corresponds to the *bulk velocity*

28 $$\begin{aligned} \mathbf{U}_j=\frac{1}{n_j}\int \mathbf{v} f_j\,\mathrm {d}^3\mathbf{v}, \end{aligned}$$

while the second moment represents the *pressure tensor*

29 $$\begin{aligned} {\mathsf {P}}_j=m_j\int \left( \mathbf{v}-\mathbf{U}_j\right) \left( \mathbf{v}-\mathbf{U}_j\right) f_j\,\mathrm {d}^3\mathbf{v}. \end{aligned}$$

The third moment corresponds to the *heat-flux tensor*

30 $$\begin{aligned} {\mathsf {Q}}_j=m_j\int \left( \mathbf{v}-\mathbf{U}_j\right) \left( \mathbf{v}-\mathbf{U}_j\right) \left( \mathbf{v}-\mathbf{U}_j\right) f_j\,\mathrm {d}^3\mathbf{v}. \end{aligned}$$

For many applications in magnetized-plasma physics, it is useful to choose the coordinate system to be aligned with the direction $\hat{\mathbf{b}}\equiv \mathbf{B}/|\mathbf{B}|$ of the magnetic field and to define the pressure components with respect to the direction of the magnetic field. In this coordinate system, Equation (30) reduces through contraction to the perpendicular heat-flux vector

31 $$\begin{aligned} \mathbf{q}_{\perp j}=\frac{1}{2}{\mathsf {Q}}_j: \left( {\mathsf {I}}_3-\hat{\mathbf{b}}\hat{\mathbf{b}}\right) \end{aligned}$$

and the parallel heat-flux vector

32 $$\begin{aligned} \mathbf{q}_{\parallel j}={\mathsf {Q}}_j : \left( \hat{\mathbf{b}}\hat{\mathbf{b}}\right) , \end{aligned}$$

where ${\mathsf {I}}_3$ is the three-dimensional unit matrix. We define the double-dot and triple-dot products in a similar way to the usual dot product as

33 $$\begin{aligned} {\mathsf {A}}:{\mathsf {B}}=\sum \limits _{i,j}{\mathsf {A}}_{ij}{\mathsf {B}}_{ji}\qquad \text {and} \qquad {\mathsf {A}} \dot{:} {\mathsf {B}}=\sum \limits _{i,j,k}{\mathsf {A}}_{ijk}{\mathsf {B}}_{kji}. \end{aligned}$$

Although higher moments do not give rise to named bulk parameters like these four, the *moment hierarchy* can be continued to infinity by multiplying the integrand with further powers of velocity.

Taking velocity moments of the full Vlasov equation and exploiting the definitions of the lowest moments above leads to the *multi-fluid plasma equations* (Barakat and Schunk 1982; Marsch 2006). The zeroth and first moments of the Vlasov equation are the *continuity equation*,

34 $$\begin{aligned} \frac{\partial n_j}{\partial t}+\nabla \cdot \left( n_j\mathbf{U}_j\right) = 0, \end{aligned}$$

and the *momentum equation*,

35 $$\begin{aligned} n_jm_j\left( \frac{\partial }{\partial t}+\mathbf{U}_j\cdot \nabla \right) \mathbf{U}_j=-\,\nabla \cdot {\mathsf {P}}_j+n_j q_j\left( \mathbf{E}+\frac{1}{c}\mathbf{U}_j\times \mathbf{B}\right) . \end{aligned}$$

We define the perpendicular pressure and the parallel pressure as

36 $$\begin{aligned} p_{\perp j}\equiv {\mathsf {P}}_j:\frac{{\mathsf {I}}_3-\hat{\mathbf{b}}\hat{\mathbf{b}}}{2} \end{aligned}$$

and

37 $$\begin{aligned} p_{\parallel j}\equiv {\mathsf {P}}_j:\left( \hat{\mathbf{b}}\hat{\mathbf{b}}\right) , \end{aligned}$$

respectively, which are related to the temperatures in the directions perpendicular and parallel to $\mathbf{B}$ through

38 $$\begin{aligned} T_{\perp j}=\frac{p_{\perp j}}{n_j k_{\mathrm {B}}} \end{aligned}$$

and

39 $$\begin{aligned} T_{\parallel j}=\frac{p_{\parallel j}}{n_j k_{\mathrm {B}}}. \end{aligned}$$

We write the *perpendicular energy equation* as

40 $$\begin{aligned}&\left( \frac{\partial }{\partial t}+\mathbf{U}_j\cdot \nabla \right) p_{\perp j}+p_{\perp j}\left( \nabla \cdot \mathbf{U}_j+\nabla _{\perp }\cdot \mathbf{U}_j\right) =\left( \hat{\mathbf{b}}\hat{\mathbf{b}}-{\mathsf {I}}_3\right) :\left( \varvec{\tau }_j\cdot \nabla \mathbf{U}_j\right) \nonumber \\&\quad -\nabla \cdot \mathbf{q}_{\perp j}-\frac{1}{2}\varvec{\tau }_j:\left( \frac{\partial }{\partial t}+\mathbf{U}_j\cdot \nabla \right) \left( \hat{\mathbf{b}}\hat{\mathbf{b}}\right) -\frac{1}{2}{\mathsf {Q}}_j \dot{:} \nabla \left( \hat{\mathbf{b}}\hat{\mathbf{b}}\right) \end{aligned}$$

and the *parallel energy equation* as

41 $$\begin{aligned}&\left( \frac{\partial }{\partial t}+\mathbf{U}_j\cdot \nabla \right) p_{\parallel j}+p_{\parallel j}\left( \nabla \cdot \mathbf{U}_j+2\nabla _{\parallel }\cdot \mathbf{U}_j\right) =-\,2\hat{\mathbf{b}}\hat{\mathbf{b}}:\left( \varvec{\tau }_j\cdot \nabla \mathbf{U}_j\right) \nonumber \\&\quad -\nabla \cdot \mathbf{q}_{\parallel j}+\varvec{\tau }_j:\left( \frac{\partial }{\partial t}+\mathbf{U}_j\cdot \nabla \right) \left( \hat{\mathbf{b}}\hat{\mathbf{b}}\right) +{\mathsf {Q}}_j \dot{:} \nabla \left( \hat{\mathbf{b}}\hat{\mathbf{b}}\right) , \end{aligned}$$

where

42 $$\begin{aligned} {\varvec{\tau }}_j\equiv {\mathsf {P}}_j-p_{\perp j}{\mathsf {I}}_3-\left( p_{\parallel j}-p_{\perp j}\right) \hat{\mathbf{b}}\hat{\mathbf{b}} \end{aligned}$$

is the stress tensor,

43 $$\begin{aligned} \nabla _{\perp }\equiv \left( {\mathsf {I}}_3-\hat{\mathbf{b}}\hat{\mathbf{b}}\right) \nabla ,\qquad \text {and}\qquad \nabla _{\parallel }\equiv \left( \hat{\mathbf{b}}\hat{\mathbf{b}}\right) \nabla . \end{aligned}$$

The hierarchy of moments of the Vlasov equation continues to infinity, and similar fluid equations exist for the stress tensor, the heat-flux tensor, and all higher-order moments. However, this gives rise to a closure problem since the *n*th moment of the Vlasov equation always includes the $(n+1)$st moment of the distribution function. For example, the continuity equation, which is the zeroth moment of the Vlasov equation, includes the bulk velocity, which corresponds to the first moment of $f_j$. The $(n+1)$st moment of the distribution function, in turn, requires the $(n+1)$st moment of the Vlasov equation as a description of its dynamical evolution. Every fluid model is, therefore, fundamentally susceptible to a closure problem since the solution of an infinite chain of non-degenerate equations is formally impossible. For most practical purposes, the moment hierarchy is thus truncated by expressing a higher-order moment of $f_j$ through lower moments of $f_j$ only. Closing the moment hierarchy introduces limitations on the physics of the problem at hand and deviations in the solutions to the multi-fluid system of equations from the solutions to the full Vlasov equation. For example, a typical closure of the moment hierarchy is the assumption of an isotropic and adiabatic pressure, i.e., ${\mathsf {P}}_j=p_j\, {\mathsf {I}}_3$ and $p_j\propto n_j^{\kappa }$, where $\kappa$ is the adiabatic exponent. This closure of the momentum equation neglects heat flux and small velocity-space structure in $f_j$. Therefore, any finite closure is only applicable if the physics of the problem at hand justifies the neglect of higher-order velocity moments of $f_j$. We note, for example, that collisions are such a process that can produce conditions under which higher-order moments are negligible (see Sect. 3).

Assuming only slow changes of the magnetic field compared to $\varPi _{\varOmega _j}$ and that $\varvec{\tau }_j=0$, the second velocity moment of the Vlasov equation (20) leads to the useful *double-adiabatic energy equations* (Chew et al. 1956; Whang 1971; Sharma et al. 2006; Chandran et al. 2011),

44 $$\begin{aligned} n_j B\left( \frac{\partial }{\partial t}+\mathbf{U}_j\cdot \nabla \right) \left( \frac{p_{\perp j}}{n_jB}\right) =-\,\nabla \cdot \mathbf{q}_{\perp j} -q_{\perp j}\nabla \cdot \hat{\mathbf{b}} \end{aligned}$$

and

45 $$\begin{aligned} \frac{n_j^3}{B^2}\left( \frac{\partial }{\partial t}+\mathbf{U}_j\cdot \nabla \right) \left( \frac{B^2p_{\parallel j}}{n_j^3}\right) =-\,\nabla \cdot \mathbf{q}_{\parallel j}+2q_{\perp j}\nabla \cdot \hat{\mathbf{b}}. \end{aligned}$$

If we neglect heat flux by setting the right-hand sides of Eqs. (44) and (45) to zero, we obtain the conservation laws for the *double-adiabatic invariants*, which are also referred to as the *Chew–Goldberger–Low (CGL) invariants* (Chew et al. 1956)

46 $$\begin{aligned} \frac{p_{\perp j}}{n_jB}\approx \mathrm {constant}\qquad \text {and} \qquad \frac{B^2p_{\parallel j}}{n_j^3}\approx \mathrm {constant.} \end{aligned}$$

#### Magnetohydrodynamics

Magnetohydrodynamics (MHD) is a single-fluid description that results from summing the fluid equations of all species and defining the moments of the single *magnetofluid* as the mass density

47 $$\begin{aligned} \rho \equiv \sum \limits _jm_jn_j, \end{aligned}$$

the bulk velocity

48 $$\begin{aligned} \mathbf{U}\equiv \frac{1}{\rho }\sum \limits _j m_jn_j\mathbf{U}_j, \end{aligned}$$

and the total scalar pressure

49 $$\begin{aligned} P\equiv \frac{1}{3} \sum \limits _j {\mathsf {P}}_j : {\mathsf {I}}_3 \end{aligned}$$

under the assumption that ${\mathsf {P}}_j$ is isotropic and diagonal. This procedure leads to the *MHD continuity equation*,

50 $$\begin{aligned} \frac{\partial \rho }{\partial t}+\nabla \cdot \left( \rho \mathbf{U}\right) =0, \end{aligned}$$

and the *MHD momentum equation*,

51 $$\begin{aligned} \rho \left( \frac{\partial }{\partial t}+\mathbf{U}\cdot \nabla \right) \mathbf{U}=-\,\nabla P+\frac{1}{c}\left( \mathbf{j}\times \mathbf{B}\right) . \end{aligned}$$

The electric-field term from Eq. (35) vanishes under the quasi-neutrality assumption that $\rho _{\mathrm {c}}$ from Eq. (25) is negligible, which is justified on scales $\gg \lambda _{\mathrm {D}}$. Faraday’s law describes the evolution of the magnetic field as

52 $$\begin{aligned} \frac{\partial \mathbf{B}}{\partial t}=-\,c\nabla \times \mathbf{E}. \end{aligned}$$

The electric field follows from the electron momentum equation (35) as the generalized Ohm’s law,

53 $$\begin{aligned} \mathbf{E}=\frac{m_{\mathrm {e}}}{q_{\mathrm {e}}}\left( \frac{\partial }{\partial t}+\mathbf{U}_{\mathrm {e}}\cdot \nabla \right) \mathbf{U}_{\mathrm {e}}+\frac{1}{n_{\mathrm {e}}q_{\mathrm {e}}}\nabla \cdot {\mathsf {P}}_{\mathrm {e}}-\frac{1}{n_{\mathrm {e}}q_{\mathrm {e}}c}\mathbf{j}\times \mathbf{B}+\frac{1}{n_{\mathrm {e}}q_{\mathrm {e}}c}\mathbf{j}_{\mathrm {i}}\times \mathbf{B}, \end{aligned}$$

where

54 $$\begin{aligned} \mathbf{j}_{\mathrm {i}}\equiv \mathbf{j}-n_{\mathrm {e}}q_{\mathrm {e}}\mathbf{U}_{\mathrm {e}} \end{aligned}$$

is the ion contribution to the current density. The terms on the right-hand side of Eq. (53) represent the contributions from electron inertia, the electron pressure gradient (i.e., the ambipolar electric field), the Hall term, and the ion convection term, respectively. Under the assumptions of quasi-neutrality in a proton–electron plasma and the negligibility of terms of order $m_{\mathrm {e}}/m_{\mathrm {p}}$, we find

55 $$\begin{aligned} \mathbf{E}=\frac{1}{n_{\mathrm {e}}q_{\mathrm {e}}}\nabla \cdot {\mathsf {P}}_{\mathrm {e}}-\frac{1}{n_{\mathrm {e}}q_{\mathrm {e}}c}\mathbf{j}\times \mathbf{B}-\frac{1}{c}\mathbf{U}\times \mathbf{B}. \end{aligned}$$

If we furthermore assume small or moderate $\beta _{\mathrm {e}}$ and consider processes occurring on scales $\gg d_{\mathrm {p}}$ (Chiuderi and Velli 2015), we can neglect the contributions of the electron pressure gradient and the Hall term to $\mathbf{E}$. We then find the common expression for Ohm’s law in MHD:

56 $$\begin{aligned} \mathbf{E}=-\frac{1}{c}\mathbf{U}\times \mathbf{B}. \end{aligned}$$

Equations (52) and (56) describe *Alfvén’s frozen-in theorem*, stating that magnetofluid bulk motion across field lines is forbidden, since otherwise the infinite resistivity of the magnetofluid would lead to infinite eddy currents. Instead, the magnetic flux through a co-moving surface is conserved.^3^ The assumptions leading to Eq. (56) are fulfilled for processes on time scales much greater than $\varPi _{\varOmega _{j}}$ and $\varPi _{\omega _{\mathrm {p}j}}$ as well as on spatial scales much greater than $d_{j}$ and $\rho _{j}$. In this limit, the displacement current in Ampère’s law is also negligible, which allows us to write the current density in Eq. (51) in terms of the magnetic field:

57 $$\begin{aligned} \mathbf{j}=\frac{c}{4\pi }\nabla \times \mathbf{B}. \end{aligned}$$

The MHD equations are often closed with the adiabatic closure relation,

58 $$\begin{aligned} \left( \frac{\partial }{\partial t}+\mathbf{U}\cdot \nabla \right) \left( \frac{P}{\rho ^\kappa }\right) =0, \end{aligned}$$

where $\kappa$ is the adiabatic exponent. The MHD equations are intrinsically scale-free and, therefore, only valid for processes that do not occur on any of the characteristic plasma scales of the system introduced in Sect. 1.1. Thus, MHD only applies to large-scale phenomena that occur

1. on length scales $\lesssim \,L$,
2. on length scales $\gg \max (d_{j},\rho _j)$, and
3. on timescales $$\gg \max (\varPi _{\varOmega _j}, \varPi _{\omega _{\mathrm {p}j}})$$

for all *j*.

#### Standard distributions in solar-wind physics

Although solar-wind measurements often reveal irregular plasma distribution functions (see Sects. 1.4.4, 1.4.5, as well as Marsch 2012), it is sometimes helpful to invoke closed analytical expressions for the distribution functions in a plasma. In the following description, we use the cylindrical coordinate system in velocity space introduced in Sect. 1.4.1 with its symmetry axis to be parallel to $\hat{\mathbf{b}}$.

A gas in thermodynamic equilibrium has a *Maxwellian* velocity distribution,

59 $$\begin{aligned} f_{\mathrm {M}}(\mathbf{v})=\frac{n_j}{\pi ^{3/2}w_j^3}\exp \left( -\frac{\left( \mathbf{v}-\mathbf{U}_j\right) ^2}{w_j^2}\right) , \end{aligned}$$

where

60 $$\begin{aligned} w_j\equiv \sqrt{\frac{2k_{\mathrm {B}}T_j}{m_j}} \end{aligned}$$

is the (isotropic) thermal speed of species *j*. Equation (59) has a thermodynamic justification in equilibrium statistical mechanics based on the Gibbs distribution (Landau and Lifshitz 1969). An empirically motivated extension of the Maxwellian distribution is the so-called *bi-Maxwellian* distribution, which introduces temperature anisotropies with respect to the background magnetic field yet follows the Maxwellian behavior on any one-dimensional cut at constant $v_{\perp }$ or constant $v_{\parallel }$ in velocity space:

61 $$\begin{aligned} f_{\mathrm {bM}}(\mathbf{v})=\frac{n_j}{\pi ^{3/2}w_{\perp j}^2w_{\parallel j}}\exp \left( -\frac{v_{\perp }^2}{w_{\perp j}^2}-\frac{\left( v_{\parallel }-U_{\parallel j}\right) ^2}{w_{\parallel j}^2}\right) , \end{aligned}$$

where $w_{\perp j}$ and $w_{\parallel j}$ are the thermal speeds defined in Eqs. (4) and (5). Advanced methods in thermodynamics such as non-extensive statistical mechanics lead to the $\kappa$-*distribution* (Tsallis 1988; Livadiotis and McComas 2013; Livadiotis 2017),

62 $$\begin{aligned} f_{\kappa }(\mathbf{v})=\frac{n_j}{w_j^3}\left[ \frac{2}{\pi (2\kappa -3)}\right] ^{3/2}\frac{\varGamma (\kappa +1)}{\varGamma (\kappa -1/2)}\left[ 1+\frac{2}{2\kappa -3}\frac{\left( \mathbf{v}-\mathbf{U}_j\right) ^2}{w_j^2}\right] ^{-\kappa -1}, \end{aligned}$$

where $\varGamma (x)$ is the $\varGamma$-function (Abramowitz and Stegun 1972) and $\kappa >3/2$. We note that $f_{\kappa }\rightarrow f_{\mathrm {M}}$ for $\kappa \rightarrow \infty$. The $\kappa$-distribution is characterized by having tails that are more pronounced for smaller $\kappa$ (i.e., the kurtosis of the distribution increases as $\kappa$ decreases). Analogous to the bi-Maxwellian is the *bi-*$\kappa$*-distribution*,

63 $$\begin{aligned} f_{\mathrm {b}\kappa }(\mathbf{v})= & {} \frac{n_j}{w_{\perp j}^2w_{\parallel j}}\left[ \frac{2}{\pi (2\kappa -3)}\right] ^{3/2}\frac{\varGamma (\kappa +1)}{\varGamma (\kappa -1/2)}\nonumber \\&\times \left\{ 1+\frac{2}{2\kappa -3}\left[ \frac{v_{\perp }^2}{w_{\perp j}^2}+\frac{\left( v_{\parallel }-U_{\parallel j}\right) ^2}{w_{\parallel j}^2}\right] \right\} ^{-\kappa -1}. \end{aligned}$$

In the following sections, we will encounter observed distribution functions and recognize some of the uses and limitations of these analytical expressions.

#### Ion properties

**Fig. 4:**
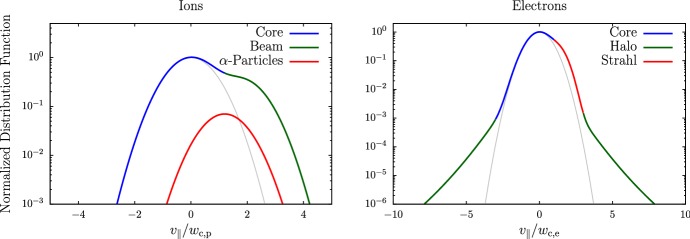
Illustration of ion (left) and electron (right) kinetic features in the solar wind. We show cuts through the distribution function along the direction of the magnetic field. We normalize the distribution functions to the maxima of the proton and electron distribution functions, respectively. We normalize the parallel velocity to the thermal speed of the proton and electron core components, $w_{\mathrm {c,p}}$ and $w_{\mathrm {c,e}}$, respectively. We note that $w_{\mathrm {c,p}}\ll w_{\mathrm {c,e}}$. The gray curves show the underlying core distribution alone. The distributions are shown in the reference frames in which the core distribution is at rest

In-situ spacecraft instrumentation has been measuring ion and electron velocity distributions for decades (see Sect. 2.2). Figure 4 summarizes some of the observed features in ion and electron distribution functions schematically.

**Fig. 5:**
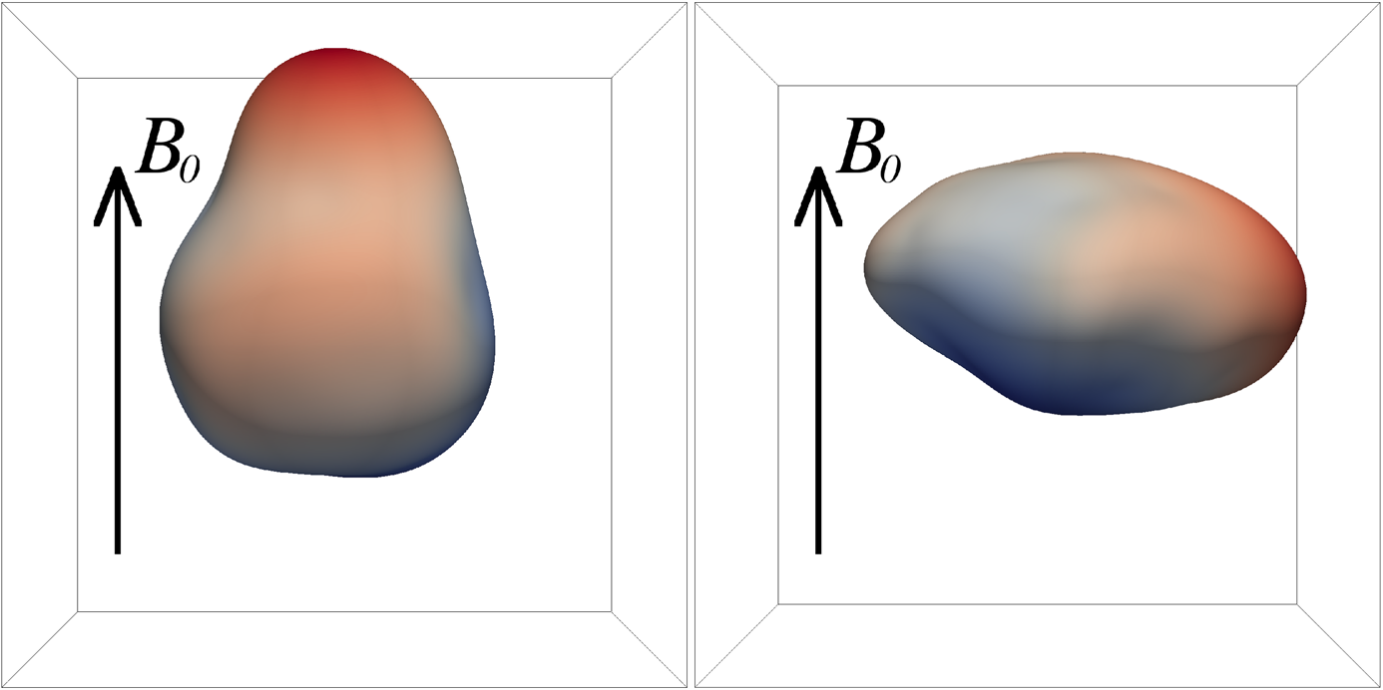
Interpolated isosurfaces in velocity space of two proton distribution functions measured by Helios 2. The arrow $\mathbf{B}_0$ indicates the direction of the local magnetic field. The color-coding represents the distance of the isosurface from the center-of-mass velocity. Left: measurement from 1976-02-04 at 10:21:43 UTC. The center-of-mass velocity is 478 km/s. The elongation along the magnetic-field direction represents the proton beam. Right: measurement from 1976-04-16 at 07:50:54 UTC. The center-of-mass velocity is 768 km/s. The oblate structure of the distribution function represents a temperature anisotropy with $T_{\perp \mathrm {p}}>T_{\parallel \mathrm {p}}$. These distribution functions are available as animations in the online supplementary material

These observations show that proton distributions often deviate from the Maxwellian equilibrium distribution given by Eq. (59). For instance, proton distributions often display a field-aligned *beam*: a second proton component streaming faster than the proton *core* component along the direction of the magnetic field with a relative speed $\gtrsim v_{\mathrm {Ap}}$ (Asbridge et al. 1974; Feldman et al. 1974b; Marsch et al. 1982b; Goldstein et al. 2000; Tu et al. 2004; Alterman et al. 2018). In Fig. 4 (left), the proton beam is shown in green as an extension of the distribution function toward greater $v_{\parallel }$. Protons also show *temperature anisotropies* with respect to the magnetic field (Hundhausen et al. 1967a, b; Marsch et al. 1981; Kasper et al. 2002; Marsch et al. 2004; Hellinger et al. 2006; Bale et al. 2009; Maruca et al. 2012), which manifest in unequal diagonal elements of ${\mathsf {P}}_j$ in Eq. (29). Figure 5 shows isosurfaces of $f_{\mathrm {p}}$ based on measurements from the Helios spacecraft. The background magnetic field is vertically aligned, and the color-coding represents the distance of the isosurfaces from the center-of-mass velocity. A standard Maxwellian distribution would be a monochromatic sphere in these diagrams. Instead, we see that the proton distribution is anisotropic. The example on the left-hand side shows an extension of the isosurface along the magnetic-field direction, which indicates the proton-beam component. Almost always, the proton beam is directed away from the Sun and along the magnetic-field axis.^4^ This observation suggests that the beam represents a preferentially accelerated proton component. The existence of this beam thus puts a major observational constraint on potential mechanisms for solar-wind heating and acceleration, which must generate this almost ubiquitous feature in $f_{\mathrm {p}}$. In the example on the right-hand side of Fig. 5, the isosurface is spread out in the directions perpendicular to the magnetic field, which indicates that $T_{\perp \mathrm {p}}>T_{\parallel \mathrm {p}}$. Although the plasma also exhibits periods with $T_{\perp \mathrm {p}}<T_{\parallel \mathrm {p}}$, the predominance of cases with $T_{\perp \mathrm {p}}>T_{\parallel \mathrm {p}}$ in the fast wind in the inner heliosphere (Matteini et al. 2007) suggests an ongoing heating mechanism in the solar wind that counter-acts the double-adiabatic expansion quantified in Eqs. (44) and (45). The double-adiabatic expansion alone would create $T_{\perp \mathrm {p}}\ll T_{\parallel \mathrm {p}}$ in the inner heliosphere when we neglect the action of heat flux and collisions on protons. Therefore, only heating mechanisms that explain the observed anisotropies with $T_{\perp \mathrm {p}}>T_{\parallel \mathrm {p}}$ in the solar wind (and possibly also in the corona; see Kohl et al. 2006) are successful candidates for a complete description of the physics of the solar wind.

The colors on the isosurfaces in Fig. 5 illustrate that the bulk velocity of the proton distribution function differs significantly from the center-of-mass velocity. This is mostly due to the $\alpha$-particles in the solar wind (Ogilvie 1975; Asbridge et al. 1976; Marsch et al. 1982a; Neugebauer et al. 1994, 1996; Steinberg et al. 1996; Reisenfeld et al. 2001; Berger et al. 2011; Gershman et al. 2012; Bourouaine et al. 2013). Although their number density is small ($n_{\alpha }\lesssim 0.05n_{\mathrm {p}}$), their mass density corresponds to about 20% of the proton mass density. We often observe the $\alpha$-particles, like the proton beam, to drift with respect to the proton core along the magnetic-field direction and away from the Sun with a typical drift speed $\lesssim \,v_{\mathrm {Ap}}$. In Fig. 4 (left), the $\alpha$-particles are shown as a separate shifted distribution in red, centered around the $\alpha$-particle drift speed.

The solar wind also exhibits *anisothermal behavior*; i.e., not all plasma species have equal temperatures (Formisano et al. 1970; Feldman et al. 1974a; Bochsler et al. 1985; Cohen et al. 1996; von Steiger and Zurbuchen 2002, 2006). The $\alpha$-particles often show $T_{\parallel \alpha }\gtrsim 4 T_{\parallel \mathrm {p}}$ (Kasper et al. 2007, 2008, 2012). Electrons are typically colder than protons in the fast solar wind but hotter than protons in the slow solar wind (Montgomery et al. 1968; Hundhausen 1970; Newbury et al. 1998). As stated in Sect. 1.2, heavy-ion-to-proton temperature ratios are typically greater than the corresponding heavy-ion-to-proton mass ratios for almost all observable ions in the solar wind. Like the other kinetic features, solar-wind heating and acceleration models are only fully successful if they explain the observed anisothermal behavior.

All of these non-equilibrium features (temperature anisotropies, beams, drifts, and anisothermal behavior) are less pronounced in the slow solar wind than in the fast wind, which is typically attributed to the greater collisional relaxation rates and the longer expansion times in the slow wind (see Sect. 3.3). These non-equilibrium features reflect the multi-scale nature of the solar wind, since they are driven by a combination of large-scale expansion effects, local kinetic processes, and the feedback of small-scale processes on the large-scale evolution.

#### Electron properties

Although the mass of an electron is much less than the mass of a proton ($m_{\mathrm {e}}/m_{\mathrm {p}}\approx 1/1836$), and the electrons’ contribution to the total solar-wind momentum flux is insignificant, electrons do affect the large-scale evolution of the solar wind (Montgomery 1972; Salem et al. 2003). As the most abundant particle species, they guarantee quasi-neutrality: $\rho _{\mathrm {c}}\approx 0$ and $j_{\parallel } \approx 0$ at length scales $\gg \lambda _{\mathrm {e}}$ and timescales $\gg \varPi _{\omega _{\mathrm {pe}}}$. Due to their small mass, they are highly mobile and have a much greater thermal speed than the protons, leading to their subsonic behavior (i.e., $U_{\mathrm {e}}\ll w_{\mathrm {e}}$). Their momentum balance in Eq. (35) is dominated by their pressure gradient and electromagnetic forces. Through these contributions, the electrons create an *ambipolar electrostatic field* in the expanding solar wind. This field is the central underlying acceleration mechanism of exospheric models (see Sect. 3.1; Lemaire and Scherer 1973; Maksimovic et al. 2001). Parker’s (1958) solar-wind model does not explicitly invoke an ambipolar electrostatic field. Nevertheless, the electron contribution to the pressure gradient in Parker’s MHD equation of motion is equivalent to the ambipolar electric field that follows from Eq. (35) for electrons in the limit $m_{\mathrm {e}}\rightarrow 0$ (Velli 1994, 2001).

Although electrons typically have greater collisional relaxation rates than ions, they exhibit a number of characteristic kinetic non-equilibrium features, which, as for the ions, are more pronounced in the fast solar wind. Most notably, the electron distribution often consists of three distinct components (Feldman et al. 1975; Pilipp et al. 1987a, b; Hammond et al. 1996; Maksimovic et al. 1997; Fitzenreiter et al. 1998):

- a thermal *core*, which mostly follows a Maxwellian distribution and has a thermal energy of $\sim 10\,\mathrm {eV}$—blue in Fig. 4 (right);
- a non-thermal *halo*, which mostly follows a $\kappa$-distribution, manifests as enhanced high-energy tails in the electron distribution, and has a thermal energy of $\lesssim \,80\,\mathrm {eV}$—green in Fig. 4 (right); and
- a *strahl*,^5^ which is a field-aligned beam of electrons and usually travels in the anti-Sunward direction with a bulk energy $\lesssim \,100\,\mathrm {eV}$—red in Fig. 4 (right).

The core typically includes $\sim 95\%$ of the electrons. It sometimes displays a temperature anisotropy (Serbu 1972; Phillips et al. 1989b; Štverák et al. 2008) and a relative drift with respect to the center-of-mass frame (Bale et al. 2013). A recent study suggests that a bi-self-similar distribution, which forms through inelastic particle scattering, potentially describes the core distribution better than a bi-Maxwellian distribution (Wilson et al. 2019).

**Fig. 6:**
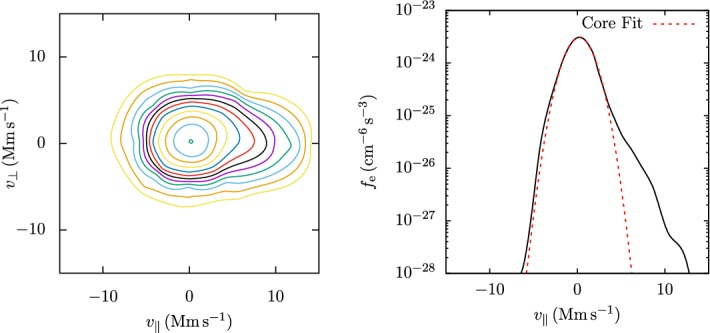
Electron velocity distribution function measured by Helios 2 in the fast solar wind at a heliocentric distance of $0.29\,\mathrm {au}$ on 1976-04-18 at 23:38:35 UTC. Left: isocontours of the distribution in a field-aligned coordinate system. Right: a cut through the distribution function along the magnetic-field direction. The red dashed curve shows a Maxwellian fit to the core of the distribution function. The strahl is clearly visible as an enhancement in the distribution function at $v_{\parallel }>0$

The strahl probably results from a more isotropic distribution of superthermal electrons in the corona that has been focused by the mirror force in the nascent solar wind (Owens et al. 2008), explaining the anti-Sunward bulk velocity of the strahl in the solar-wind rest frame. As with the ion beams, a Sunward or bi-directional electron strahl can occur when the magnetic-field configuration changes during the plasma’s passage from the Sun (Gosling et al. 1987; Owens et al. 2017). Figure 6 shows an example of an electron velocity distribution function measured in the solar wind. This distribution exhibits a significant strahl at $v_{\parallel }>0$ but shows no clear halo component. We reiterate our paradigm that all successful solar-wind acceleration and heating scenarios must account for the observed kinetic structure of the solar wind, including these features in the electron distributions. At highest energies $\gtrsim 2\,\mathrm {keV}$, a nearly isotropic *superhalo* of electrons exists; however, its number density is very small compared to the densities of the other electron species ($\lesssim \,10^{-5}\,\mathrm {cm}^{-3}$ at 1 au), and its origin remains poorly understood (Lin 1998; Wang et al. 2012; Yang et al. 2015; Tao et al. 2016).

Observations of the superthermal electrons (i.e., strahl and halo) reveal that $(n_{\mathrm {s}}+n_{\mathrm {h}})/n_{\mathrm {e}}$ remains largely constant with heliocentric distance, where $n_{\mathrm {s}}$ is the strahl density and $n_{\mathrm {h}}$ is the halo density. Conversely, $n_{\mathrm {s}}/n_{\mathrm {e}}$ decreases with distance from the Sun while $n_{\mathrm {h}}/n_{\mathrm {e}}$ increases (Maksimovic et al. 2005; Štverák et al. 2009; Graham et al. 2017). Various processes have been proposed to explain this phenomenon, most of which involve the scattering of strahl electrons into the halo (Vocks et al. 2005; Gary and Saito 2007; Pagel et al. 2007; Saito and Gary 2007; Owens et al. 2008; Anderson et al. 2012; Gurgiolo et al. 2012; Landi et al. 2012; Verscharen et al. 2019a).

Locally, electrons often show isothermal behavior (i.e., having a polytropic index of one) due to their large field-parallel mobility. Globally, their non-thermal distribution functions carry a large heat flux according to Eq. (30) into the heliosphere (Feldman et al. 1976; Scime et al. 1995). Observations of large-scale electron temperature profiles suggest that the electron heat flux, rather than local heating, dominates their temperature evolution (Pilipp et al. 1990; Štverák et al. 2015). These energetic considerations also reveal that a combination of processes regulate the heat flux of the distribution. Collisions and collective kinetic processes such as microinstabilities are the prime candidates for explaining electron heat-flux regulation (see Sects. 3.3.2, 6.1.2; Scime et al. 1994, 1999, 2001; Bale et al. 2013; Lacombe et al. 2014).

#### Open questions and problems

The major outstanding science questions in solar-wind physics require a detailed understanding of the interplay between the multi-scale nature and the observed kinetic features of the solar wind. This theme applies to the coronal and solar-wind heating problem as well as the overall energetics of the inner heliosphere. We remind ourselves that any answer to the heating problem must be consistent with multiple detailed observational constraints as we have seen in the previous sections.

The observed temperature profiles and overall particle energetics of ions and electrons are consequences of the complex interactions of global heat flux, Coulomb collisions (Sect. 3), local wave action (Sect. 4), turbulent heating (Sect. 5), microinstabilities (Sect. 6), and double-adiabatic expansion (Mihalov and Wolfe 1978; Feldman et al. 1979; Gazis and Lazarus 1982; Marsch et al. 1983, 1989; Pilipp et al. 1990; McComas et al. 1992; Gazis et al. 1994; Issautier et al. 1998; Maksimovic et al. 2000; Matteini et al. 2007; Cranmer et al. 2009; Hellinger et al. 2011; Le Chat et al. 2011; Hellinger et al. 2013; Štverák et al. 2015). We still lack a detailed physics-based understanding of the majority of these processes, and the quantification of these processes and their role for the overall energetics of the solar wind remains one of the most outstanding science problems in space research.

**Fig. 7:**
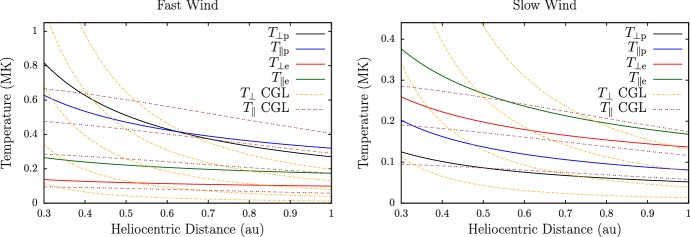
Temperature profiles in the inner heliosphere for fast (left) and slow (right) wind. We show radial power-law fits to proton-temperature measurements separated by fast ($700\,\mathrm {km/s}\le U_{\mathrm {p}r}\le 800\,\mathrm {km/s}$) and slow ($300\,\mathrm {km/s}\le U_{\mathrm {p}r}\le 400\,\mathrm {km/s}$) solar-wind conditions from Hellinger et al. (2013). Likewise, we show radial power-law fits to electron-temperature measurements separated by fast ($U_{\mathrm {p}r}\ge 600\,\mathrm {km/s}$) and slow ($U_{\mathrm {p}r}\le 500\,\mathrm {km/s}$) solar-wind conditions from Štverák et al. (2015). The thin-dashed lines indicate the CGL temperature profiles according to Eqs. (44) and (45), where we set the right-hand sides of both equations to zero and determine the magnetic field through Eqs. (16) and (17) using $n_{j}\propto 1/r^2$, $\theta =90^{\circ }$, $r_{\mathrm {eff}}=10\,R_{\odot }$, and $U_{\mathrm {p}r}=500\,\mathrm {km/s}$

Observed temperature profiles (including anisotropies) are some of the central messengers about the overall solar-wind energetics, apart from velocity profiles. Figure 7 illustrates the radial evolution of the proton and electron temperatures in the directions perpendicular and parallel to the magnetic field and separated by fast and slow wind. We also show the expected temperature profiles under the assumption that the evolution follows the double-adiabatic (CGL) expansion according to Eqs. (44) and (45) only. All of the measured temperature profiles deviate from the CGL profiles to some degree, and this trend continues at greater heliocentric distances (Cranmer et al. 2009). Explaining these deviations lies at the heart of the challenge to explain coronal and solar-wind heating and acceleration.

We intend this review to give an overview over the relevant multi-scale processes in the solar wind. In the near future, data from the Parker Solar Probe (Fox et al. 2016) and Solar Orbiter (Müller et al. 2013) spacecraft will provide us with detailed observations of the local and global properties of the solar wind at different distances from the Sun. These groundbreaking observations will help us to quantify the roles of the multi-scale processes described in this review.

Section 2 describes the methods to measure solar-wind particles and fields in situ. In Sect. 3, we discuss the effects of collisions on the multi-scale evolution of the solar wind. Section 4 introduces waves, and Sect. 5 introduces turbulence as mechanisms that affect the local and global plasma behavior. We describe the role of kinetic microinstabilities and parametric instabilities in Sect. 6. In Sect. 7, we summarize this review and consider future developments in the study of the multi-scale evolution of the solar wind.

## In-situ observations of space plasmas

Observations of space plasmas can be roughly divided into two categories: *remote* and *in-situ*. Remote observations include both measurements of the plasma’s own emissions (e.g., radio waves, visible light, and X-ray photons) as well as measurements of the effects that the plasma has on emissions from other sources (e.g., Faraday rotation and absorption lines). In this way, regions such as the chromosphere that are inaccessible to spacecraft can still be studied. Additionally, imaging instruments such as coronagraphs provide information on the global structure of space plasma. Nevertheless, due to limited spectral and angular resolution, these instruments cannot provide information on all of the small-scale processes at work within the plasma. Remote observations also only offer limited information on three-dimensional phenomena. If the observed plasma is optically thick (e.g., the photosphere in visible light), its interior cannot be probed; if it is optically thin (e.g., the corona in EUV), remote observations suffer from the effects of line-of-sight integration.

In contrast, in-situ observations provide detailed information on microkinetic processes in space plasmas. Spacecraft carry in-situ instruments into the plasma to directly detect its particles and fields and thereby to provide small-scale observations of localized phenomena. Although an in-situ instrument only detects the plasma in its immediate vicinity, statistical studies of ensembles of measurements have provided remarkable insights into how small-scale processes affect the plasma’s large-scale evolution.

This section briefly overviews both the capabilities and the limitations of instruments used to observe the solar wind in situ. Although a full treatment of the subject is beyond the scope of this review, a basic understanding of these instruments is essential for the proper scientific analysis of their measurements. Section 2.1 highlights some significant heliospheric missions. Two sections are dedicated to in-situ observations of thermal ions and electrons: Sect. 2.2 overviews the instrumentation, and Sect. 2.3 addresses the analysis of particle data. Sections 2.4 and 2.5 respectively discuss the in-situ observation of the solar wind’s magnetic and electric fields. Section 2.6 presents a short description of multi-spacecraft techniques.

**Table 2:** Select heliospheric missions: completed, active, and future

Mission	Years active${}^{\mathrm{a}}$	Radial coverage${}^{\mathrm{b}}$ (au)	Source
Luna 1, 2, and 3	1959–1959	$\approx$ 1.0${}^{\mathrm{c}}$	NSSDC; Johnson (1979)
Mariner 2	1962–1962	0.866–1.003	COHOWeb
Pioneer 6	1965–1971	0.814–0.984	COHOWeb
Pioneer 7	1966–1968	1.010–1.126	COHOWeb
Pioneer 10	1972–1995	0.99–63.04	CDAWeb (PIONEER10_COHO1HR_MERGED_MAG_PLASMA)
Pioneer 11	1973–1992	1.00–36.26	CDAWeb (PIONEER11_COHO1HR_MERGED_MAG_PLASMA)
Pioneer Venus	1978–1992	0.72–0.73	CDAWeb (PIONEERVENUS_COHO1HR_MERGED_MAG_PLASMA)
ISEE-3 (ICE)	1978–1990	0.93–1.03	CDAWeb (ISEE-3_MAG_1MIN_MAGNETIC_FIELD)
Helios 1	1974–1981	0.31–0.98	CDAWeb (HELIOS1_COHO1HR_MERGED_MAG_PLASMA)
Helios 2	1976–1980	0.29–0.98	CDAWeb (HELIOS2_COHO1HR_MERGED_MAG_PLASMA)
Ulysses	1990–2009	1.02–5.41	CDAWeb (UY_COHO1HR_MERGED_MAG_PLASMA)
Cassini	1997–2017	0.67–10.07	COHOWeb; OMNIWeb Plus (helio1day)
STEREO B	2006–2014	1.00–1.09	CDAWeb (STB_COHO1HR_MERGED_MAG_PLASMA)
Voyager 1	1977–	1.01–140.71${}^{\mathrm{d}}$	CDAWeb (VOYAGER1_COHO1HR_MERGED_MAG_PLASMA)
Voyager 2	1977–	1.00–118.91${}^{\mathrm{d}}$	CDAWeb (VOYAGER2_COHO1HR_MERGED_MAG_PLASMA)
Wind	1994–	0.972–1.017	CDAWeb (WI_OR_PRE)
SOHO	1995–	0.972–1.011	CDAWeb (SO_OR_PRE)
ACE	1997–	0.973–1.010	CDAWeb (AC_OR_SSC)
New Horizons	2006–	11.268–42.775${}^{\mathrm{d}}$	CDAWeb (NEW_HORIZONS_SWAP_VALIDSUM)
STEREO A	2006–	0.96–0.97	CDAWeb (STA_COHO1HR_MERGED_MAG_PLASMA)
DSCOVR	2015–	0.973–1.007	CDAWeb (DSCOVR_ORBIT_PRE)
PSP	2018–	0.0459–0.25${}^{\mathrm{e,f}}$	Fox et al. (2016)
Solar Orbiter	2020${}^{\mathrm{g,h}}$	0.28–1.2${}^{\mathrm{e}}$	Müller et al. (2013)
IMAP	2024${}^{\mathrm{g}}$	0.973–1.007${}^{\mathrm{i}}$	NASA Release 18-046

${}^{\mathrm{a}}$Year of launch to final year (with non-fill data) in cited dataset

${}^{\mathrm{b}}$Incomplete for some missions due to data gaps

${}^{\mathrm{c}}$Exact range not available

${}^{\mathrm{d}}$Distance still increasing; values on 2018-01-01 (Voyager 1), 2018-10-26 (Voyager 2), or 2018-10-31 (New Horizons)

${}^{\mathrm{e}}$Anticipated radial coverage

${}^{\mathrm{f}}$Perihelion of first three orbits: 0.163 au

${}^{\mathrm{g}}$Anticipated launch date

${}^{\mathrm{h}}$https://www.esa.int/Our_Activities/Space_Science/Solar_Orbiter, accessed 2019-09-10

${}^{\mathrm{i}}$Approximate radial coverage of the first Lagrangian point of the Earth–Sun system

### Overview of in-situ solar-wind missions

In-situ plasma instruments were among the first to be flown on spacecraft. Gringauz et al. (1960) used data from Luna 1, Luna 2, and Luna 3, which at the the time were known as the Cosmic Rockets, to report the first detection of super-sonic solar-wind ions as predicted by Parker (1958). These observations were soon confirmed by Neugebauer and Snyder (1962), who used in-situ measurements from Mariner 2 en route to Venus.

**Fig. 8:**
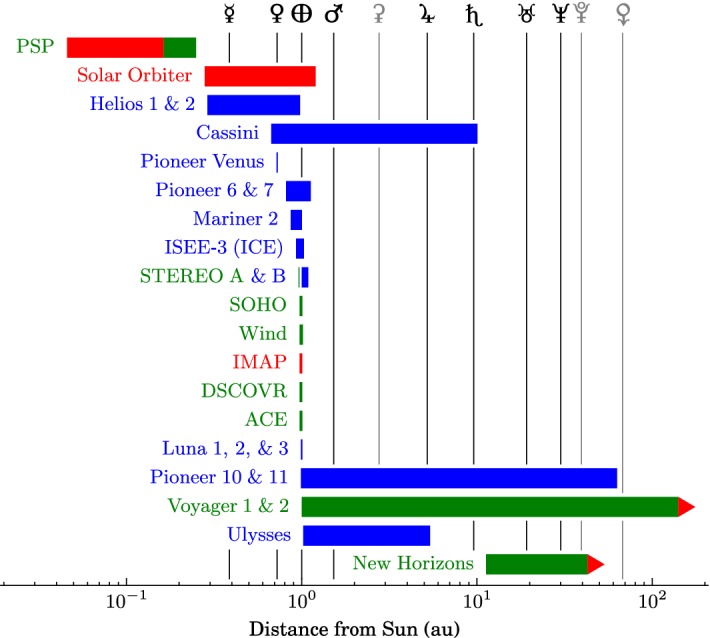
Radial coverage of select heliospheric missions based on Table 2. Colors indicate the status of each mission: completed (blue), active (green), and future (red). The colored bar for each mission does not reflect any data gaps that may be present in its dataset(s). Mixed coloring has been used for PSP to reflect that, while the mission is active, final radial coverage has not yet been achieved. Red arrows indicate that the radial coverages of Voyager 1 and 2 and New Horizons are still increasing. Vertical lines indicate the semi-major axes of the eight planets (black) and the dwarf planets Ceres, Pluto, and Eris (gray)

Since then, numerous spacecraft have carried in-situ instruments throughout the heliosphere to observe the solar wind’s particles and fields. Table 2 lists a selection of these missions grouped as completed, active, and future missions. The column “Radial Coverage” lists the ranges of heliocentric distance for which in-situ data are available, which are presented graphically in Fig. 8. Currently, Voyager 1 (Kohlhase and Penzo 1977) is the most distant spacecraft from the Sun—a superlative that it will continue to hold for the foreseeable future. Helios 2 (Porsche 1977) held for several decades the record for closest approach to the Sun, but, in late 2018, Parker Solar Probe (Fox et al. 2016) achieved a substantially closer perihelion.

### Thermal-particle instruments

Thermal particles constitute the most abundant but lowest-energy particles in solar-wind plasma. Although no formal definition exists, the term commonly refers to particles whose energies are within several (“a few”) thermal widths of the plasma’s bulk velocity. We define these as protons with energies $\lesssim \,10\, \mathrm{keV}$ and electrons with energies $\lesssim \,100\, \mathrm{eV}$ under typical solar-wind conditions at 1 au. We note, however, that most thermal-particle instruments cover a wider range of energies.

Although particle moments such as density, bulk velocity, and temperature are useful quantities for characterizing the plasma, these parameters generally cannot be measured directly. Instead, thermal-particle instruments measure *particle spectra*, which give the distribution of particle energies in various directions. These spectra must then be analyzed to derive values for the particle moments (see Sect. 2.3).

This section focuses on the basic design and operation of three types of thermal-particle instruments: *Faraday cups*, *electrostatic analyzers (ESAs)*, and *mass spectrometers*. Since particle acceleration beyond thermal energies is outside of the scope of this review, we do not address instruments for measuring higher-energy particles.

Some other techniques and instruments exist for measuring thermal particles in solar-wind plasma, but we omit extensive discussion of these since they generally provide limited information about the phase-space structure of particle distributions. For example, an electric-field instrument can be used to infer some electron properties (especially density; see Sect. 2.5). Likewise *Langmuir probes* provide some electron moments (Mott-Smith and Langmuir 1926). A series of bias voltages is applied to a Langmuir probe relative either to the spacecraft or to another Langmuir probe. The electron density and temperature can then be inferred from measurements of current at each bias voltage. The Cassini spacecraft included a spherical Langmuir probe (Gurnett et al. 2004) along with other plasma instruments (Young et al. 2004).

#### Faraday cups

Faraday cups rank among the earliest instruments for studying space plasmas. Historically noteworthy examples include the charged-particle traps on Luna 1, Luna 2, and Luna 3 (Gringauz et al. 1960) and the Solar Plasma Experiment on Mariner 2 (Neugebauer and Snyder 1962), which provided the first in-situ observations of the solar wind’s supersonic ions. Since then, Faraday cups on Pioneer 6 and Pioneer 7 (Lazarus et al. 1966, 1968), Voyager 1 and 2 (Bridge et al. 1977), Wind (Ogilvie et al. 1995), and DSCOVR (Aellig et al. 2001a) have continued to observe solar-wind particles.

**Fig. 9:**
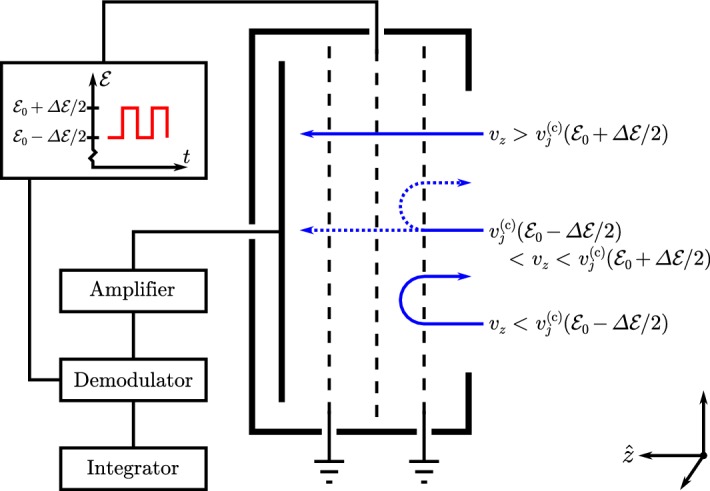
Simplified cross-sectional diagram of a Faraday cup for observing ions. The cup’s aperture is on the right, its *collector plate* is on the left, and its three grids are indicated by dashed lines. A square-wave voltage, ${\mathcal {E}} = {\mathcal {E}}_0 \pm \varDelta {\mathcal {E}}/2 > 0$, is applied to the middle grid, which is known as the *modulator*. Blue arrows indicate inflowing *j*-ions. Depending on $v_z$, the normal component of the ion’s velocity, it is either always accepted by the modulator (high speed), always rejected (low speed), or only accepted when the modulator’s voltage is low (intermediate speed). The accepted ions produce a current at the collector plate, which the detection system amplifies, demodulates, and integrates to measure, in effect, the current from only the intermediate-speed ions according to Eq. (66)

As depicted in Fig. 9, a Faraday cup consists of a grounded metal structure with an aperture. A typical Faraday cup has a somewhat “squat” geometry with a wide aperture so that it accepts incoming particles from a wide range of directions. For example, the full-width half-maximum field of view of each of the Wind/SWE Faraday cups is about $105^\circ$. At the back of the cup is a metal *collector* plate, which receives the current *I* of the inflowing charged particles.

Figure 9 shows three of the fine mess grids that are placed between a Faraday cup’s aperture and collector. The inner and outer grids are electrically grounded. A voltage ${\mathcal {E}}$ is applied to the middle grid, known as the *modulator*, to restrict the ability of particles to reach the collector. We define $\hat{\mathbf{z}}$ to indicate the direction into the Faraday cup so that $-\hat{\mathbf{z}}$ is the cup’s *look direction*. Consider a *j*-particle of mass $m_j$ and charge $q_j$ that enters the cup with a velocity $\mathbf{v}$. For a modulator voltage ${\mathcal {E}}$, the particle can only reach the collector if the normal component of its velocity, $v_z = \mathbf{v}\cdot \hat{\mathbf{z}}$, is greater than the *cutoff speed*

64 $$\begin{aligned} v^{(\mathrm {c})}_j({\mathcal {E}}) \equiv {\left\{ \begin{array}{ll} \sqrt{\displaystyle \frac{2\,q_j\,{\mathcal {E}}}{m_j}} &{} \quad \text {if}\; \; q_j\,{\mathcal {E}} > 0 \\ 0 &{} \quad \text {else} \end{array}\right. }. \end{aligned}$$

When ${\mathcal {E}}$ and $q_j$ have opposite signs, the modulator places no restriction on the particle’s ability to reach the collector.

Typically, the modulator is not kept at a constant voltage but rather alternated between two voltages:

65 $$\begin{aligned} {\mathcal {E}} = {\mathcal {E}}_0 \pm \frac{ \varDelta {\mathcal {E}}}{2}, \end{aligned}$$

where ${\mathcal {E}}_0$ is the offset and $\varDelta {\mathcal {E}}$ is the peak-to-peak amplitude. In this configuration, the detector circuit is designed to use synchronous detection to measure the difference in the collector current between the two states:

66 $$\begin{aligned} \varDelta I({\mathcal {E}}_0, \varDelta {\mathcal {E}}) = I\left( {\mathcal {E}}_0-\frac{\varDelta {\mathcal {E}}}{2}\right) - I\left( {\mathcal {E}}_0+\frac{\varDelta {\mathcal {E}}}{2}\right) . \end{aligned}$$

Essentially, $\varDelta I$ is the current from particles whose velocities are sufficient for them to reach the collector when the modulator voltage is low but not when it is high. This method suppresses contributions to the collector current that do not vary with the modulator voltage. These contributions include the signal from any particle species with a charge opposite that of the modulator since, per Eq. (64), the modulator does not restrict the inflow of such particles. This method also mitigates the effects of photoelectrons, which are liberated from the collector by solar UV photons and whose signal can exceed that of solar-wind particles by orders of magnitude (Bridge et al. 1960).

A set of ${\mathcal {E}}_0$ and $\varDelta {\mathcal {E}}$ values defines a *voltage window*. By measuring the differential current $\varDelta I$ for a series of these, a Faraday cup produces an energy distribution of solar-wind particles. The size and number of voltage windows determine the spectral resolution and range, which, for many Faraday cups, can be adjusted in flight to accommodate changing plasma conditions. Since a Faraday cup is simply measuring current, its detector electronics often exhibit little degradation with time. For example, Kasper et al. (2006) demonstrate that the absolute gain of each of the Wind/SWE Faraday cups (Ogilvie et al. 1995) drifts $\lesssim \,0.5\%$ per decade.

Various approaches exist to use Faraday cups to measure the direction of inflowing particles, which is necessary for inferring parameters such as bulk velocity and temperature anisotropy. The Voyager/PLS investigation (Bridge et al. 1977) and the BMSW solar-wind monitor on SPECTR-R (Šafránková et al. 2008) include multiple Faraday cups pointed in different directions. DSCOVR/PlasMag (Aellig et al. 2001a) has only a single Faraday cup but multiple collector plates: a *split collector*. Each collector is off-axis from the aperture and thus has a slightly different field of view. Pioneer 6, Pioneer 7 (Lazarus et al. 1966, 1968), and Wind (Ogilvie et al. 1995) are spinning spacecraft, so their Faraday cups make measurements in various directions as the spacecraft rotate.

A Faraday cup’s *response function* is a mathematical model for what the instrument measures under different plasma conditions: i.e., an expression for $\varDelta I$ as a function of the particle distribution functions. For simplicity, we initially consider only one particle species *j* and assume that the distribution function $f_j$ is, during the measurement cycle, a function of $\mathbf{v}$ only. The number density of *j*-particles in a phase-space volume $\mathrm {d}^3\mathbf{v}$ centered on $\mathbf{v}$ is

67 $$\begin{aligned} \mathrm {d}n_j = f_j(\mathbf{v})\,\mathrm {d}^3\mathbf{v}. \end{aligned}$$

The current that the Faraday cup measures from the particles in this volume is

68 $$\begin{aligned} \mathrm {d}I_j = q_jv_zA(\theta ,\phi )\,\mathrm {d}n_j = q_jv_zA(\theta ,\phi )f_j(\mathbf{v})\,\mathrm {d}^3\mathbf{v}, \end{aligned}$$

where $(v,\theta ,\phi )$ are the spherical coordinates of $\mathbf{v}$, and $A(\theta ,\phi )$ is the Faraday cup’s effective collecting area as a function of particle-inflow direction.^6^ If the modulator voltage spans the voltage window ${\mathcal {E}}_0\pm \varDelta {\mathcal {E}}/2$, then the contribution of all *j*-particles to the measured differential current is

69 $$\begin{aligned} \varDelta I_j = \int \mathrm {d}I_j = q_j \int \limits _{v^{(\mathrm {c})}_j({\mathcal {E}}_0-\varDelta {\mathcal {E}}/2)}^{v^{(\mathrm {c})}_j({\mathcal {E}}_0+\varDelta {\mathcal {E}}/2)} \mathrm {d}v_z \, v_z \int \limits _{-\infty }^{\infty } \mathrm {d}v_y \int \limits _{-\infty }^{\infty } \mathrm {d}v_x \, A(\theta ,\phi ) f_j(\mathbf{v}). \end{aligned}$$

Since a Faraday cup cannot distinguish current from different types of particles, the measured current is

70 $$\begin{aligned} \varDelta I = \sum _j \varDelta I_j, \end{aligned}$$

where the sum is carried out over all particle species in the plasma.

Equations (69) and (70) provide the general form of the response function of a Faraday cup. Section 2.3 overviews the process of inverting the response function to determine the particle moments from a measured particle spectrum.

**Fig. 10:**
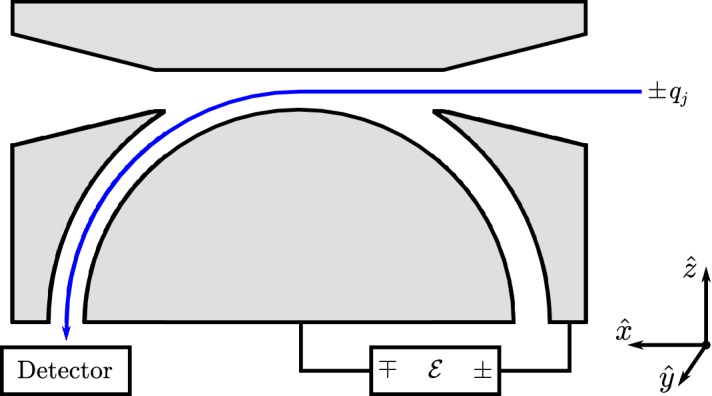
Simplified cross-sectional diagram of a *top-hat* style electrostatic analyzer (ESA). The aperture is shown on the upper left and right, and can provide up to $360^\circ$ of coverage of azimuth $\phi$. In contrast, only particles within a limited range of elevation $\theta$ are able to pass through the curved *collimator* plates and reach the *detector*. A DC voltage ${\mathcal {E}}$ is sustained between the plates and sets the sign and value of the target *energy per charge* $K/q_j$ for incoming particles. The spacing between the collimator plates defines the width of the energy windows

#### Electrostatic analyzers

Like Faraday cups, electrostatic analyzers (ESAs) have a long history of use in the observation of thermal particles in the solar wind. Though ESAs are substantially more complex than Faraday cups, they enable much more direct and detailed studies of distribution functions (see Sect. 2.3.1). Additionally, they can be combined with mass spectrometers (see Sect. 2.2.3) to directly probe the ion composition of the plasma.

Figure 10 shows a simplified cross-section of the common *top-hat* design for an ESA (Carlson et al. 1983). Such a device consists of two hemispherical shells that are nested concentrically so as to leave a narrow gap between them. Particles enter via a hole in the top of the larger hemisphere and are then subjected to the electric field that is created by maintaining a DC voltage ${\mathcal {E}}$ between the two hemispheres. The value of ${\mathcal {E}}$ and the curvature and spacing of the hemispheres define an *energy-per-charge* range for an incoming particle to reach the detectors at the base of the hemispheres. If an incoming particle has a kinetic energy *K* and charge $q_j$, it can only reach the detectors if the ratio $K/q_j$ falls within that range. To generate a particle spectrum, ${\mathcal {E}}$ is swept through a series of values. The range of particle energies is set by the range of ${\mathcal {E}}$ values, which, on most ESAs, can be adjusted in flight. Nevertheless, the width of an ESA’s energy window $\varDelta K/K_0$ is fixed geometrically by the spacing between its collimator plates. In contrast, the width of a Faraday cups’ energy window is adjustable in flight since it is set by a voltage range according to Eq. (65).

An ESA’s detectors are typically arranged around the base of the hemispheres. While Faraday cups detect incoming particles by measuring their net current, an ESA’s detectors usually count particle cascades generated by the strikes from individual particles. Such detectors would be impractical for a Faraday cup because they would be overwhelmed by solar UV photons. On a top-hat ESA, the tight spacing of the deflectors and a low-albedo coating^7^ on their surfaces ensure that very few photons reach the detectors. Each of the detectors is typically some type of electron multiplier, which uses an electrostatic potential in such a way that a strike by a single charged particle produces a cascade of electrons, which can then be registered. *Channel electron multipliers (CEMs)* were used for ACE/SWEPAM (McComas et al. 1998a), while *micro-channel plates (MCPs)* were used for Wind/3DP (Lin et al. 1995) and STEREO/IMPACT/SWEA (Sauvaud et al. 2008). Both CEM and MCP detectors require more complex calibration than is needed for a Faraday cup. For example, after each particle strike, an electron multiplier experiences a *dead time*, during which the electron cascade is in progress and the detector cannot respond to another particle. Furthermore, electron multipliers (and MCPs in particular) often exhibit significant degradation in their efficiency with time.

A typical top-hat ESA has a *fan-beam* field of view. The size and number of detectors define its azimuthal resolution and coverage, and ESAs can be designed with up to $360^\circ$ of $\phi$-coverage. In contrast, most ESAs only sample particles over a limited range of elevation $\theta$, and a number of strategies have been employed to provide $\theta$-coverage. The ESAs in the Helios plasma investigation (Schwenn et al. 1975; Rosenbauer et al. 1977) and in Wind/3DP (Lin et al. 1995) were designed to rely on spacecraft spin to sweep their fan beams. Although the Cassini spacecraft was three-axis stabilized, its CAPS instrument suite was mounted on an actuator, which a motor rotated through about $180^\circ$ of azimuth every 3 min (Young et al. 2004). The MAVEN spacecraft is likewise three-axis stabilized, but its SWIA instrument (Halekas et al. 2015) incorporated a second set of electrostatic deflectors to effectively steer its fan beam by adjusting the path of ions entering the top hat. Finally, the unique design of MESSENGER/FIPS (Andrews et al. 2007) moved beyond the top hat to give that instrument wide $\theta$-coverage (versus a fan beam) but reduced aperture size.

For any given value of ${\mathcal {E}}$, each ESA detector essentially has its own effective collecting area $A_j(K,\theta ,\phi )$, which depends on the energy $K = m_jv^2/2$ and direction $(\theta ,\phi )$ of incoming *j*-particles. The number of *j*-particles detected from an infinitesimal volume $\mathrm {d}^3\mathbf{v}$ of phase-space during a time interval $\varDelta t$ is

71 $$\begin{aligned} \mathrm {d}N_j = \varDelta t\, v A_j(K,\theta ,\phi )\, \mathrm {d}n_j, \end{aligned}$$

where $\mathrm {d}n_j$ is the number density of *j*-particles in $\mathrm {d}^3\mathbf{v}$. Substituting Eq. (67) and converting to spherical coordinates gives

72 $$\begin{aligned} \mathrm {d}N_j = \frac{2\,\varDelta t}{m_j^2} A_j(K,\theta ,\phi ) f_j(K,\theta ,\phi ) K \sin \theta \, \mathrm {d}K\, \mathrm {d}\theta \, \mathrm {d}\phi , \end{aligned}$$

where $f_j$ has been parameterized in energy and direction rather than vector velocity. The total number of *j*-particles detected in $\varDelta t$ is

73 $$\begin{aligned} \varDelta N_j \!= \!\int \mathrm {d}N_j\!=\! \frac{2\,\varDelta t}{m_j^2} \int \limits _0^{\infty } \mathrm {d}K\, K \int \limits _{0}^{\pi } \mathrm {d}\theta \, \sin \theta \int \limits _0^{2\pi } \mathrm {d}\phi \, A_j(K,\theta ,\phi ) f_j(K,\theta ,\phi ).\qquad \end{aligned}$$

Formally, the integrals in Eq. (73) are carried out over all energies and directions (i.e., all of phase space) but most ESAs are designed so that a given detector is only sensitive to particles from a relatively narrow range of energies and directions. Consequently, the detector’s effective collecting area is often approximated as

74 $$\begin{aligned} A_j(K,\theta ,\phi ) \approx {\left\{ \begin{array}{ll}\displaystyle \frac{A_0}{\sin \theta _0} &{} \quad \text {if}\;\; |K-K_0|<\varDelta K,\, |\theta -\theta _0|<\varDelta \theta , \, |\phi -\phi _0| <\varDelta \phi \\ 0 &{}\quad \text {else} \end{array}\right. },\nonumber \\ \end{aligned}$$

where $A_0$ is the nominal collecting area, $(\theta _0,\phi _0)$ is the look direction, $\varDelta \theta$ and $\varDelta \phi$ set the field of view, and $K_0$ and $\varDelta K$ set the energy range of *j*-particles. Using Eq. (74) and assuming that $\varDelta K$, $\varDelta \theta$, and $\varDelta \phi$ are small relative to variations in $f_j(K,\theta ,\phi )$, we approximate Eq. (73) as

75 $$\begin{aligned} \varDelta N_j \approx \frac{2A_0K_0}{m_j^2} \varDelta t\, \varDelta K\, \varDelta \theta \, \varDelta \phi \, f_j(K_0,\theta _0,\phi _0)\approx \frac{2K_0^2}{m_j^2} G f_j(K_0,\theta _0,\phi _0), \end{aligned}$$

where

76 $$\begin{aligned} G \equiv A_0\, \varDelta t \frac{\varDelta K}{K_0} \varDelta \theta \, \varDelta \phi \end{aligned}$$

is known as the *geometric factor*. ESAs are often designed and operated in such a way that *G* is approximately constant.

If an ESA does not have any mass-spectrometry capability (see Sect. 2.2.3), then each of its detectors measures the count of all particles of any species that reach it. Thus, the measured quantity is

77 $$\begin{aligned} \varDelta N = \sum _j \varDelta N_j, \end{aligned}$$

where the sum is carried out over all particle species *j*.

Equations (73) and (77) specify the response function of a top-hat ESA. A particle spectrum from such an instrument consists of a set of measured $\varDelta N$-values made over various ${\mathcal {E}}$-values and in various directions. Section 2.3 describes how the response function can be used to extract information about particle distribution functions from a measured spectrum.

#### Mass spectrometers

As noted above, neither a Faraday cup nor an ESA can, on its own, directly distinguish among different ion species: they simply measure the current and counts, respectively, of the incoming particles. A limited composition analysis, though, is still possible because the voltage ${\mathcal {E}}$ needed for either type of instrument to detect a *j*-particle of speed *v* is proportional to $m_j/q_j$. Though relative drift is often observed among different particle species in the solar wind, it generally remains far less than the bulk speed (see Sect. 1.4.4). Thus, in a particle spectrum, the signals from different particle species appear shifted by their mass-to-charge ratios. By separately analyzing these signals (see Sect. 2.3), values can be inferred for the moments of the various particle species.

This strategy does have significant limitations. First, it provides no mechanism for distinguishing ions with the same mass-to-charge ratio (e.g., ${}^{12}\mathrm{C}^{3+}$ and ${}^{16}\mathrm{O}^{4+}$). Second, even when particle species have distinct mass-to-charge ratios, ambiguity can still arise from the overlap of their spectral signal. For example, the mass-to-charge ratios of protons and $\alpha$-particles differ enough that values for their moments can often be derived for both species from Faraday-cup (e.g., Kasper 2002, Chapter 4) and ESA (e.g., Marsch et al. 1982b) spectra. Nevertheless, the $\alpha$-particle signal can suffer confusion with minor ions (e.g., Bame et al. 1975), and, especially at low Mach numbers, the proton and $\alpha$-particle signals can almost completely overlap (e.g., Maruca 2012, Sect. 3.3).

A mass spectrometer is required to achieve the most accurate measurements of solar-wind composition (see also the more complete review by Gloeckler 1990). As opposed to being a separate instrument, a mass spectrometer is typically incorporated into an ESA as its detector system and is used to measure the speed of each particle. The ESA ensures that only particles within a known, narrow range of energy per charge pass through. As each particle enters the mass spectrometer, an electric field accelerates it by a known amount. The particle then triggers a start signal by liberating electrons from a thin foil,^8^ which are detected via an MCP. Next, the particle travels a known distance $\varDelta s$ to another foil.^9^ The particle triggers a stop signal by passing through this latter foil before finally reaching the detector. The time $\varDelta t$ between the start and stop signals is the particle’s *time of flight*, a measurement of which allows the particle’s speed $v=\varDelta s/\varDelta t$ through the mass spectrometer to be inferred.

Several different designs have been developed for mass spectrometers for heliophysics. In a time-of-flight versus energy (TOF/E) mass spectrometer, such as Ulysses/SWICS (Gloeckler et al. 1992), ACE/SWICS (Gloeckler et al. 1998, Sect. 3.1), and STEREO/IMPACT/PLASTIC (Galvin et al. 2008), *solid-state detectors (SSDs)* are used to ultimately detect each ion. Unlike an electron multiplier, an SSD is able to measure the energy of individual charged particles. Therefore, a TOF/E instrument measures each ion’s initial energy per charge, speed through the instrument, and residual energy at the detector. Together, these quantities provide sufficient information to determine the ion’s mass, charge, and initial speed. In contrast, a high-mass-resolution spectrometer (HMRS) such as ACE/SWIMS (Gloeckler et al. 1998, Sect. 3.2) does not need to measure the ions’ residual energy and can simply use MCP detectors. An HMRS exploits the fact that passing through the start foil tends to decrease an ion’s charge state to either 0 or ${+}\,1$. The particle then passes through a known but non-uniform electric field, which deflects the singly ionized particle to the detectors. The electric field causes the time of flight to be mass dependent, so each particle’s mass can be inferred.

### Analyzing thermal-particle measurements

A particle spectrum, whether measured by a Faraday cup or an ESA, must be processed in order to extract information about the observed particles. This involves inverting the instrument’s response function—Eqs. (69) and (70) for a Faraday cup, and Eqs. (73) and (77) for an ESA—so that particle moments or phase-space densities can be derived from measured current or counts. This section briefly describes three methods for achieving this: *distribution-function imaging*, *moments analysis*, and *fitting of model distribution functions*.

#### Distribution-function imaging

Equation (75) suggests a very simple method for interpreting a particle spectrum from an ESA. The number of counts $\varDelta N_j$ of *j*-particles is approximately proportional to the value of the *j*-particles’ distribution function $f_j$ at some point in phase space. If only *j*-particles are considered, then the set of measured $\varDelta N$-values (i.e., the particle spectrum) can be used to give a set of values for $f_j$ across phase space. In this sense, an ESA’s particle spectrum can be thought of as an image of a distribution function. This is the method employed by Marsch et al. (1982a, b) in their well-known contour-plots of proton and $\alpha$-particle distribution functions from the Helios mission (see also Figs. 5, 6 of this review). Since this technique is not focused on extracting the values of particle moments, it is especially well suited to studying the three-dimensional structure of distribution functions and non-Maxwellian features.

Nevertheless, distribution-function imaging carries significant limitations. First, in the case of ion measurements, significant confusion can arise among the various ion species in the plasma (see Sect. 2.2.3). If an ESA does not have a mass spectrometer, it simply measures the total count of particles $\varDelta N$ rather than each individual $\varDelta N_j$. Second, various assumptions are made in deriving Eq. (75). Notably, the field of view and energy range were taken to be small relative to the scale of variations in the distribution function. When these assumptions break down, this technique returns a distorted image of $f_j$. Third, this technique cannot be applied to observations from a Faraday cup. Essentially, a Faraday cup’s large field of view means that each of its $\varDelta I$-measurements samples a large region of phase space. The integrals in Eq. (69) cannot be easily simplified to give an expression like Eq. (75).

Though ESA images of distribution functions can provide tremendous insight into phase-space structure, care must be exercised to properly account for instrumental effects. Any ESA has finite angular and energy resolutions, which must be considered when interpreting their output. An irregularity in a distribution function may seem significant in a contour plot but actually result from only a single datum with a low number of particle counts. Such finite-resolution effects are often more pronounced in proton versus electron data because protons, being supersonic, are concentrated into a narrow beam of phase space. A related effect arises in both ion and electron data from the finite period of time required for an ESA to sweep through its angular and energy ranges. Especially during periods of high variability in the solar wind, this may result in distribution-function images that constitute “hybrids” of distinct plasma conditions.

#### Moments analysis

Moments analysis provides the most direct method for estimating particle moments from a measured particle spectrum. Essentially, this technique relies on deriving relationships between the moments of a distribution function (see Sect. 1.4.1) and the moments of the measured quantity: $\varDelta I_j$ for a Faraday cup or $\varDelta N_j$ for an ESA. For the latter case, Eq. (75) shows that $\varDelta N_j$ is approximately proportional to $f_j$. Thus, each moment of $f_j$ can be approximated with a discrete integral of $\varDelta N_j$: a sum over all the measured $\varDelta N$-values. For a Faraday cup, the relationship between $\varDelta I_j$ and $f_j$ in Eq. (69) is more complex, but similar expressions exist to relate the moments of $f_j$ to sums of the measured $\varDelta I$-values (see, e.g., Kasper et al. 2006, Appendix A). In either case, the calculations are relatively simple. For this reason, moments analyses are commonly implemented in spacecraft flight computers, which often have limited computational resources or limited down-link bandwidth for the transmission of full particle spectra.

Moments analysis carries the significant limitation that it provides no mechanism for easily distinguishing different components of a distribution function (e.g., its core and beam), or, in the case of ions, for differentiating among species (see Sect. 2.2.3). Additionally, the particle spectrum must provide excellent coverage of $f_j$ in phase space so that the discrete integrals of the measured $\varDelta I$- or $\varDelta N$-values can reasonably approximate the infinite integrals of $f_j$ that define its moments.

#### Fitting model distribution functions

In a fitting analysis of a particle spectrum, a *model distribution* (such as those defined in Sect. 1.4.3) is chosen for each $f_j$-component and particle species under consideration. These model distributions are then substituted into the expression for $\varDelta I$ for a Faraday cup in Eq. (70) or $\varDelta N$ for an ESA in Eq. (77). This substitution gives an expression for the measured quantity, $\varDelta I$ or $\varDelta N$, in terms of the fit parameters of the model distributions: e.g., particle densities, velocities, and temperatures. This model can then be fit to a measured spectrum to derive estimates of the particle moments.

Unlike moments analysis, fitting allows for the direct treatment of multiple $f_j$-components or ion species. It also allows data to be weighted based on the uncertainty in each measurement and does not require that the particle spectrum cover almost all of phase space. Indeed, Kasper et al. (2006) use the microkinetic limits on temperature anisotropy to infer that fitting model distribution functions to ion measurements from the Wind/SWE Faraday cups produces temperature values that are significantly more accurate than those returned from a moments analysis.

The greatest disadvantage of fitting is the need to assume a model distribution. If such a model does not capture all of the features of the actual distribution function, the fitting results are unreliable. In addition, the complexity of the functions involved usually necessitates the use of non-linear fitting algorithms (e.g., the Levenberg–Marquardt algorithm; see Marquardt 1963), which are computationally intensive and generally cannot be implemented on spacecraft computers.

### Magnetometers

This section provides a brief overview of the three types of magnetometers most commonly used on heliophysics missions: *search-coil magnetometers*, *fluxgate magnetometers*, and *helium magnetometers*. The reviews by Ness (1970), Acuña (1974, 2002), and Smith and Sonett (1976) provide much more detailed treatments of these and other types of magnetometers.

#### Search-coil magnetometers

Though simpler in design than fluxgate and helium magnetometers, search-coil magnetometers have been less frequently flown on space-physics missions because of their poor sensitivity to background magnetic fields and low-frequency magnetic fluctuations. The search-coil magnetometer was first used in space on Pioneer 1 (Sonett et al. 1960). Later, search coils were included in Wind/Waves (Bougeret et al. 1995), Cluster/STAFF (Cornilleau-Wehrlin et al. 1997), and Themis/SCM (Roux et al. 2008).

Essentially, a search-coil magnetometer is a coil of wire that wraps around a portion of a core made from a high-permeability material, which serves to amplify the magnetic field. Let $\mathbf{B}_{\mathrm{ext}}$ denote the magnetic field external to the core, which is to be measured. The magnetic field inside the core is

78 $$\begin{aligned} \mathbf{B}_{\mathrm{int}} = \mu _{\mathrm {c}} \mathbf{B}_{\mathrm{ext}}, \end{aligned}$$

where $\mu _{\mathrm {c}}$ is the effective relative permeability of the core. One complication is that $\mu _{\mathrm {c}}$ differs from $\mu _{\mathrm {r}}$, the relative permeability of the bulk material comprising the core. In general,

79 $$\begin{aligned} \mu _{\mathrm {c}} = \frac{\mu _{\mathrm {r}}}{1+N_{\mathrm {d}}\left( \mu _{\mathrm {r}}-1\right) }, \end{aligned}$$

where $N_{\mathrm {d}}$ is the *demagnetization factor*, which reflects the core’s particular geometry (see, e.g., Tumanski 2011, Sect. 2.4.3). For materials with relatively low permeability, $\mu _{\mathrm {c}} \approx \mu _{\mathrm {r}}$, but materials with high $\mu _{\mathrm {r}}$ are usually favored for search coils as they substantially boost sensitivity.

If the coil has ${\mathcal {N}}$ turns, then, by Faraday’s law according to Eq. (23), the voltage induced in the coil is

80 $$\begin{aligned} {\mathcal {E}} = - \frac{{\mathcal {N}} A \mu _{\mathrm {c}}}{c} \, \frac{\mathrm {d}B_{\mathrm{ext},z}}{\mathrm {d}t}, \end{aligned}$$

where *A* is the core’s cross-sectional area, and the core is oriented along the *z*-axis. Thus, a measurement of ${\mathcal {E}}$ gives the rate of change in the axial component of $\mathbf{B}_{\mathrm{ext}}$. If $B_{\mathrm{ext},z}(t)$ is sinusoidal,

81 $$\begin{aligned} B_{\mathrm{ext},z}(t) = B_{0,z} \cos \left( 2 \pi \nu t + \phi \right) , \end{aligned}$$

the coil voltage is

82 $$\begin{aligned} {\mathcal {E}}(t) = \frac{2 \pi \nu {\mathcal {N}} A \mu _{\mathrm {c}} B_{0,z}}{c} \sin \left( 2 \pi \nu t + \phi \right) . \end{aligned}$$

A single coil can only detect fluctuations in the $\mathbf{B}_{\mathrm{ext}}$ component parallel to the coil’s axis. Thus, search-coil magnetometers often include three orthogonal coils to enable measurements of the vector magnetic field.

The factor of $\nu$ in Eq. (82) indicates that a search coil’s sensitivity scales linearly with frequency. Search-coil magnetometers are thus mostly used in the frequency range from a few Hz to several kHz. A non-accelerating search coil is completely insensitive to the background magnetic field. However, a search-coil magnetometer on a spinning spacecraft can still measure a constant field since the field is non-constant in the instrument’s frame of reference. This method was employed on Pioneer 1 to make the first measurements of the interplanetary magnetic field (Sonett et al. 1960; Rosenthal 1982).

#### Fluxgate magnetometers

The fluxgate magnetometer was first invented for terrestrial use by Aschenbrenner and Goubau (1936), and since then, it has become the most widely used type of magnetometer in heliophysics missions. Although the fluxgate magnetometer is more complex than the search-coil magnetometer, it is much better suited to measuring the background magnetic field and low-frequency ($\lesssim \,10\,\mathrm {Hz}$) magnetic fluctuations.

**Fig. 11:**
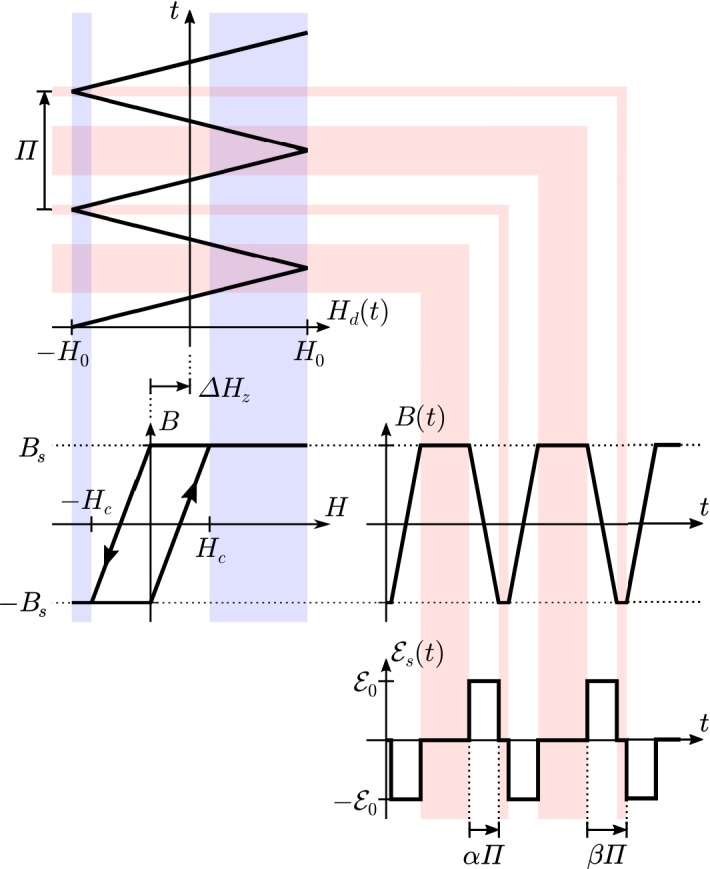
The performance of an idealized, basic fluxgate magnetometer. The hysteresis plot of the fluxgate’s ferromagnetic core is shown in the center left and indicates the magnetic field *B* in the core as a function of the auxiliary field *H* applied to it. The value of *H* is the sum of the auxiliary field $H_{\mathrm {d}}$ from the fluxgate magnetometer’s drive coil and the auxiliary field $\varDelta H_z$ associated with the magnetic field external to the instrument. The upper-left plot shows $H_{\mathrm {d}}(t)$, and $\varDelta H_z$ is represented as a horizontal shift between the two left plots. The value of $\varDelta H$ has been greatly exaggerated for illustrative purposes. The *H*-values for which the core is saturated are indicated by light-blue shading, and the times *t* when this occurs are indicated by light-red shading. The center-right plot shows the core’s magnetic field *B*(*t*), which is limited by the saturation value $B_{\mathrm {s}}$. The lower-right plot shows the voltage ${\mathcal {E}}_{\mathrm {s}}(t)$ that *B*(*t*) induces in the fluxgate magnetometer’s sense coil. After Ness (1970)

A fluxgate magnetometer relies on the *hysteresis* of ferromagnetic materials. The center-left plot in Fig. 11 shows an idealized representation of the hysteresis curve for such a material. The magnetic field $\mathbf{B}$ inside the material depends not only on the auxiliary field^10^ $\mathbf{H}$ applied to it but also on the history of the core’s magnetization. Nevertheless, there exists a critical *H*-value, $H_{\mathrm {c}}$, such that the magnetic field is saturated at a strength $B_{\mathrm {s}}$ if $\left|\mathbf{H} \right|\ge H_{\mathrm {c}}$.

In a typical design, a fluxgate magnetometer consists of a ferromagnetic core wrapped by two coils of wire: a *drive coil* and a *sense coil*. A triangle-wave current is applied to the drive coil to produce an auxiliary field $H_{\mathrm {d}}(t)$ that has an amplitude $H_0$ and period $\varPi$ (upper-left plot in Fig. 11). The core’s total auxiliary field is then

83 $$\begin{aligned} H(t) = H_{\mathrm {d}}(t) + \varDelta H_z, \end{aligned}$$

where the *z*-direction corresponds to the axis of the core, and $\varDelta H_z$ represents the contribution of the external magnetic field, which is to be measured. The value of $H_0$ is chosen to be large enough that the core experiences both positive and negative saturation during each cycle of $H_{\mathrm {d}}(t)$. As a result, the core’s magnetic field *B*(*t*) has the form of a truncated triangle wave (center-right plot in Fig. 11). A non-zero value of $\varDelta H_z$ produces a DC offset in *B*(*t*), which means that the core spends different amounts of time in positive and negative saturation. By Faraday’s law according to Eq. (23), the voltage induced in the fluxgate magnetometer’s sense coil is

84 $$\begin{aligned} {\mathcal {E}}_{\mathrm {s}} = - \frac{{\mathcal {N}}_{\mathrm {s}} A}{c} \frac{\mathrm {d}B}{\mathrm {d}t}, \end{aligned}$$

where ${\mathcal {N}}_{\mathrm {s}}$ is the number of turns in the sense coil, and *A* is the core’s cross-sectional area. Because of the offset and truncation in *B*(*t*), ${\mathcal {E}}_{\mathrm {s}}(t)$ has the form of an irregular square wave (lower-right plot in Fig. 11). We denote the duration of a positive or negative pulse as $\alpha \varPi$ and the time from the start of a positive pulse to the start of the next negative pulse as $\beta \varPi$. Then,

85 $$\begin{aligned} \alpha = \frac{H_{\mathrm {c}}}{4 H_0} \end{aligned}$$

and

86 $$\begin{aligned} \beta = \frac{1}{2}\left( 1 - \frac{\varDelta H}{H_0}\right) . \end{aligned}$$

Typically, the value of $H_0$ is chosen so that it is substantially greater than $\varDelta H_z$ and $H_{\mathrm {c}}$, in which case both $\alpha$ and $\beta$ are much less than one. The sense-coil voltage shown in Fig. 11 (lower right) has the Fourier series expansion (Ness 1970)

87 $$\begin{aligned} {\mathcal {E}}_{\mathrm {s}}(t) = {\mathcal {E}}_0 \sum _{k=1}^\infty \left( 1 - e^{-i 2 \pi \beta k} \right) \frac{\sin \left( \pi \alpha k\right) }{\pi k} \cos \left( \frac{2 \pi k t}{\varPi } \right) , \end{aligned}$$

where

88 $$\begin{aligned} {\mathcal {E}}_0 = - \frac{2 {\mathcal {N}}_{\mathrm {s}} A B_{\mathrm {s}}}{c \alpha \varPi }. \end{aligned}$$

In the absence of an external magnetic field, the values of $\varDelta H$ and $\beta$ would both be zero, which would cause all even harmonics in the above series to vanish. Thus, the second harmonic is typically measured in order to infer the value of $\varDelta H_z$ and thereby the value of $B_z$.

A single fluxgate sensor, like a single search-coil, is only sensitive to one component of the magnetic field. Consequently, fluxgate magnetometers often consist of three orthogonal sensors so that the vector magnetic field can be measured.

A fluxgate magnetometer can be used to measure the background magnetic field and low-frequency magnetic fluctuations up to a few 10’s of Hz (Ness 1970) but it has poor sensitivity to fluctuations around or above the frequency of its drive coil. Consequently, some missions carry not only fluxgate magnetometers but also search-coil magnetometers, which are better suited to measuring high-frequency magnetic fluctuations. For example, the Wind spacecraft includes both the MFI fluxgate magnetometers (Lepping et al. 1995) and the Waves search-coil magnetometers (Bougeret et al. 1995). Likewise, the four Cluster spacecraft include the FGM fluxgate magnetometers (Balogh et al. 1997) and the STAFF search-coil magnetometers (Cornilleau-Wehrlin et al. 1997).

More sophisticated designs for fluxgate magnetometers, which include additional coils and more complex geometries for the core, have been developed to improve sensitivity and to allow the instrument to be operated at higher frequencies. Notably, Geyger (1962) introduced the use of toroidal cores, which were used, e.g., for the Pioneer 11 magnetometer (Acuña 1974), Voyager/MAG (Behannon et al. 1977), Wind/MFI (Lepping et al. 1995), and STEREO/IMPACT/MAG (Acuña et al. 2008).

#### Helium magnetometers

Helium magnetometers belong to a large class of magnetometers known as *optically pumped magnetometers* (Ness 1970; Acuña 2002). Though some optically pumped magnetometers use the vapor of an alkali metal (e.g., sodium, cesium, or rubidium) as their sensing medium, helium has been more widely used in space instruments.

The sensing element of a helium magnetometer is a *cell* containing helium gas (Slocum and Reilly 1963). A radio-frequency oscillator is used to energize electrons in the gas, which collisionally excite helium atoms from their ground state, $1 ^{1}\mathrm{S}_0$, to their first excited state, $2 ^{3}\mathrm{S}_1$. Since $1 ^{1}\mathrm{S}_0$ is a singlet state, and $2 ^{3}\mathrm{S}_1$ is a triplet, the transition between them via photon emission/absorption is doubly forbidden under classical selection rules. As a result, the $2 ^{3}\mathrm{S}_1$ state is metastable.

Although collisional excitation produces equal populations for the three $2 ^{3}\mathrm{S}_1$ sub-levels, *optical pumping* produces unequal populations for this triplet (Colegrove and Franken 1960). A helium lamp serves a source of 1083 nm photons. This light is then columnated into a beam, which passes through a circularly polarized filter before reaching the cell. The 1083 nm wavelength corresponds to a helium atom’s transition between the $2 ^{3}\mathrm{S}_1$ triplet state and the three closely-spaced $2 ^{3}\mathrm{P}$ states: $2 ^{3}\mathrm{P}_0$, $2 ^{3}\mathrm{P}_1$, $2 ^{3}\mathrm{P}_2$. A helium atom in the $2 ^{3}\mathrm{S}_1$ state can transition to a $2 ^{3}\mathrm{P}$ state by absorbing one of these photons, after which it returns to $2 ^{3}\mathrm{S}_1$ via remission. However, since the photons are circularly polarized, the atom, in the presence of a magnetic field, will preferentially return to one of the $2 ^{3}\mathrm{S}_1$ sub-levels over the other two.

An infrared detector is used to measure how much of the helium lamp’s light is able to pass through the cell. The transparency of helium to 1083 nm photons depends directly on the pumping efficiency, which in turn varies with the strength of the magnetic field and the field’s angle with respect to the beam path. Thus, the magnetic field can be inferred from measurements of the intensity of transmitted light.

A vector helium magnetometer typically includes three orthogonal pairs of *Helmholtz coils* so that an arbitrary magnetic field can be applied to the cell in addition to the external magnetic field that is to be measured. In the usual operating mode, a constant-magnitude magnetic field is rotated relative to the beam path at a frequency of a few 100’s of Hz. This results in a periodic variation in the intensity of transmitted light. For a full vector measurement of the external magnetic field, the applied magnetic field is rotated through two orthogonal planes, each of which has an axis parallel to the beam path.

Vector helium magnetometers have been used on some heliophysics missions but not as many as fluxgate magnetometers. In general, helium magnetometers are more complex and often require more mass and power than fluxgate magnetometers (Acuña 2002). Nevertheless, helium magnetometers are effective for measuring strong magnetic fields, which makes them useful for planetary missions such as Pioneers 10 and 11 (Smith et al. 1975). ISEE-3 (later renamed ICE; Frandsen et al. 1978) also carried a vector helium magnetometer. Some missions, including Ulysses (Balogh et al. 1992) and Cassini (Dunlop et al. 1999; Dougherty et al. 2004), carried both vector helium and fluxgate magnetometers. The helium magnetometer on Cassini was unique in that it could be operated in either a scalar or vector mode (i.e., measure either *B* or $\mathbf{B}$). This design was developed to improve measurements of Saturn’s strong magnetic field.

### Electric-field measurements

Measurements of the vector electric field $\mathbf{E}$ in the solar wind are typically made over a very wide range of frequencies from a few kHz to tens of MHz. The most common probes of $\mathbf{E}$ are monopole and dipole antennas, the lengths of which can vary based on scientific goals and practicalities. For example, the length (spacecraft to tip) of each STEREO/Waves antenna is $6\,\mathrm{m}$ (Bale et al. 2008; Bougeret et al. 2008), while Wind/Waves has antennas that are $7.5\,\mathrm{m}$ and $50\,\mathrm{m}$ long (Bougeret et al. 1995).

Electric-field instruments for heliophysics missions often utilize multiple receivers. This not only helps to accommodate the wide range of frequencies but also allows for different observation modes to be implemented. The simplest mode is *waveform capture*, in which a time series of voltage measurements from each antenna is recorded. This mode preserves the most information about $\mathbf{E}(t)$ but produces large amounts of data and thus is generally used only as a *burst mode*. An alternative mode is *spectrum capture*, in which only the power spectral density is recorded at a predetermined set of frequencies. This significantly lowers the data volume while preserving frequency information. As a matter of practice, this mode is often implemented with a narrow-band receiver that is stepped through a series of discrete frequency ranges to measure the total power in each.

Electric-field instruments also have uses beyond simply measuring $\mathbf{E}$ for its own sake. Although these applications are beyond the scope of this review, two merit brief mention here. The first is the measurement of the *quasi-thermal noise spectrum*, which can be used to infer the properties of electrons (Meyer-Vernet and Perche 1989). When an antenna is surrounded by a plasma, the antenna’s frequency response is altered in a predictable way at frequencies near the electron plasma frequency $\omega _{\mathrm{pe}}$. As shown in Eq. (7), $\omega _{\mathrm{pe}}$ is proportional to $\sqrt{n_{\mathrm{e}}}$, so the determination of $\omega _{\mathrm{pe}}$ from the quasi-thermal noise spectrum is a direct measure of the electron density $n_{\mathrm{e}}$. In addition, the temperature and some non-thermal properties of electrons can be extracted from the shape of the quasi-thermal noise spectrum. Second, antennas can be used very effectively as dust detectors because of the large size of the antennas and the distinctive electrical signal produced by a dust grain striking an antenna (Couturier et al. 1981; Le Chat et al. 2009). The abundance and size-distribution of dust particles have been studied using measurements from STEREO/Waves (Zaslavsky et al. 2012) and Wind/Waves (Kellogg et al. 2016).

### Multi-spacecraft techniques

Most of the observational results presented in this review are based on measurements from individual spacecraft. Nevertheless, powerful techniques have been developed to analyze simultaneous in-situ measurements from multiple spacecraft to distinguish between spatial and temporal fluctuations in the plasma. This section offers a brief description of the key concepts.

Spacecraft separated by relatively large distances ($\gtrsim 0.1\,\mathrm{au}$) offer particular benefits for observing remote or large-scale phenomena. For example, the primary motivation of the aptly named STEREO mission (Kaiser et al. 2008) was to provide stereoscopic observations of the Sun and the inner heliosphere. The in-situ particle instruments of the PLASTIC suite were designed for studies of the temporal and spatial variations of ICMEs (Galvin et al. 2008). Likewise, the Waves investigation allowed for the triangulation (*radiogoniometry*) of radio-burst source regions (Bougeret et al. 2008, Sect. 3.4), which has also been achieved using spacecraft from separate missions (Steinberg et al. 1984; Hoang et al. 1998; Reiner et al. 1998).

Constellations of spacecraft with tighter spacings are used to observe local or small-scale plasma phenomena, especially in Earth’s magnetosphere and magnetosheath. This approach was largely pioneered with the Cluster mission (Escoubet et al. 1997) and later employed and expanded upon for THEMIS/ARTEMIS (Angelopoulos 2008) and MMS (Burch et al. 2016). In each of these missions, at least four spacecraft were flown in a quasi-tetrahedral formation to utilize three basic techniques (Dunlop et al. 1988):

- In *curlometry*, a four-point measurement of the magnetic field $\mathbf{B}$ is used to estimate $\varvec{\nabla }\times \mathbf{B}$ and thereby the current density $\mathbf{j}$ (Robert et al. 2000). This technique relies on $\mathbf{j}$ being nearly uniform within the tetrahedron, so it is best suited to study phenomena on spatial scales of order or larger than the dimension of the constellation.
- For the *wave-telescope* technique, a Fourier analysis of $\mathbf{B}$-measurements from the four spacecraft is made to determine the frequency spectrum, directional distribution, and mode of plasma fluctuations (Neubauer and Glassmeier 1990; Pinçon and Motschmann 2000; Motschmann et al. 2000). Due to effects such as aliasing, this method is most accurate in characterizing waves comparable in scale to the spacecraft constellation (Sahraoui et al. 2010a).
- In a *discontinuity analysis*, the arrival times of a magnetic discontinuity (e.g., a shock) at the spacecraft are compared so that the discontinuity’s orientation and velocity can be inferred (Russell et al. 1983; Mottez and Chanteur 1994; Dunlop and Woodward 2000). This method is most accurate for discontinuities whose boundary regions are thin relative to the spacecraft separations.

## Coulomb collisions

Collisions among particles provide the fundamental mechanism through which an ionized or neutral gas increases its entropy and ultimately comes into thermal equilibrium. In a fully ionized plasma, hard scatterings rarely occur; instead, *Coulomb collisions*, in which charged particles slightly deflect each other, are the primary collisional means by which particles exchange momentum and energy. The solar wind’s low density ensures that the rates of particle collisions remain relatively low. In contrast, the denser plasma of the solar corona has a much higher collision rate, and collisional processes are understood to be an important ingredient in the heating and acceleration of coronal plasma (see Sect. 3.1). Unfortunately, this has led to the widespread misconception that, beyond the solar corona, Coulomb collisions have no impact on the evolution of solar-wind plasma. In reality, while collision rates in the solar wind can be very low, the effects of collisions on the plasma never truly vanish.

This section overviews the effects that Coulomb collisions have on the microkinetics and large-scale evolution of solar-wind plasma through interplanetary space. Section 3.1 provides a simple dimensional analysis of Coulomb collisions, while Sect. 3.2 overviews the more complete kinetic theory of particle collisions in plasmas. Section 3.3 describes observations of solar-wind collisional relaxation.

### Dimensional analysis of Coulomb collisions

Before addressing the detailed kinetic treatment of collisions, we use dimensional analysis to derive a very rough expression for the rate of collisions in a plasma among particles of the same species.

We consider a species whose particles have mass $m_j$ and charge $q_j$. The *j*-particles may be approximated as all traveling at the species’ thermal speed $w_j$. When a pair of *j*-particles collide, kinetic energy is temporarily converted into electric potential energy. Assuming (very crudely) that this conversion is complete,

89 $$\begin{aligned} 2\left( \frac{1}{2} m_j w_j^2\right) = \frac{q_j^2}{x_{\min }}, \end{aligned}$$

where $x_{\min }$ is the particles’ distance of closest approach. Consequently,

90 $$\begin{aligned} \sigma \equiv \pi x_{\min }^2 = \frac{\pi q_j^4}{m_j^2 w_j^4} \end{aligned}$$

is the scattering cross-section for collisions among *j*-particles.

We now consider a volume *V* containing $N_j$ of the *j*-particles. The average time $t_j$ that a *j*-particle goes between collisions is roughly equal to the time that it takes to sweep out $1/N_j$ of the total volume. Taking $\sigma$ to be the particle’s effective cross-sectional area,

91 $$\begin{aligned} \frac{1}{n_j} = \frac{V}{N_j} = \sigma w_j t_j, \end{aligned}$$

where $n_j$ is the number density of *j*-particles. Thus,

92 $$\begin{aligned} t_j = \frac{1}{n_j w_j \sigma } = \frac{m_j^2 w_j^3}{\pi q_j^4 n_j} = \frac{2^{3/2}m_j^{1/2}\left( k_{\mathrm {B}} T_j\right) ^{3/2}}{\pi q_j^4 n_j}. \end{aligned}$$

Though Eq. (92) was derived from a naïve treatment of Coulomb collisions, it can be used to approximate the collisionality of a species such as protons. For example, at $r = 1\,\mathrm{au}$ from the Sun, $n_{\mathrm {p}} \sim 3\,\mathrm{cm}^{-3}$ and $T_{\mathrm {p}} \sim 10^5\,\mathrm{K}$. These correspond to a proton collisional timescale of $t_{\mathrm {p}} \sim 10^8\,\mathrm{s}$, which is substantially longer than the solar wind’s typical expansion time to this distance; see Eq. (1). In contrast, in the middle corona (see Fig. 2), $n_{\mathrm {p}}\sim 10^8\,\mathrm{cm}^{-3}$ and $T_{\mathrm {p}}\sim 10^6\,\mathrm{K}$, which give $t_{\mathrm {p}}\sim 350\,\mathrm{s}$. These estimates, though very rough, reveal that collisional effects have substantially more impact on coronal versus solar-wind plasma.

The stark difference in collisionality between the solar corona and solar wind forms the basis of *exospheric models* of the heliosphere. Although these models fall beyond the scope of this review, they warrant some mention. Since the early work on exospheric models by Jockers (1968, 1970) and Lemaire and Scherer (1971a, b), they have been shown to account for some features of the interplanetary solar wind. For example, the preferential heating of minor ions in a coronal exosphere can lead to the preferential acceleration of these ions (Pierrard et al. 2004). Maksimovic et al. (2005) offer a more complete overview of exospheric models, and the reviews by Marsch (1994) and Echim et al. (2011) provide an even more detailed treatment of the subject.

### Kinetic theory of collisions

A full treatment of the kinetic theory of collisions in plasmas is beyond the scope of this review. Instead, this section serves as a brief description of how the collisional term of the Boltzmann equation is used to derive collision rates for particle moments. More complete presentations of the theory are given by Spitzer (1956), Longmire (1963), Braginskii (1965), Wu (1966), Burgers (1969), Krall and Trivelpiece (1973, Chapters 6 and 7), Schunk (1975, 1977), Lifshitz and Pitaevskii (1981, Chapter 4), Klimontovich (1997), and Fitzpatrick (2015).

#### The collision term

Discussions of particle collisions in gases usually begin with the Boltzmann equation (19) since the effects of collisions are neatly grouped into the *collision term* on the right-hand side of the equation:

93 $$\begin{aligned} \frac{\partial f_j}{\partial t}+\mathbf{v}\cdot \frac{\partial f_j}{\partial \mathbf{x}}+\mathbf{a}\cdot \frac{\partial f_j}{\partial \mathbf{v}}=\left( \frac{\delta f_j}{\delta t}\right) _{\mathrm {c}}, \end{aligned}$$

where the derivative $\left( \delta /\delta t\right) _{\mathrm {c}}$ is known as the *collision operator*. The separation of the collision term from the terms on the left-hand side becomes somewhat murky for plasmas. Coulomb collisions occur through the interaction of the *particle electric fields*, but the plasma’s *background electric field* contributes to the acceleration $\mathbf{a}$. The particle electric field is the field generated by a single particle, while the background electric field is the collective result of all neighboring charged particles. Ultimately, the distinction between collisions and the effects of the background fields is phenomenological. Under the *molecular chaos hypothesis* (or *stoßzahlansatz*), collisions among particles are assumed to be uncorrelated and to occur randomly (Maxwell 1867).

**Fig. 12:**
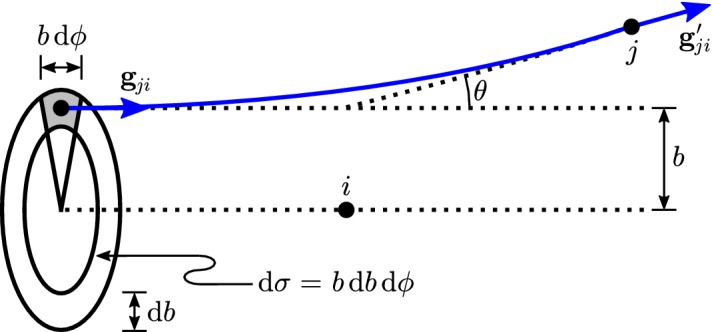
Diagram of a *j*-particle scattering off of an *i*-particle via the electric force in the *i*-particle’s reference frame, in which the *j*-particle has an initial velocity $\mathbf{g}_{ji}$ and a final velocity $\mathbf{g}'_{ji}$; see Eqs. (95) and (96)

To derive an expression for the collisional term, we consider the Coulomb scattering of a *j*-particle off of an *i*-particle via the electric force. We define the particles’ initial velocities as $\mathbf{v}_j$ and $\mathbf{v}_i$, their final velocities as $\mathbf{v}^{\prime }_j$ and $\mathbf{v}^{\prime }_i$, their masses as $m_j$ and $m_i$, and their charges as $q_j$ and $q_i$. We note that the *j*- and *i*-particles may be of the same species. The center-of-mass velocity of the two particles is

94 $$\begin{aligned} \mathbf{u}_{ji} \equiv \frac{m_j\mathbf{v}_j + m_i\mathbf{v}_i}{m_j + m_i} = \frac{m_j\mathbf{v}^{\prime }_j + m_i\mathbf{v}^{\prime }_i}{m_j + m_i} \equiv \mathbf{u}^{\prime }_{ji}, \end{aligned}$$

which is unchanged by the collision. Figure 12 depicts this scattering event in the *i*-particle’s frame of reference, in which the *j*-particle has an initial velocity

95 $$\begin{aligned} \mathbf{g}_{ji} \equiv \mathbf{v}_j - \mathbf{v}_i \end{aligned}$$

and a final velocity

96 $$\begin{aligned} \mathbf{g}^{\prime }_{ji} \equiv \mathbf{v}^{\prime }_j - \mathbf{v}^{\prime }_i. \end{aligned}$$

We denote the impact parameter as *b* and the scattering angle as $\theta$. In a Coulomb collision, these two quantities are related by

97 $$\begin{aligned} \tan \left( \frac{\theta }{2}\right) = \frac{q_jq_i}{m_{ji}g_{ji}^2 b}, \end{aligned}$$

where

98 $$\begin{aligned} m_{ji} \equiv \frac{m_jm_i}{m_j+m_i} \end{aligned}$$

is the reduced mass of the two particles (see, e.g., Thornton and Marion 2004; Fitzpatrick 2015). We consider an infinitesimal portion of the impact-parameter plane (see Fig. 12) as

99 $$\begin{aligned} {\mathrm {d}}\sigma = b\,{\mathrm {d}}b\,{\mathrm {d}}\phi . \end{aligned}$$

All *j*-particles that originate from this region are scattered into an infinitesimal solid-angle centered on $\theta$:

100 $$\begin{aligned} {\mathrm {d}}\varOmega = \sin \theta \,{\mathrm {d}}\theta \,{\mathrm {d}}\phi . \end{aligned}$$

To derive the differential cross-section for a Coulomb collision, we assume that the colliding particles only interact electrostatically. Then, when we combine Eqs. (99) and (100) with that for the Coulomb force, we arrive at the *Rutherford cross-section* (Rutherford 1911; Geiger and Marsden 1913):

101 $$\begin{aligned} \frac{{\mathrm {d}}\sigma }{{\mathrm {d}}\varOmega } = \frac{q_j^2 q_i^2}{4 m_{ji}^2 g_{ji}^4\sin ^4(\theta /2)}. \end{aligned}$$

Now, we consider all *i*-particles in the infinitesimal volume of phase space ${\mathrm {d}}^3\mathbf{v}_i$ that is centered on $\mathbf{v}_i$. The rate (i.e., the number of particles per unit time) at which *j*-particles, originating from ${\mathrm {d}}\sigma$, collide with *i*-particles in ${\mathrm {d}}^3\mathbf{v}_i$ is

102 $$\begin{aligned} f_i(\mathbf{v}_i) g_{ji}\,{\mathrm {d}}\sigma \,{\mathrm {d}}^3\mathbf{v}_i = f_i(\mathbf{v}_i)g_{ji}\frac{{\mathrm {d}}\sigma }{{\mathrm {d}}\varOmega }{\mathrm {d}}\varOmega \,{\mathrm {d}}^3\mathbf{v}_i. \end{aligned}$$

Thus, the rate of decrease in the value of $f_j(\mathbf{v}_j)$ due to collisions with *i*-particles in all regions of phase space is

103 $$\begin{aligned} \left( \frac{\delta f_j}{\delta t}\right) _{{\mathrm {c}},i,-} = - \int {\mathrm {d}}^3\mathbf{v}_i \int {\mathrm {d}}\varOmega \,f_j(\mathbf{v}_j) f_i(\mathbf{v}_i) g_{ji} \frac{{\mathrm {d}}\sigma }{{\mathrm {d}}\varOmega }. \end{aligned}$$

The above expression is negative because it only accounts for the decrease in $f_j(\mathbf{v}_j)$ due to *j*-particles of velocity $\mathbf{v}_j$ being scattered to other velocities by *i*-particles. The value of $f_j(\mathbf{v}_j)$ can also increase as collisions scatter *j*-particles of other velocities to $\mathbf{v}_j$. Indeed, Coulomb collisions are symmetric: if *j*- and *i*-particles of initial velocities $\mathbf{v}^{\prime }_j$ and $\mathbf{v}^{\prime }_i$ collide at an impact parameter *b*, their final velocities will be $\mathbf{v}_j$ and $\mathbf{v}_i$. Thus, the rate of increase in $f_j(\mathbf{v}_j)$ due to collisions with *i*-particles is

104 $$\begin{aligned} \left( \frac{\delta f_j}{\delta t}\right) _{{\mathrm {c}},i,+} = \int {\mathrm {d}}^3\mathbf{v}_i \int {\mathrm {d}}\varOmega \,f_j(\mathbf{v}^{\prime }_j) f_i(\mathbf{v}^{\prime }_i) g_{ji} \frac{{\mathrm {d}}\sigma }{{\mathrm {d}}\varOmega }. \end{aligned}$$

We note that, in the above equation, $\mathbf{v}^{\prime }_j$ and $\mathbf{v}^{\prime }_i$ are functions of $\mathbf{v}_j$, $\mathbf{v}_i$, and $\theta$. The net rate of change in $f_j(\mathbf{v}_j)$ due to collisions with *i*-particles is

105 $$\begin{aligned} \left( \frac{\delta f_j}{\delta t}\right) _{{\mathrm {c}},i}= & {} \left( \frac{\delta f_j}{\delta t}\right) _{{\mathrm {c}},i,+} + \left( \frac{\delta f_j}{\delta t}\right) _{{\mathrm {c}},i,-}\nonumber \\= & {} \int {\mathrm {d}}^3\mathbf{v}_i \int {\mathrm {d}}\varOmega \left[ f_j(\mathbf{v}^{\prime }_j) f_i(\mathbf{v}^{\prime }_i) - f_j(\mathbf{v}_j) f_i(\mathbf{v}_i) \right] g_{ji} \frac{{\mathrm {d}}\sigma }{{\mathrm {d}}\varOmega }. \end{aligned}$$

Finally, the net rate of change in $f_j(\mathbf{v}_j)$ due to collisions with all species (i.e., the full collision term) is

106 $$\begin{aligned} \left( \frac{\delta f_j}{\delta t}\right) _{\mathrm {c}}= & {} \sum _{i} \left( \frac{\delta f_j}{\delta t}\right) _{{\mathrm {c}},i} \nonumber \\= & {} \sum _{i} \int {\mathrm {d}}^3\mathbf{v}_i \int {\mathrm {d}}\varOmega \left[ f_j(\mathbf{v}^{\prime }_j) f_i(\mathbf{v}^{\prime }_i) - f_j(\mathbf{v}_j) f_i(\mathbf{v}_i) \right] g_{ji}\frac{{\mathrm {d}}\sigma }{{\mathrm {d}}\varOmega }. \end{aligned}$$

This includes Coulomb collisions of *j*-particles with other *j*-particles, so the above sum must include $i = j$.

#### The Landau collision integral

Evaluating Eq. (106) is highly non-trivial but it is helped by the fact that the dominant contribution comes from small-angle collisions: those that produce small $\theta$-values. Before invoking the small-$\theta$ limit, it is convenient to express the particles’ initial and final velocities in terms of the center-of-mass velocity $\mathbf{u}_{ji} = \mathbf{u}^{\prime }_{ji}$ as

107 $$\begin{aligned} \mathbf{v}_j=\mathbf{u}_{ji}+\frac{m_{ji}}{m_j} \mathbf{g}_{ji}, \end{aligned}$$

108 $$\begin{aligned} \mathbf{v}^{\prime }_j=\mathbf{u}_{ji}+\frac{m_{ji}}{m_j}\mathbf{g}^{\prime }_{ji}, \end{aligned}$$

109 $$\begin{aligned} \mathbf{v}_i=\mathbf{u}_{ji}-\frac{m_{ji}}{m_i}\mathbf{g}_{ji}, \end{aligned}$$

and

110 $$\begin{aligned} \mathbf{v}^{\prime }_i=\mathbf{u}_{ji}-\frac{m_{ji}}{m_i}\mathbf{g}^{\prime }_{ji}. \end{aligned}$$

Thus,

111 $$\begin{aligned} \mathbf{v}^{\prime }_j = \mathbf{v}_j + \frac{m_{ji}}{m_j}\varDelta \mathbf{g}_{ji} \end{aligned}$$

and

112 $$\begin{aligned} \mathbf{v}^{\prime }_i = \mathbf{v}_i - \frac{m_{ji}}{m_i}\varDelta \mathbf{g}_{ji}, \end{aligned}$$

where

113 $$\begin{aligned} \varDelta \mathbf{g}_{ji} \equiv \mathbf{g}^{\prime }_{ji} - \mathbf{g}_{ji}. \end{aligned}$$

In the small-$\theta$ limit, $\left|\varDelta \mathbf{g}_{ji}\right|$ is also small, so Eqs. (111) and (112) can be used as the basis for a Taylor expansion of $f_j$ and $f_i$ about $\mathbf{v}=\mathbf{v}_j$ and $\mathbf{v}=\mathbf{v}_i$, respectively. Retaining terms through the second order gives

114 $$\begin{aligned} f_j(\mathbf{v}^{\prime }_j) \approx f_j(\mathbf{v}_j) + \frac{m_{ji}}{m_j}\varDelta \mathbf{g}_{ji}\cdot \frac{\partial f_j}{\partial \mathbf{v}_j}+ \frac{m_{ji}^2}{2 m_j^2} \varDelta \mathbf{g}_{ji}\,\varDelta \mathbf{g}_{ji}:\frac{\partial ^2 f_j}{\partial \mathbf{v}_j\partial \mathbf{v}_j} \end{aligned}$$

and

115 $$\begin{aligned} f_i(\mathbf{v}^{\prime }_i) \approx f_i(\mathbf{v}_i) - \frac{m_{ji}}{m_i} \varDelta \mathbf{g}_{ji}\cdot \frac{\partial f_i}{\partial \mathbf{v}_i} + \frac{m_{ji}^2}{2 m_i^2}\varDelta \mathbf{g}_{ji}\,\varDelta \mathbf{g}_{ji}:\frac{\partial ^2 f_i}{\partial \mathbf{v}_i\partial \mathbf{v}_i}. \end{aligned}$$

These approximations can be substituted into Eq. (105), which, after considerable simplification (see, e.g., Hellinger and Trávníček 2009; Fitzpatrick 2015), yields the *Landau collision integral/operator* (Landau 1936, 1937):

116 $$\begin{aligned} \left( \frac{\delta f_j}{\delta t}\right) _{{\mathrm {c}},i}\approx & {} \frac{2 \pi q_j^2 q_i^2}{m_j} \ln \varLambda _{ji}\nonumber \\&\times \frac{\partial }{\partial \mathbf{v}_j}\cdot \left[ \int {\mathrm {d}}^3\mathbf{v}_i \frac{{\mathsf {I}}_3\,g_{ji}^2-\mathbf{g}_{ji} \mathbf{g}_{ji}}{g_{ji}^3}\cdot \left( \frac{f_i(\mathbf{v}_i)}{m_j}\frac{\partial f_j}{\partial \mathbf{v}_j} - \frac{f_j(\mathbf{v}_j)}{m_i}\frac{\partial f_i}{\partial \mathbf{v}_i} \right) \right] ,\nonumber \\ \end{aligned}$$

where $\ln \varLambda _{ji}$ is the *Coulomb logarithm*, which is the subject of Sect. 3.2.3 and is given in Eq. (117).

Although Eq. (116) is an improvement over Eq. (105), actually calculating the Landau collision integral remains a daunting task even for relatively simple scenarios. Often, additional approximations are introduced, and numerical methods are employed. An alternative approach is the *BGK operator*, which explicitly models the departure of a particle species’ distribution function from its equilibrium state (Bhatnagar et al. 1954). This method was later generalized for the case of magnetized plasmas (Dougherty 1964, and references therein). Pezzi et al. (2015) present a numerical comparison of the Landau and Dougherty collision operators.

#### The Coulomb logarithm

The factor $\ln \varLambda _{ji}$ in Eq. (116) is known as the *Coulomb logarithm*:

117 $$\begin{aligned} \ln \varLambda _{ji} \equiv \int \limits _{b_{ji,\min }}^{b_{ji,\max }} \frac{{\mathrm {d}}b}{b} = \ln \left( \frac{b_{ji,\max }}{b_{ji,\min }}\right) . \end{aligned}$$

It arises from the $\varOmega$-integral in Eq. (105) via the relationship between *b* and $\theta$ according to Eq. (97). Even though the derivation of Eq. (116) would seemingly imply that all *b* from 0 to $\infty$ should be considered, the Coulomb logarithm diverges at both of these limits. As a result, the integral in Eq. (117) has been given the more restrictive limits $b_{ji,\min }$ and $b_{ji,\max }$, which are discussed below. Though there is some degree of arbitrariness in how these limits are defined, Eq. (117) is relatively insensitive to their particular values. In practice, $b_{ji,\min } \ll b_{ji,\max }$, so the logarithm of their ratio only changes appreciably when they are varied by orders of magnitude.

The integral in Eq. (117) diverges at small *b* due to the breakdown of the small-$\theta$ limit used to derive Eq. (116): as the value of *b* decreases, the value of $\theta$ increases until it can no longer be considered small. In reality, collisions with small *b* have a minimal effect on the distribution function because of their relative rarity. As a result, collisions with $\theta > \theta _{\max }$ are negligible and may be safely disregarded. A typical choice is $\theta _{\max } = 90^{\circ }$, which, by Eq. (97), corresponds to

118 $$\begin{aligned} b_{ji,\min } = \frac{q_jq_i}{m_{ji}{\overline{g}}_{ji}^2}, \end{aligned}$$

where ${\overline{g}}_{ji}$ is the average speed of a *j*-particle relative to an *i*-particle. The quantity $m_{ji}{\overline{g}}_{ji}^2$ roughly reflects the average kinetic energy of *j*- and *i*-particles in the plasma frame. As a result,

119 $$\begin{aligned} b_{ji,\min } = \frac{q_jq_i}{k_{\mathrm {B}}T_{ji}}, \end{aligned}$$

where $T_{ji}$ is the average temperature of the *j*- and *i*-particles.

The divergent behavior of Eq. (117) at high *b* stems from a more subtle reason. The analysis above begins by considering the scattering of a single particle by another. Effectively, the motion of each particle is modeled as a series of hard scatters, between which the particle’s velocity remains constant. In reality, Coulomb collisions are soft scatters, and each plasma particle is simultaneously colliding with many other particles. As a result, each particle is partially shielded from the influence of distant particles by the particles closer to it. An appropriate choice, then, for $b_{ji,\max }$ is the Debye length $\lambda _{\mathrm {D}}$ (Cohen et al. 1950; Spitzer 1956) as defined in Eq. (11). Taking into account all the particle species in the plasma,

120 $$\begin{aligned} b_{ji,\max } = b_{\max } \equiv \left( \frac{4\pi }{k_{\mathrm {B}}} \sum \limits _\ell \frac{q_\ell ^2 n_\ell }{T_\ell } \right) ^{-1/2}, \end{aligned}$$

where $q_\ell$, $n_\ell$, and $T_\ell$ are the charge, number density, and temperature of each species in the plasma. As a result of this choice, the value of $b_{ji,\max }$ is the same for all pairs of particle species.

This discussion of $b_{ji,\max }$ raises some concern over the use of binary collisions at all. In principle, a more accurate approach would be to use an analysis of Markovian processes to derive the collision operator from the Fokker–Planck equation (Fokker 1914; Planck 1917). Nevertheless, Wu (1966, Sects. 2–6) notes that both analyses produce the same result, Eq. (116), in the limit of small-angle scattering.

#### Rosenbluth potentials

An alternative expression for the Landau collision integral in Equation (116) can be obtained by using the *Rosenbluth potentials* (Rosenbluth et al. 1957), which are defined as

121 $$\begin{aligned} G_i(\mathbf{v}_j) \equiv \int \left| \mathbf{g}_{ji} \right| f_i(\mathbf{v}_i)\, {\mathrm {d}}^3 \mathbf{v}_i \end{aligned}$$

and

122 $$\begin{aligned} H_i(\mathbf{v}_j) \equiv \int \frac{1}{\left| \mathbf{g}_{ji}\right| } f_i(\mathbf{v}_i)\, {\mathrm {d}}^3\mathbf{v}_i. \end{aligned}$$

Likewise, we define flux densities associated with friction

123 $$\begin{aligned} \mathbf{A}_{ji} \equiv \frac{4 \pi q_j^2 q_i^2}{m_i}\ln \varLambda _{ji}\frac{\partial H_i}{\partial \mathbf{v}_j} \end{aligned}$$

and with diffusion

124 $$\begin{aligned} {\mathsf {D}}_{ji} \equiv \frac{2 \pi q_j^2 q_i^2}{m_j} \ln \varLambda _{ji} \frac{\partial ^2 G_i}{\partial \mathbf{v}_j\partial \mathbf{v}_j}. \end{aligned}$$

With these quantities defined, we express the Landau collision operator as the velocity divergence of the sum of these fluxes (see Montgomery and Tidman 1964; Marsch 2006; Fitzpatrick 2015), casting it in terms of a Fokker–Planck advection–diffusion equation in velocity space:

125 $$\begin{aligned} \left( \frac{\delta f_j}{\delta t}\right) _{{\mathrm {c}},i} \approx - \frac{1}{m_j}\frac{\partial }{\partial \mathbf{v}_j}\cdot \left( \mathbf{A}_{ji} - {{\mathsf {D}}}_{ji} \cdot \frac{\partial }{\partial \mathbf{v}_j} \right) f_j. \end{aligned}$$

#### Collisional timescales

Conceptually, a *collisional timescale* is the time required for collisions to significantly reduce a non-equilibrium feature such as a drift or anisotropy (for examples of non-equilibrium kinetic features in the solar wind, see Sects. 1.4.4, 1.4.5). Each specific type of non-equilibrium feature has its own expression for its collisional timescale that depends on the conditions in the plasma. These timescales are derived from moments of the Boltzmann collision term, similar to the procedure described in Sect. 1.4.1. This requires that assumptions be made about the particular form of the distribution function of each particle species involved.

As an example, we discuss the *collisional slowing time* for two particle species, *j* and *i*.^11^ These species’ differential flow is

126 $$\begin{aligned} \varDelta \mathbf{U}_{ji} \equiv \mathbf{U}_j - \mathbf{U}_i, \end{aligned}$$

where $\mathbf{U}_j$ and $\mathbf{U}_i$ are the bulk velocities of species *j* and *i*, respectively. Then, the rate of change in the differential flow due to collisions is

127 $$\begin{aligned} \left( \frac{\delta \left( \varDelta \mathbf{U}_{ji}\right) }{\delta t} \right) _{\mathrm{c}} = \left( \frac{\delta \mathbf{U}_j}{\delta t} \right) _{\mathrm{c}} - \left( \frac{\delta \mathbf{U}_i}{\delta t} \right) _{\mathrm{c}}. \end{aligned}$$

We express the bulk velocities $\mathbf{U}_j$ and $\mathbf{U}_i$ as moments of $f_j$ and $f_i$, the distribution functions of the *j*- and *i*-particles, according to Eq. (28) and find

128 $$\begin{aligned} \left( \frac{\delta \left( \varDelta \mathbf{U}_{ji}\right) }{\delta t} \right) _{\mathrm {c}}= & {} \left[ \frac{\delta }{\delta t} \left( \frac{1}{n_j} \int {\mathrm {d}}^3\mathbf{v}\,\mathbf{v} f_j(\mathbf{v}) \right) \right] _{\mathrm {c}} - \left[ \frac{\delta }{\delta t} \left( \frac{1}{n_i} \int {\mathrm {d}}^3\mathbf{v}\,\mathbf{v}\,f_i(\mathbf{v}) \right) \right] _{\mathrm{c}} \nonumber \\= & {} \int {\mathrm {d}}^3\mathbf{v} \, \mathbf{v} \left[ \frac{1}{n_j} \left( \frac{\delta f_j}{\delta t} \right) _{\mathrm {c}} - \frac{1}{n_i} \left( \frac{\delta f_i}{\delta t} \right) _{\mathrm {c}} \right] . \end{aligned}$$

To continue this analysis, we must make a choice for the form of the collision terms and for the distribution functions. Once these are set, the result, to first order, has the form

129 $$\begin{aligned} \left( \frac{\delta \left( \varDelta \mathbf{U}_{ji}\right) }{\delta t} \right) _{\mathrm {c}} = -\, \nu _{\mathrm{s},ji} \, \varDelta \mathbf{U}_{ji}, \end{aligned}$$

where $\nu _{\mathrm{s},ji}$ is the *collision frequency* for the slowing of *j* particles by *i* particles. The corresponding collisional timescale is defined to be

130 $$\begin{aligned} \tau _{\mathrm{s},ji} \equiv \frac{1}{\nu _{\mathrm{s},ji}}. \end{aligned}$$

Collisional timescales are most commonly derived and used for the relaxation of temperature anisotropy $$T_{\perp j}/T_{\parallel j}$$, unequal temperatures $$ T_{j}/T_{i}$$, and differential flow $$\varDelta \mathbf{U}_{ji}$$.

Specific expressions for these collisional timescales have been computed and/or compiled by Spitzer (1956), Schunk (1975, 1977), Hernández and Marsch (1985), Huba (2016), and Wilson et al. (2018). Typically, only one type of non-equilibrium feature is considered in each collisional timescale but formulæ derived by Hellinger and Trávníček (2009, 2010) consider all three of the features listed above. Hellinger (2016) uses observations from the Wind spacecraft to demonstrate that they result in substantially different collision and heating rates. Likewise, although most derivations assume Maxwellian or bi-Maxwellian distribution functions, Marsch and Livi (1985) derive timescales for $\kappa$-distributions.

#### Coulomb number and collisional age

The majority of the heating and acceleration that gives rise to the solar wind’s non-equilibrium properties occurs in and around the solar corona. Beyond that region, the solar wind’s bulk velocity $\mathbf{U}$ remains approximately constant and radial (see, e.g., Hellinger et al. 2011, 2013). Thus, the time required for a parcel of plasma to travel from the photosphere to a distance *r* is approximately the *expansion time* according to Eq. (1):

131 $$\begin{aligned} \tau = \frac{r}{U_r}. \end{aligned}$$

The *Coulomb number* of the parcel of plasma is then defined as

132 $$\begin{aligned} N_{\mathrm {c}} \equiv \frac{\tau }{\tau _{\mathrm {c}}} = \frac{r}{U_r \tau _{\mathrm {c}}}, \end{aligned}$$

where $\tau _{\mathrm{c}}$ is a collisional timescale. Notwithstanding the caveats noted below, the Coulomb number essentially approximates the number of collisional timescales that elapsed in a parcel of plasma during its journey from the Sun to an observer. In *collisionally old* ($N_{\mathrm {c}} \gg 1$) plasma, collisional equilibration has proceeded much farther than in *collisionally young* ($N_{\mathrm {c}} \ll 1$) plasma.

Although the Coulomb number has seen wide use in the analysis of solar-wind observations (see Sect. 3.3), the concept carries significant limitations. The above definition for $N_{\mathrm {c}}$ only allows for a single collision timescale $\tau _{\mathrm {c}}$. While the correct formula for $\tau _{\mathrm {c}}$ can be chosen for the non-equilibrium feature under consideration, accounting for the interactions of multiple departures from equilibrium presents difficulties. More fundamentally, the expression for $N_{\mathrm {c}}$ tacitly assumes that $\tau _{\mathrm {c}}$ remains constant with distance *r* from the Sun. In reality, $\tau _{\mathrm {c}}$ depends on density and temperature, both of which have strong radial trends.

To address some of these issues, various studies (Hernández et al. 1987; Chhiber et al. 2016; Kasper et al. 2017; Kasper and Klein 2019) employ an integrated Coulomb number of the form

133 $$\begin{aligned} A_{\mathrm {c}}\equiv \int \frac{\mathrm {d} t}{\tau _{\mathrm {c}}} = \int \frac{\mathrm {d} r}{U_r(r) \tau _{\mathrm {c}}(r)}. \end{aligned}$$

This formulation directly accounts for the radial dependences of densities, velocities, and temperatures. These radial trends can either be derived from theoretical expectations (e.g., for quasi-adiabatic expansion) or from empirical observations. Some authors (e.g., Kasper et al. 2017) differentiate between the *Coulomb number* $N_{\mathrm {c}}$ and *collisional age* $A_{\mathrm {c}}$, with the former defined by Eq. (132) and the latter defined by Eq. (133).^12^

Maruca et al. (2013) introduce a close alternative to the Coulomb-number analysis, *retrograde collisional analysis*, in which collisional timescales and radial trends are used to “undo” the effects of collisions and estimate the state of the solar wind when it was closer to the Sun.

### Observations of collisional relaxation in the solar wind

This section summarizes observational studies of collisional relaxation’s effects on solar-wind plasma as it expands through the heliosphere.

#### Ion collisions

Early observations of solar-wind ions indicate that $\alpha$-particles tend to be significantly faster and hotter than protons (see Sect. 1.4.4). Observations from IMP 6, IMP 7, IMP 8, and OGO 5 (Feldman et al. 1974a; Neugebauer 1976; Neugebauer and Feldman 1979) demonstrate that the values of $\left|\varDelta \mathbf{U}_{\alpha {\mathrm {p}}} \right|$ and $T_{\alpha }/T_{\mathrm {p}}$ decrease toward 0 and 1 with increasing $N_{\mathrm {c}}$. This negative correlation indicates that $\alpha$-particles are first preferentially accelerated and heated in the corona and then partially equilibrate with protons as the plasma expands through the inner heliosphere. Later studies using observations from Helios (Marsch et al. 1982a, 1983; Livi et al. 1986), ISEE 3 (Klein et al. 1985), Prognoz 7 (Yermolaev et al. 1989, 1991; Yermolaev and Stupin 1990), Ulysses (Neugebauer et al. 1994), and Wind (Kasper et al. 2008, 2017; Maruca et al. 2013; Hellinger 2016) confirm these early results. Interplanetary coronal mass ejections (ICMEs) are a notable exception to this overall trend in that they exhibit enhancements in $T_{\alpha }/T_{\mathrm {p}}$, which arise from ongoing heating during expansion (Liu et al. 2006).

Measurements of $T_{\perp {\mathrm {p}}}$ and $T_{\parallel {\mathrm {p}}}$ from Wind reveal that the average value of the anisotropy ratio $T_{\perp {\mathrm {p}}}/T_{\parallel {\mathrm {p}}}\rightarrow 1$ as the Coulomb number increases (Kasper et al. 2008, 2017). Further observations (Bale et al. 2009) show that both Coulomb collisions and kinetic microinstabilities (see Sect. 6) have roles in limiting proton temperature anisotropy. Numerical models confirm this interplay of collisional and wave–particle effects (Tam and Chang 1999; Hellinger and Trávníček 2010; Matteini et al. 2012).

**Fig. 13:**
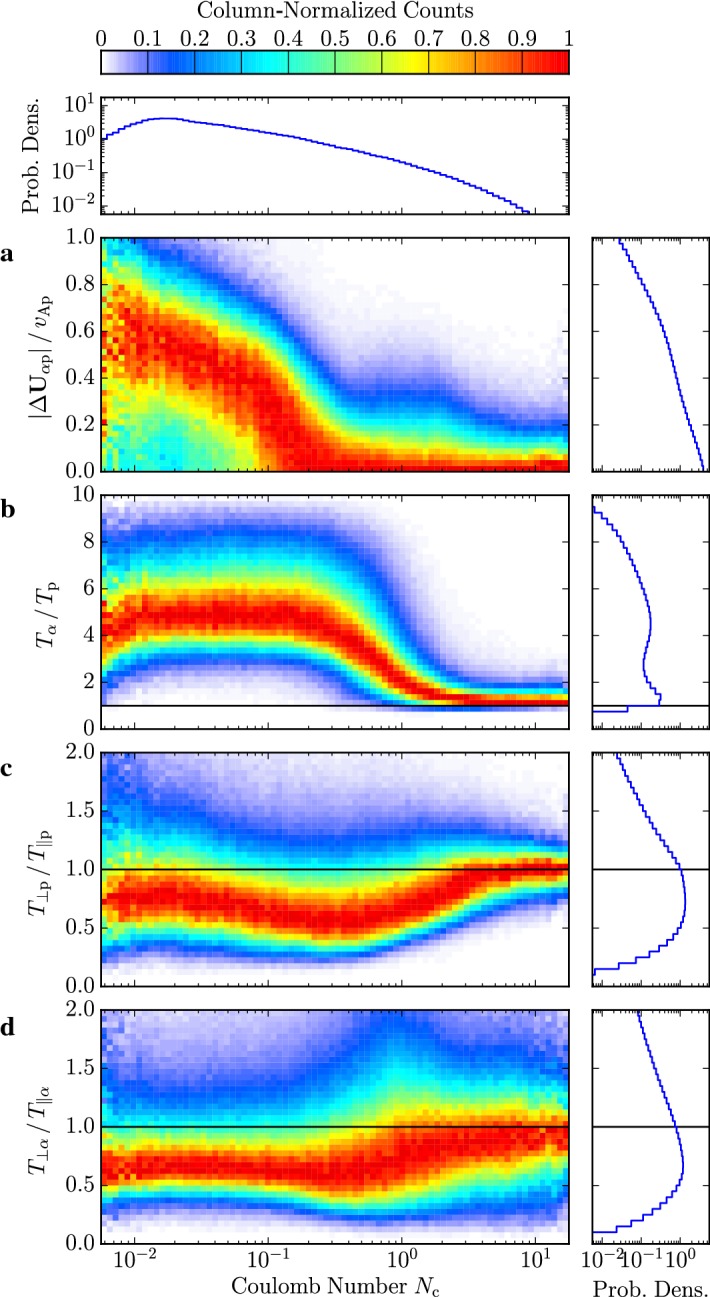
Trends in four parameters with Coulomb number $N_{\mathrm {c}}$: **a** $\alpha$–proton differential flow normalized to the proton Alfvén speed, **b** $\alpha$-to-proton relative temperature, **c** proton temperature anisotropy, and **d** $\alpha$-particle temperature anisotropy. The dataset, compiled by Maruca et al. (2012, 2013), consists of 2.1-million data from the Wind/SWE Faraday cups. The color scale is linear, and red indicates the most-likely parameter value for a given $N_{\mathrm {c}}$-value. The probability densities of Coulomb number (top) and of each of the four parameters (right) are also shown. After Kasper et al. (2008, 2017)

Figure 13 shows trends in four parameters with Coulomb number $N_{\mathrm {c}}$ in a dataset of 2.1-million data from the Wind/SWE Faraday cups compiled by Maruca et al. (2012, 2013). The values of $N_{\mathrm {c}}$ are calculated using the expression derived by Maruca et al. (2013), which is based on the proton “self-collision time” described by Spitzer (1956). For each parameter *P*, the $(N_{\mathrm {c}},P)$-plane is divided into 80 logarithmically spaced $N_{\mathrm {c}}$-bins and 40 linearly spaced *P*-bins. Once the data are binned, the grid is column-normalized: the number of counts in each bin is divided by the number of counts in the most-populated bin in its column. Thus, the color of each bin in Fig. 13 indicates the relative likelihood of a *P*-value for a given $N_{\mathrm {c}}$-value. Each of the four parameters in Fig. 13 is an indicator of a departure from local thermal equilibrium. As $N_{\mathrm {c}}$ increases, the most-likely *P*-value approaches its equilibrium state: 0 for $\left|\varDelta \mathbf{U}_{\alpha {\mathrm {p}}}\right|/v_{\mathrm {Ap}}$ and 1 for $T_{\alpha }/T_{\mathrm {p}}$, $T_{\perp \mathrm {p}}/T_{\parallel \mathrm {p}}$, and $T_{\perp \alpha }/T_{\parallel \alpha }$. Each parameter reaches equilibrium at a different $N_{\mathrm {c}}$-value because the formula for $N_{\mathrm {c}}$ uses the same self-collision time as a generic collisional timescale rather than the specific collisional timescale for each parameter *P*.

**Fig. 14:**
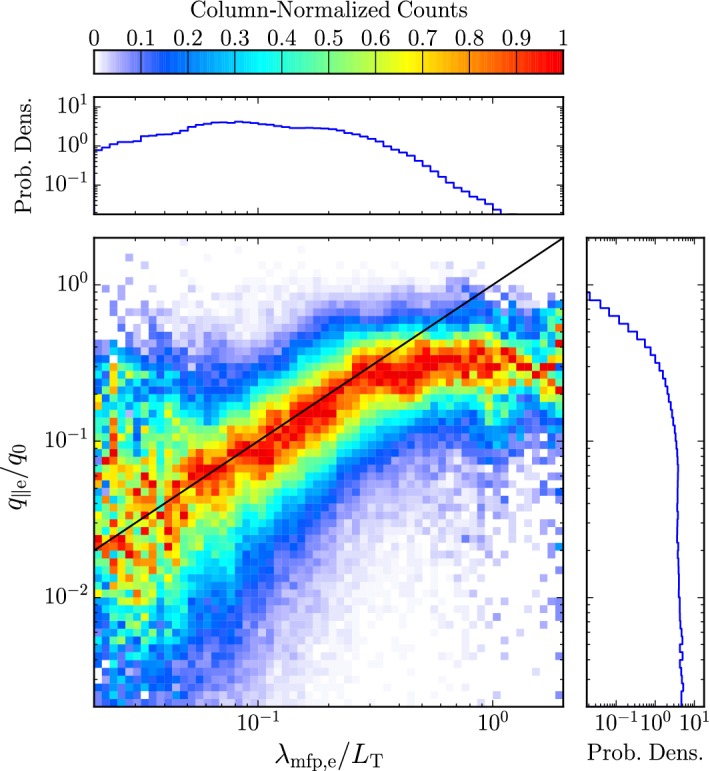
Column-normalized distribution of Wind/3DP electron data as a function of the parallel heat flux $q_{\parallel \mathrm {e}}$ and the electron mean free path $\lambda _{\mathrm {mfp,e}}$. The Spitzer–Härm prediction in this normalization is given by $q_{\parallel \mathrm {e}}/q_0=1.07\lambda _{\mathrm {mfp,e}}/L_{\mathrm {T}}$ and is shown as a black line. We use $\alpha =2/7$. The probability densities for $\lambda _{\mathrm {mfp,e}}/L_{\mathrm {T}}$ (top) and $q_{\parallel \mathrm {e}}/q_0$ (right) are also shown. After Salem et al. (2003) and Bale et al. (2013) and using data provided by C. Salem

Column-normalizing plots (as has been done, e.g., for those in Figs. 13, 14) is a powerful and well established technique for exploring collisional effects in solar-wind plasma. It represents a refinement of the method used in some of the earliest studies of collisional relaxation (e.g., Feldman et al. 1974a; Neugebauer 1976), in which data were divided into logarithmically uniform $N_{\mathrm {c}}$-intervals, and the average $T_{\alpha }/T_{\mathrm {p}}$-value was plotted for each interval. Nevertheless, some caution is warranted in producing and interpreting column-normalized plots in general. First, the procedure of column-normalization modifies the weights of different data points and thus may cause an overemphasis or underemphasis of bins in a statistical data set. Second, the very act of column-normalization imposes causality: the parameter on the vertical axis becomes a function of that on the horizontal axis. Though this is usually justified in collisionalization studies because of the strong theoretical motivation for such a causal relationship, column-normalization is not appropriate for all correlation studies. Third, determining which parameters to plot is complicated by the many correlations that exist among particle moments (e.g., the well established temperature–speed relationship for protons; Lopez and Freeman 1986). Even so, parameters such as $T_{\alpha }/T_{\mathrm {p}}$ and $\left| \varDelta \mathbf{U}_{\alpha \mathrm p}\right|$ have been qualitatively (Kasper et al. 2008) and quantitatively (Maruca et al. 2013) demonstrated to be more strongly correlated with $N_{\mathrm {c}}$ than with $n_{\mathrm {p}}$, $U_{\mathrm {p} r}$, or $T_{\mathrm {p}}$ (all three of which $N_{\mathrm {c}}$ depends on).

Observations also give insight into collisional effects on minor ions. ISEE 3 and SOHO/CELIAS data show that, while mass-proportional temperatures are most common, the effects of collisional thermalization are apparent at low solar-wind speeds (Bochsler et al. 1985; Hefti et al. 1998). Interestingly, von Steiger et al. (1995) and von Steiger and Zurbuchen (2006) find no indications of a departure from mass-proportional temperatures at any solar-wind speed. This may be due to the limited number of data from very slow wind or from the ongoing heating of heavy ions. Coulomb-number analyses of heavy-ion observations from ACE/SWICS show similar negative trends in the ion-to-proton temperature ratio with Coulomb number (Tracy et al. 2015, 2016).

Although most observational studies of ion–ion collisions focus on the effects of collisions on particle moments, some consider how collisions affect the structure of ion distribution functions. Marsch and Goldstein (1983) note that the value of the collision term in Eq. (106) varies across phase space and is highest for particles traveling at the bulk speed of the plasma. This finding is consistent with proton distribution functions observed by Helios, which show Maxwellian cores surrounded by non-Maxwellian tails. A kinetic model of the collisional effects on proton distribution functions counter-intuitively reveals that collisional isotropization can actually generate proton beams (Livi and Marsch 1987), which themselves would then be ultimately eroded by collisions.

#### Electron collisions

Collisions involving electrons, due to their higher rates (see, e.g., Wilson et al. 2018), are thought to play an even more important role in solar-wind thermodynamics than collisions involving only ions. As noted in Sect. 1.4.5, electron distribution functions in the solar wind typically exhibit a three-component structure consisting of a core, halo, and strahl. Many theories (e.g., Scudder and Olbert 1979a, b; Lie-Svendsen et al. 1997; Lie-Svendsen and Leer 2000) for the origin of these electron populations rely on the transition from highly collisional plasma in the lower corona to weakly collisional plasma in the upper corona.

Beyond the corona, numerous studies find that Coulomb collisions among electrons continue to affect them in the interplanetary solar wind. An analysis of Mariner 10 data (Ogilvie and Scudder 1978) reveals that collisions have the greatest influence on the electron core while the electron halo remains weakly collisional. Electron distribution functions observed by Helios show that Coulomb collisions have a significant impact on the phase-space location of the core–halo boundary (Pilipp et al. 1987a, b, c). Kinetic simulations suggest that the interplay of collisions and expansion in the solar wind can give rise to the electron core, halo, and beam (Landi et al. 2010, 2012). Moreover, a kinetic model for the radial evolution of the strahl developed by Horaites et al. (2018b) indicates that Coulomb collisions provide a significant source of pitch-angle scattering for this population.

Solar-wind electrons typically exhibit less temperature anisotropy than ions (Chen et al. 2016, Figure 1), which is at least partially ascribed to the higher rate of electron versus ion collisions. Analytical models that account for electron expansion and collisions in the interplanetary solar wind agree well with ISEE 3 and Ulysses observations of electron temperature anisotropy (Phillips et al. 1989a; Phillips and Gosling 1990; Phillips et al. 1993). A study of Wind observations by Salem et al. (2003) finds that electron temperature anisotropy is strongly correlated with Coulomb number, with collisionally old electrons being most likely to exhibit isotropy. As is the case for protons, data from Helios, Cluster, and Ulysses show that both Coulomb collisions and kinetic microinstabilities play significant roles in isotropizing solar-wind electrons (Štverák et al. 2008, 2015).

Collisions also significantly affect electron heat flux. According to *Spitzer–Härm theory* (Spitzer and Härm 1953), the electron heat flux is proportional to the timescale of electron–electron collisions. Statistical analyses of Wind electron measurements show that this relationship holds true but only in highly collisional plasma (Salem et al. 2003; Bale et al. 2013). Figure 14 shows the distribution of Wind/3DP electron data in the plane of the normalized parallel heat flux versus the normalized electron mean free path in the solar wind. We normalize $q_{\parallel \mathrm {e}}$ to the free-streaming saturation heat flux $q_0\equiv 3n_{\mathrm {e}}k_{\mathrm {B}}T_{\mathrm {e}}w_{\mathrm {e}}/2$ and $\lambda _{\mathrm {mfp,e}}$ to the temperature gradient $L_{\mathrm {T}}\equiv r/\alpha$, where *r* is the heliocentric distance of the measurement and $\alpha$ describes the observed temperature profile through $T_{\mathrm {e}}\propto r^{-\alpha }$. The dimensionless quantity $\lambda _{\mathrm {mfp,e}}/L_{\mathrm {T}}$ is called the *Knudsen number*. The black line shows the Spitzer–Härm prediction. The heat flux follows this prediction at large collisionality but deviates in the collisionless limit.

Spitzer–Härm theory is found to overestimate electron heat flux in moderately and weakly collisional plasma, which is consistent with results from the kinetic simulations of Landi et al. (2012) and Landi et al. (2014).

Occasionally, a parcel of solar-wind plasma is found to have an especially low or high rate of Coulomb collisions, which offers insight into the most extreme effects of collisions on electrons. In a study of several periods of very-low-density solar wind, each period exhibits an unusually narrow electron strahl (Ogilvie et al. 2000). This likely results from the combination of a low collision rate and the conservation of the first adiabatic invariant, given in Eq. (44), to first order as suggested by Fairfield and Scudder (1985). Conversely, data from ISEE 1 and ISEE 3 exhibit several *heat-flux dropouts* (Fitzenreiter and Ogilvie 1992): periods of very low electron heat flux. The weak electron halos observed during these dropouts likely result, at least in part, from enhanced electron collisionality. Likewise, Larson et al. (2000) and Farrugia et al. (2002), using the Wind and ACE spacecraft, identify weak halos in particularly dense and cold magnetic clouds and find them to be consistent with collisional effects.

## Plasma waves

*Plasma waves* are important processes for the transport and dissipation of energy in a plasma. They can accelerate plasma flows and heat plasma by damping. Section 4.1 introduces basic concepts to describe plasma waves. Section 4.2 describes damping and dissipation mechanisms, and Sect. 4.3 then presents types of plasma waves that are relevant to the multi-scale evolution of the solar wind. For more details on the broad topic of plasma waves, we refer to the excellent textbooks by Stix (1992) and Swanson (2003).

### Plasma waves as self-consistent electromagnetic and particle fluctuations

Waves are periodic or quasi-periodic spatio-temporal fluctuations which arise through the action of a *restoring force*. The self-consistent electromagnetic interactions in a plasma provide additional restoring forces that do not occur in a neutral gas. Therefore, a plasma can exhibit many more types of wave modes than a neutral gas. In this section, we introduce the linear theory of plasma waves. For further details on linear theory, we refer the reader to the general review on solar-wind plasma waves by Ofman (2010) and the textbooks by Stix (1992), Brambilla (1998), and Swanson (2003).

Linear wave theory considers a wave to be a fluctuating perturbation on an equilibrium state. We assume that any physical quantity *A* of the system can be written as

134 $$\begin{aligned} A(\mathbf{x},t)=A_0+\delta A(\mathbf{x},t), \end{aligned}$$

where $A_0$ is the constant background equilibrium, and $\delta A$ is the fluctuating perturbation of *A*. Moreover, we assume that the fluctuating quantities in a wave behave like

135 $$\begin{aligned} \delta A(\mathbf{x},t)= \mathrm {Re}\left[ A(\mathbf{k},\omega )\exp \left( i\mathbf{k}\cdot \mathbf{x}-i\omega t\right) \right] , \end{aligned}$$

where $A(\mathbf{k},\omega )$ is the complex Fourier amplitude of *A*, the wavevector $\mathbf{k}$ is real, and the frequency $\omega$ is complex. We define the *real frequency* as

136 $$\begin{aligned} \omega _{\mathrm {r}}\equiv \mathrm {Re}\,\omega \end{aligned}$$

and the *growth or damping rate* as

137 $$\begin{aligned} \gamma \equiv \mathrm {Im}\,\omega . \end{aligned}$$

The *linear dispersion relation* is a mathematical expression based on a self-consistent set of linearized equations for the plasma particles and the electromagnetic fields. It connects the wavevector $\mathbf{k}$ with the frequency $\omega$ in such a way that its solutions represent self-consistent waves in the plasma. If multiple solutions exist for a given $\mathbf{k}$, then each corresponds to a distinct *mode*. According to Eqs. (135) and (137), the amplitude of the fluctuations decreases exponentially with time if $\gamma < 0$. As a solution to the linear dispersion relation, we describe such a wave as being *linearly damped* (see Sect. 4.2.1). Likewise, if $\gamma >0$, the wave amplitude increases exponentially with time and the wave is *linearly unstable* (see Sect. 6).

Neglecting any background electric field $\mathbf{E}_0$, we rewrite the electric and magnetic fields according to Eq. (135) as

138 $$\begin{aligned} \mathbf{E}(\mathbf{x},t)=\delta \mathbf{E}(\mathbf{x},t)=\mathrm {Re}\left[ \mathbf{E}(\mathbf{k},\omega )\exp \left( i\mathbf{k}\cdot \mathbf{x}-i\omega t\right) \right] \end{aligned}$$

and

139 $$\begin{aligned} \mathbf{B}(\mathbf{x},t)=\mathbf{B}_0+\delta \mathbf{B}(\mathbf{x},t)=\mathbf{B}_0+\mathrm {Re}\left[ \mathbf{B}(\mathbf{k},\omega )\exp \left( i\mathbf{k}\cdot \mathbf{x}-i\omega t\right) \right] , \end{aligned}$$

using the complex Fourier amplitudes $\mathbf{E}(\mathbf{k},\omega )$ and $\mathbf{B}(\mathbf{k},\omega )$. In the following, we write the Fourier amplitudes without their arguments $(\mathbf{k},\omega )$ and assume that $|\delta \mathbf{B}|\ll |\mathbf{B}_0|$. Substituting Eqs. (138) and (139) into Maxwell’s equations  (21) through (24), we find in Fourier space

140 $$\begin{aligned}&\mathbf{k}\cdot \mathbf{E}=-\,4\pi i \rho _{\mathrm {c}}, \end{aligned}$$

141 $$\begin{aligned}&\mathbf{k}\cdot \mathbf{B}=0, \end{aligned}$$

142 $$\begin{aligned}&\mathbf{k}\times \mathbf{E}-\frac{\omega }{c}\mathbf{B}=0, \end{aligned}$$

and

143 $$\begin{aligned} \mathbf{k}\times \mathbf{B}+\frac{\omega }{c}\mathbf{E}=-\frac{4\pi i}{c}\mathbf{j}, \end{aligned}$$

where

144 $$\begin{aligned} \rho _{\mathrm {c}}=\sum \limits _j \rho _{\mathrm {c}j}=\sum \limits _j q_jn_j \end{aligned}$$

is the charge density and

145 $$\begin{aligned} \mathbf{j}=\sum \limits _j \mathbf{j}_j \end{aligned}$$

is the current density. In Eqs. (144) and (145), the sums are carried over all particle species *j* in the plasma. The left-hand sides of Eqs. (140) through (143) represent the interactions between the electric and magnetic fields, while the right-hand sides represent the self-consistent effects of the particles on the fields.

We define the *plasma susceptibility tensor* $\varvec{\chi }_j$ of species *j* through

146 $$\begin{aligned} \varvec{\chi }_j\cdot \mathbf{E}\equiv \frac{4\pi i}{\omega }\mathbf{j}_j \end{aligned}$$

and the *dielectric tensor* $\varvec{\epsilon }$ as

147 $$\begin{aligned} \varvec{\epsilon }\equiv \mathbf{1} +\sum \limits _j \varvec{\chi }_j. \end{aligned}$$

The dielectric tensor is additive in the contributions from each plasma species *j* and reflects the interaction between fields and particles. With these definitions, we find

148 $$\begin{aligned} \varvec{\epsilon }\cdot \mathbf{E}=\mathbf{E}+\frac{4\pi i}{\omega }\mathbf{j} \end{aligned}$$

and, by using Eq. (143),

149 $$\begin{aligned} \mathbf{k}\times \mathbf{B}+\frac{\omega }{c}\varvec{\epsilon }\cdot \mathbf{E}=0. \end{aligned}$$

Combining Eq. (142) with Eq. (149) leads to the *wave equation*:

150 $$\begin{aligned} \mathbf{n} \times \left( \mathbf{n} \times \mathbf{E}\right) +\varvec{\epsilon }\cdot \mathbf{E} =\mathbf{{\mathcal {D}}}\cdot \mathbf{E}=0, \end{aligned}$$

where $\mathbf{n}\equiv \mathbf{k}c/\omega$ is the *refractive index* and

151 $$\begin{aligned} \mathbf{{\mathcal {D}}}\equiv \begin{pmatrix} \epsilon _{xx}-n_z^2 &{}\quad \epsilon _{xy} &{} \quad \epsilon _{xz}+n_xn_z \\ \epsilon _{yx} &{}\quad \epsilon _{yy}-n_x^2-n_z^2 &{} \quad \epsilon _{yz} \\ \epsilon _{zx}+n_zn_x &{}\quad \epsilon _{zy} &{} \quad \epsilon _{zz}-n_x^2 \end{pmatrix} \end{aligned}$$

is the *dispersion tensor*. The *phase velocity* of a solution is given by $\omega \mathbf{k}/k^2$. Non-trivial solutions to the wave equation fulfill

152 $$\begin{aligned} \mathrm {det}\,\left[ \mathbf{{\mathcal {D}}}(\mathbf{k},\omega )\right] =0, \end{aligned}$$

which is the mathematical dispersion relation. The identification of plasma waves then involves the calculation of a proper dielectric tensor for the plasma conditions at hand as well as the derivation of the roots of Eq. (152).

If the calculation of ${{\varvec{\epsilon }}}$ is based on the linearized Vlasov equation (Gary 1993), Eq. (152) leads to the full *hot-plasma dispersion relation*, which is a standard-tool in the calculation of plasma waves (Rönnmark 1982; Klein and Howes 2015; Verscharen and Chandran 2018; Verscharen et al. 2018). In this model, Eq. (20) is linearized for each plasma species *j* to first order in $\delta f_j$, under the assumption that $f_j=f_{0j}+\delta f_j$, as

153 $$\begin{aligned} \frac{\partial \delta f_j}{\partial t}+ \mathbf{v}\cdot \frac{\partial \delta f_j}{\partial \mathbf{x}}+\varOmega _j \left( \mathbf{v}\times \hat{\mathbf{b}} \right) \cdot \frac{\partial \delta f_{j}}{\partial \mathbf{v}}=-\frac{q_j}{m_j}\left( \delta \mathbf{E}+\frac{1}{c}\mathbf{v}\times \delta \mathbf{B}\right) \cdot \frac{\partial f_{0j}}{\partial \mathbf{v}},\nonumber \\ \end{aligned}$$

where the left-hand side describes the change of $\delta f_j$ along the zeroth-order particle trajectory, $\varOmega _j$ is calculated based on the background magnetic-field magnitude $B_0$, and $\hat{\mathbf{b}}\equiv \mathbf{B}_0/B_0$. The resulting solutions for $\delta f_j$ from integration along the particle trajectories then define $\rho _c$ and $\mathbf{j}$ according to Eqs. (25) and (26). We refer to the textbooks by Melrose and McPhedran (1991), Stix (1992), and Gary (1993) for more details on the calculation of $\varvec{\epsilon }$.

In our discussion of wave modes in Sect. 4.3, we present analytical results for wave dispersion and polarization relations based on different models and in different limits, which we identify whenever necessary. Fluid models and kinetic models often lead to different predictions in the dispersion relation and polarization properties of linear waves (see, e.g., Verscharen et al. 2017; Wu et al. 2019). These differences result from differences in the models’ underlying assumptions (e.g., the closure of the hierarchy of moment equations; see Sect. 1.4.1). Furthermore, analytical calculations of the dispersion relation often rely on mathematical approximations in certain limits (e.g., taking $m_{\mathrm {e}}\rightarrow 0$ or $T_j\rightarrow 0$). Before we discuss the wave modes further, we describe damping and dissipation mechanisms in the following section.

### Damping and dissipation mechanisms

The damping and dissipation of plasma waves are important for the global behavior of the plasma because these processes transfer energy between the electromagnetic fields and the particles and are also candidates for the dissipation of turbulent plasma fluctuations in the solar wind (see Sect. 5).

For our discussion, we distinguish between *damping* as a reduction in the amplitude of field fluctuations (i.e., $\gamma <0$) and *dissipation* as an irreversible increase in entropy of a plasma species (i.e., $\mathrm {d}S_j>0$, where $S_j$ is the entropy of species *j*). Lastly, we define *heating* as an increase of the plasma’s thermal energy. In this section, we address three important damping and dissipation mechanisms for plasma waves: (1) *quasilinear diffusion* from Landau-resonant or cyclotron-resonant wave–particle interactions, (2) *nonlinear phase mixing*, and (3) *stochastic heating*. So long as the Boltzmann equation (19) is valid, dissipation in the sense of entropy generation can only occur through particle–particle collisions. Even if collisions are not frequent enough to bring the plasma distribution function into local thermodynamic equilibrium, phase-space structures in the velocity distribution function can become small enough that collisions lead to dissipation (cf Sect. 3.2). When we study the dissipation of “collisionless” plasma waves, we, therefore, assume that collisions only affect small-scale structures in the distribution function and investigate the processes that create these small-scale structures, which in turn generate entropy through collisions. We note that deviations of velocity distributions from local thermodynamic equilibrium (see Sects. 1.4.4, 1.4.5) can affect the polarizations, transport ratios, and damping rates of the plasma normal modes, as well as the heating mechanisms (Chandran et al. 2013; Kasper et al. 2013; Klein and Howes 2015; Tong et al. 2015; Kunz et al. 2018).

#### Quasilinear diffusion

Quasilinear diffusion describes the evolution of the distribution function as velocity-space diffusion that arises from the resonant interaction between waves and particles (Marsch 2006). Quasilinear theory assumes the presence of a superposition of non-interacting and randomly phased waves that are solutions to linear plasma-wave theory as described in Sect. 4.1. The force term in the Vlasov equation is then averaged over the gyro-phases of the unperturbed particle orbits so that a diffusion term for the background distribution $f_{0j}$ in $v_{\perp }$ and $v_{\parallel }$ results, independent of the gyro-phase of the particles. This process is quasilinear in the sense that the fluctuations are solutions to the linear dispersion relation (Sect. 4.1), which closes the system of equations, but the field amplitudes enter the equations quadratically. In quasilinear theory, the background distribution $f_{0j}$ evolves slowly compared to the timescale of the fluctuations $1/\omega _{\mathrm {r}}$. Under the assumption of small wave amplitudes and $|\gamma /\omega _{\mathrm {r}}|\ll 1$, quasilinear diffusion follows the equation (Shapiro and Shevchenko 1962; Kennel and Engelmann 1966; Rowlands et al. 1966; Stix 1992)

154 $$\begin{aligned} \frac{\partial f_{0j}}{\partial t}=\frac{q_j^2}{8\pi ^2m_j^2}\lim \limits _{V\rightarrow \infty } \frac{1}{V} \sum \limits _{n=-\,\infty }^{+\infty }\int \mathrm {d}^3k\frac{1}{v_{\perp }}{\hat{G}}v_{\perp }\delta \left( \omega _{\mathrm {r}}-k_{\parallel }v_{\parallel }-n\varOmega _j\right) \left| \psi _{n}\right| ^2{\hat{G}} f_{0j},\nonumber \\ \end{aligned}$$

where the pitch-angle operator is defined as

155 $$\begin{aligned} {\hat{G}}\equiv \left( 1-\frac{k_{\parallel }v_{\parallel }}{\omega _{\mathrm {r}}}\right) \frac{\partial }{\partial v_{\perp }}+\frac{k_{\parallel }v_{\perp }}{\omega _{\mathrm {r}}}\frac{\partial }{\partial v_{\parallel }}, \end{aligned}$$

and

156 $$\begin{aligned} \psi _n\equiv \frac{1}{\sqrt{2}}\left[ E_{\mathrm {r}}e^{i\phi }J_{n+1}(\sigma _j)+E_{\mathrm {l}}e^{-i\phi }J_{n-1}(\sigma _j)\right] +\frac{v_{\parallel }}{v_{\perp }}E_zJ_{n}(\sigma _j). \end{aligned}$$

We define the wavevector components perpendicular and parallel to the background magnetic field as $k_{\perp }$ and $k_{\parallel }$, respectively. The right-handed and left-handed components of the Fourier-transformed electric field are $E_{\mathrm {r}}\equiv \left( E_x-iE_y\right) /\sqrt{2}$ and $E_{\mathrm {l}}\equiv \left( E_x+iE_y\right) /\sqrt{2}$, respectively, $J_n$ is the *n*th order Bessel function of the first kind, $\sigma _j\equiv k_{\perp }v_{\perp }/\varOmega _j$, $\phi$ is the azimuthal angle of $\mathbf{k}$, and *V* is the spatial volume under consideration. Since Eq. (154) is a second-order differential equation in $v_{\perp }$ and $v_{\parallel }$, it indeed corresponds to a diffusion in velocity space. The $\delta$-function in Eq. (154) guarantees that the only particles that participate in the resonant interactions are those for which $v_{\parallel }$ is equal to the *resonance speed*:

157 $$\begin{aligned} v_{\mathrm {res}}\equiv \frac{\omega _{\mathrm {r}}-n\varOmega _j}{k_{\parallel }}. \end{aligned}$$

Due to the form of ${\hat{G}}$, the diffusive flux of particles is tangent to semicircles in the $v_{\parallel }{-}v_{\perp }$ plane defined by

158 $$\begin{aligned} \left( v_{\parallel }-\frac{\omega _{\mathrm {r}}}{k_{\parallel }}\right) ^2+v_{\perp }^2=\mathrm {constant} \end{aligned}$$

and directed from larger to smaller values of $f_{0j}$ (Verscharen and Chandran 2013). During the diffusion, the particles gain kinetic energy if ($v_{\perp }^2+v _{\parallel }^2$) increases and lose it if this quantity decreases. The energy gained or lost by the particles is taken from or given to the wave at the resonant $k_{\parallel }$ and $\omega _{\mathrm {r}}$ so that this wave’s amplitude changes. The $n=0$ term in the sum in Eq. (154) corresponds to * Landau damping* (1946) and *transit-time damping*, and the $n\ne 0$ terms correspond to *cyclotron damping*.

**Fig. 15:**
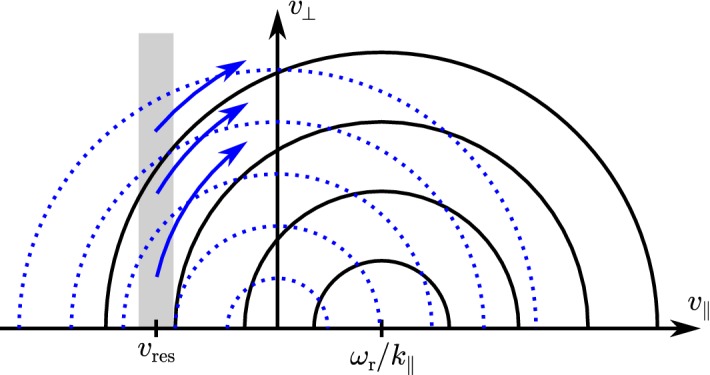
*Quasilinear diffusion* in the cyclotron-resonant damping of particles with $v_{\parallel }=v_{\mathrm {res}}<0$ (gray shaded area) with waves of parallel phase speed $\omega _{\mathrm {r}}/k_{\parallel }$. The blue dotted circles represent isocontours of the background distribution function $f_{0j}$. The diffusion paths (blue arrows) are locally tangential to circles around the point $(v_{\perp },v_{\parallel })=(0,\omega _{\mathrm {r}}/k_{\parallel })$ (black circles). In this example, the resonant particles gain kinetic energy, which corresponds to an increase in $(v_{\perp }^2+v_{\parallel }^2)$. This energy is removed from the waves at $\omega _{\mathrm {r}}$ and $k_{\parallel }$, which are thus damped

We illustrate the quasilinear diffusion process for a cyclotron-damped wave in Fig. 15. In this example, cyclotron-resonant particles with $v_{\parallel }=v_{\mathrm {res}}<0$ interact with waves with $\omega _{\mathrm {r}}$ and $k_{\parallel }$ and diffuse in velocity space. The cyclotron-resonant damping of left-handed waves propagating parallel to $\mathbf{B}_0$ exhibits these characteristics. We illustrate the case of quasilinear diffusion for a cyclotron-resonant instability in Fig. 20 in Sect. 6.

#### Entropy cascade and nonlinear phase mixing

Since dissipation, by definition, is irreversible, all dissipation processes cause entropy to increase. In a plasma with low collisionality, wave turbulence (see Sect. 5.2) is associated with fluctuations in entropy^13^ that cascade to small scales, where collisions have greater effects and ultimately dissipate these fluctuations. Applying Boltzmann’s *H*-theorem to Eq. (19), we obtain the entropy relation

159 $$\begin{aligned} \frac{\mathrm {d} S_j}{\mathrm {d}t}\!=\!\frac{\mathrm {d}}{\mathrm {d}t}\left( -\int \frac{\mathrm {d}^3\mathbf{r}}{V}\int \mathrm {d}^3\mathbf{v} \,f_j\ln f_j\right) \!=\!-\!\int \frac{\mathrm {d}^3\mathbf{r}}{V}\int \mathrm {d}^3\mathbf{v} \,\left( \frac{\delta f_j}{\delta t}\right) _{\mathrm {c}}\,\ln f_j,\qquad \end{aligned}$$

where $S_j$ is the entropy of species *j*, and *V* is the spatial volume under consideration. Equation (159) shows that entropy only increases in the presence of particle–particle collisions. We now separate $f_j$ into its equilibrium part $f_{0j}$ and its fluctuating part $\delta f_j$ as

160 $$\begin{aligned} f_j(\mathbf{x}, \mathbf{v},t)=f_{0j}(\mathbf{v})+\delta f_j(\mathbf{x},\mathbf{v},t). \end{aligned}$$

We assume that the collision frequency is of order $\omega _{\mathrm {r}}$,^14^ and $f_{0j}$ is a Maxwellian as in Eq. (59) with temperature $T_{0j}$. After averaging over the timescales greater than the typical fluctuation time $\sim 1/\omega _{\mathrm {r}}$ and summing over all species, we describe the evolution of the generalized energy through the energy equation with the help of the expression for the entropy from Eq. (159) as (Schekochihin et al. 2008)

161 $$\begin{aligned} \frac{\mathrm {d}W}{\mathrm {d}t}= & {} \frac{\mathrm {d}}{\mathrm {d}t}\int \frac{\mathrm {d}^3\mathbf{r}}{V}\left( \frac{E^2+B^2}{8\pi }+\sum \limits _j \int \mathrm {d}^3\mathbf{v} \frac{k_{\mathrm {B}}T_{0j}\delta f_j^2}{2f_{0j}}\right) \nonumber \\= & {} \epsilon +\int \frac{\mathrm {d}^3\mathbf{r}}{V}\sum \limits _j \int \mathrm {d}^3\mathbf{v}\frac{k_{\mathrm {B}}T_{0j}\delta f_j}{f_{0j}}\left( \frac{\delta f_j}{\delta t}\right) _{\mathrm {c}}, \end{aligned}$$

where *W* is the generalized energy and $\epsilon$ is the externally supplied power (e.g., through large-scale driving by shears or compressions).^15^

The *entropy cascade* constitutes the redistribution of generalized energy from electromagnetic fluctuations ($E^2+B^2$) to entropy fluctuations ($\delta f_j^2/f_{0j}$) according to Eq. (161). These fluctuations in entropy then cascade to smaller scales in velocity space through a combination of *linear* and *nonlinear phase mixing*. Linear phase mixing corresponds to Landau damping, which we describe in Sect. 4.2.1. The spread in parallel velocity of the particle distribution leads to a dependency of the Landau–resonant interactions between particles and the electric field on the particles’ parallel velocity.

Nonlinear phase mixing often serves as a faster mechanism of entropy cascade. A particle with a greater $v_{\perp }$ has a greater $\rho _j$ and thus experiences a slower $\mathbf{E}\times \mathbf{B}$ drift than a particle with smaller $v_{\perp }$ (Dorland and Hammett 1993). Two particles of the same species *j* but distinct perpendicular velocities $v_{\perp }$ and $v_{\perp }^{\prime }$ experience spatially decorrelated fluctuations in the electric and magnetic fields if the difference between the particles’ gyro-radii $v_{\perp }/|\varOmega _j|$ and $v_{\perp }^{\prime }/|\varOmega _j|$ is greater than the perpendicular correlation length $1/k_{\perp }$ of the field fluctuations (Schekochihin et al. 2008). In kinetic theory, this process leads to spatial perpendicular mixing of ion distributions with different gyro-centers and hence to the creation of small-scale structure in the gyro-center distribution. Small-scale structure in the fields in physical space thus leads to small-scale structure in the distribution function in velocity space perpendicular to $v_{\perp }$ as the result of this nonlinear phase mixing (Tatsuno et al. 2009; Bañón Navarro et al. 2011; Kawamori 2013; Navarro et al. 2016; Cerri et al. 2018). Once these velocity-space structures are small enough, collisions can efficiently smooth them—see Eq. (106) and the associated discussion—and thereby increase entropy and the perpendicular temperature of the ions.

#### Stochastic heating

Stochastic heating is a non-resonant energy-diffusion process. It arises from field fluctuations with spatial variations on the gyro-radius scale of the diffusing particles ($k_{\perp }\rho _j\sim 1$) and frequencies that are small compared to the gyro-frequency ($\omega _{\mathrm {r}}\ll |\varOmega _j|$) in a constant background magnetic field $\mathbf{B}_0$ (McChesney et al. 1987; Chen et al. 2001b; Johnson and Cheng 2001; Chaston et al. 2004; Fiksel et al. 2009).

**Fig. 16:**
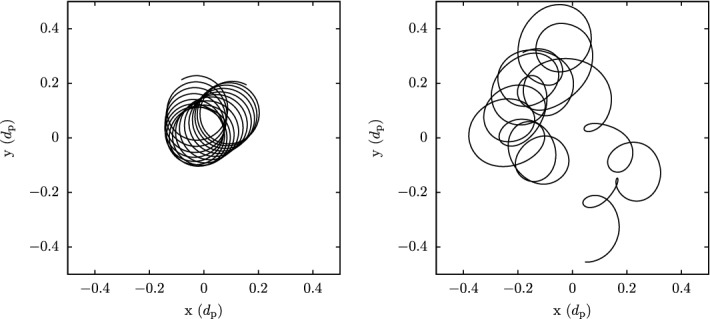
Trajectories of test particles in the plane perpendicular to $\mathbf{B}_0$. We use a setup similar to the kinetic-Alfvén-wave (KAW) simulations of stochastic heating described by Chandran et al. (2010). In the left panel, we show solutions for a thermal-proton trajectory when the amplitude of the Alfvénic fluctuations at $k_{\perp }\rho _{\mathrm {p}}\approx 1$ is small. The proton drifts due to the large-scale Alfvénic fluctuations, but its gyro-motion is still circular to first order. In the right panel, we show the same solutions but with an amplitude of the gyro-scale KAW fluctuations that is by a factor of five greater than in the left panel. The gyro-motion is strongly perturbed and becomes stochastic, creating the conditions for *stochastic heating*

If these fluctuations are low in amplitude, they induce only small perturbations in the particles’ otherwise circular orbits. With increasing amplitude, however, the fluctuations increasingly distort the gyro-orbits. If the amplitude of the gyro-scale fluctuations is so large that the orbits become stochastic in the plane perpendicular to $\mathbf{B}_0$, particles experience stochastic increases and decreases in their kinetic energy due to the fluctuations’ electric fields. Consequently, the particles diffuse in $v_{\perp }^2$, which corresponds to perpendicular heating (Chandran et al. 2010; Klein and Chandran 2016). This process is consistent with observations of solar-wind protons (Bourouaine and Chandran 2013; Martinović et al. 2019) and minor-ion temperatures and drifts (Chandran 2010; Wang et al. 2011; Chandran et al. 2013).

Figure 16 shows the orbits of two thermal protons in test-particle simulations of stochastic heating based on a superposition of randomly-phased kinetic Alfvén waves (KAWs; see Sect. 4.3.2). If the amplitude of the gyro-scale fluctuations is small (left panel), the magnetic moment is conserved and the particle trajectory corresponds to a drifting quasi-circular motion. If the amplitude of the gyro-scale fluctuations is large (right panel), the magnetic moment is no longer conserved. As a result, the particle’s trajectory becomes stochastic, which corresponds to stochastic heating through the waves’ electric fields.

The mechanisms of stochastic proton heating are different in the low-$\beta _{\mathrm {p}}$ regime and in the high-$\beta _{\mathrm {p}}$ regime. In plasmas with low $\beta _{\mathrm {p}}$, the proton orbits become stochastic mainly due to spatial variations in the electrostatic potential, and the protons primarily gain energy from the slow temporal variations in the electrostatic potential associated with the fluctuations (Chandran et al. 2010). In plasmas with high $\beta _{\mathrm {p}}$, the proton orbits become stochastic mainly due to spatial variations in the magnetic field, and the protons primarily gain energy from the solenoidal component of the electric field (Hoppock et al. 2018). Despite these differences, stochastic heating remains a universal candidate process to explain ion heating in the direction perpendicular to $\mathbf{B}_0$ in weakly collisional plasmas.

### Wave types in the solar wind

In this section, we discuss large-scale *Alfvén waves*, *kinetic Alfvén waves*, *Alfvén/ion-cyclotron waves*, *slow modes*, and *fast modes*, which are the most important wave types for the multi-scale dynamics of the solar wind. We note that the nomenclature of wave types is not universal and that different names are commonly used for waves of the same type depending on their location in wavevector space (e.g., TenBarge et al. 2012, Fig. 1).

#### Large-scale Alfvén waves

Alfvén waves are electromagnetic plasma waves for which magnetic tension serves as the restoring force (Alfvén 1942, 1943). To first order, these waves are non-compressive. At large scales (i.e., $kd_{\mathrm {p}}\ll 1$ and $k\rho _{\mathrm {p}}\ll 1$), Alfvén waves obey the linear dispersion relation

162 $$\begin{aligned} \omega = \pm \, |k_{\parallel }|v_{\mathrm {A}}^{*}, \end{aligned}$$

where the upper (lower) sign corresponds to propagation parallel (anti-parallel) to $\mathbf{B}_0$, and $v_{\mathrm {A}}^{*}\equiv B_0/\sqrt{4\pi \rho }$ is the MHD Alfvén speed. The group-velocity vector is parallel or anti-parallel to $\mathbf{B}_0$, and large-scale Alfvén waves are only weakly damped in a plasma with Maxwellian distribution functions. The fluctuating magnetic-field vector $\delta \mathbf{B}$ is perpendicular to $\mathbf{k}$ and $\mathbf{B}_0$. Alfvén waves are characterized by negligible fluctuations in $n_j$ (i.e., they are non-compressive) and $B\equiv |\mathbf{B}|$, but an (anti-)correlation between velocity fluctuations $\delta \mathbf{U}_j$ and magnetic-field fluctuations $\delta \mathbf{B}$. In the MHD approximation, this polarization property is given by

163 $$\begin{aligned} \frac{\delta \mathbf{U}}{v_{\mathrm {A}}^{*}}=\mp \frac{\delta \mathbf{B}}{B_0}. \end{aligned}$$

**Fig. 17:**
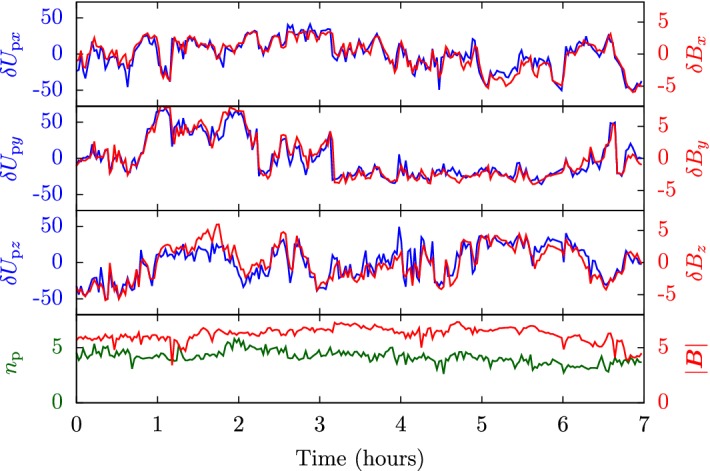
Alfvénic correlations between $\delta \mathbf{U}_{\mathrm {p}}$ and $\delta \mathbf{B}$. We show data from the Wind spacecraft’s SWE and MFI instruments starting at 18:01:59 on 2018-05-06 for a total duration of 7 h. The top three panels show the three components of the vector velocity (km/s; blue) and magnetic-field (nT; red) fluctuations. The vector components are positively correlated in this example. The bottom panel shows that the density ($\hbox {cm}^{-3}$; green) and the absolute value of the magnetic field (nT; red) stay approximately constant

In the solar wind, the center-of-mass frame, in which we define $\omega$ and $\mathbf{k}$, is dominated by the proton flow so that $\mathbf{U}\approx \mathbf{U}_{\mathrm {p}}$ and $\rho \approx n_{\mathrm {p}}m_{\mathrm {p}}$. Therefore, Eq. (163) is approximately $\delta \mathbf{U}_{\mathrm {p}}/v_{\mathrm {Ap}}\approx \mp \, \delta \mathbf{B}/B_0$. Observations of the vector components of the plasma velocity and the magnetic field in the solar wind often exhibit this polarization (Unti and Neugebauer 1968; Belcher et al. 1969; Belcher and Davis 1971; Bruno et al. 1985; Velli and Pruneti 1997; Chandran et al. 2009; Boldyrev and Perez 2012; He et al. 2012b, a; Podesta and TenBarge 2012), and we illustrate one such example in Fig. 17.

In fact, since this polarization characterizes the majority of the solar wind’s large-scale fluctuations, its large-scale turbulence is believed to be Alfvén-wave-like turbulence (see Sect. 5.2). At large scales, the amplitudes of the Alfvénic fluctuations in the solar wind are often so large that their behavior becomes nonlinear. Their polarization fulfills $B=\mathrm {constant}$, while the magnetic-field and velocity vectors often show a spherical or arc-like polarization (Tsurutani et al. 1994; Riley et al. 1996; Vasquez and Hollweg 1996). Although Alfvén waves predominantly occur in the fast solar wind, D’Amicis and Bruno (2015) identify a type of slow wind that also carries large-amplitude Alfvén waves and shows many other characteristics usually associated with fast wind (D’Amicis et al. 2019).

We note that left-circularly polarized and parallel-propagating Alfvén waves are a solution of the full nonlinear MHD and multi-fluid equations (Marsch and Verscharen 2011). At large scales, these waves follow a polarization relation that follows directly from the multi-fluid equations:

164 $$\begin{aligned} \frac{\delta \mathbf{U}_{j}}{v_{\mathrm {A}}^{*}}=\mp \left( 1 \mp \frac{U_{\parallel j}}{v_{\mathrm {A}}^{*}}\right) \frac{\delta \mathbf{B}}{B_0}, \end{aligned}$$

where the upper and lower signs describe the propagation direction as in Eq. (162). Equation (164) shows that a particle species with $U_{\parallel j}\approx v_{\mathrm {A}}^{*}$ does not participate in the bulk-velocity polarization motion associated with parallel-propagating large-scale Alfvén waves: in the reference frame of these particles, the wave has no electric field. Observations confirm that $\alpha$-particles (see Sect. 1.4.4) with $U_{\parallel \alpha }\approx v_{\mathrm {A}}^{*}$ exhibit $\delta \mathbf{U}_{\alpha }\approx 0$, which is an effect known as *surfing* $\alpha$*-particles* (Marsch et al. 1982a; Goldstein et al. 1995; Matteini et al. 2015b).

There are two extensions of the Alfvén wave to smaller scales: the kinetic Alfvén wave (KAW) at $k_{\perp }\rho _{\mathrm {p}} \gtrsim 1$ and $k_{\perp }\gg k_{\parallel }$, and the Alfvén/ion-cyclotron (A/IC) wave at $k_{\parallel }d_{\mathrm {p}}\gtrsim 1$ and $k_{\perp }\ll k_{\parallel }$. Although KAWs and A/IC waves belong to the Alfvén-wave family (Andre 1985; Yoon and Fang 2008; Klein and Howes 2015), we discuss them separately in the following two sections due to their great importance for the physics of the solar wind.

#### Kinetic Alfvén waves

Kinetic Alfvén waves (KAWs) are the short-wavelength extension of the Alfvén-wave branch for $k_{\perp }\gg k_{\parallel }$. This type of wave has received much attention since large-scale turbulence in the solar wind is Alfvén-wave-like and supports a cascade with increasing anisotropy toward $k_{\perp }\gg k_{\parallel }$ (see Sect. 5.2). Thus, KAWs are the prime candidate for extending the Alfvénic cascade to small scales.

When $k_{\perp }\rho _{\mathrm {p}}\gtrsim 1$, finite-Larmor-radius effects modify the properties of the Alfvén wave. The linear KAW dispersion relation in the gyrokinetic limit with isotropic temperatures is given by (Howes et al. 2006)

165 $$\begin{aligned} \omega =\pm \frac{|k_{\parallel }|v_{\mathrm {Ap}}k_{\perp }\rho _{\mathrm {p}}}{\sqrt{\beta _{\mathrm {p}}+{\displaystyle \frac{2}{ 1+T_{\mathrm {e}}/T_{\mathrm {p}}}}}}. \end{aligned}$$

KAWs are electromagnetic, are elliptically right-hand polarized, and have a frequency $\ll \varOmega _{\mathrm {p}}$ in this limit. While large-scale Alfvén waves are non-compressive, KAWs exhibit fluctuations in the particle density $n_j$ and the magnetic-field strength *B*. Observations of polarization properties of proton-scale and sub-proton-scale fluctuations in the solar wind and other space plasmas often find an agreement with the predicted KAW polarization (Bale et al. 2005; Salem et al. 2012; Chen et al. 2013; Podesta 2013; Roberts et al. 2013; Klein et al. 2014b; Šafránková et al. 2019; Zhu et al. 2019).

The compressive behavior of KAWs introduces fluctuations in the parallel electric field, allowing KAWs to experience Landau damping (see Sect. 4.2.1). Hybrid fluid-gyrokinetic simulations suggest that KAW turbulence leads to preferential electron heating at low $\beta _{\mathrm {p}}$ and to preferential ion heating at high $\beta _{\mathrm {p}}$ (Kawazura et al. 2019). At low $\beta _{\mathrm {p}}$, thermal protons do not satisfy the Landau-resonance condition according to Eq. (157) with $n=0$. In this case, the KAW turbulence cascades to even smaller scales, ultimately leading to preferential electron heating through electron Landau damping and subsequent collisions. At the same time, nonlinear phase mixing of the ions (see Sect. 4.2.2) creates smaller structures in the ions’ $v_{\perp }$ distribution, which eventually dissipate via collisions and perpendicularly heat the ions. At high $\beta _{\mathrm {p}}$, KAWs efficiently dissipate through proton Landau damping and subsequent collisions, which result in preferential parallel proton heating (Quataert 1998; Leamon et al. 1999; Howes 2010; Plunk 2013; TenBarge et al. 2013; He et al. 2015; Told et al. 2015; Hughes et al. 2017; Howes et al. 2018). Under certain conditions, KAW turbulence approaches the local ion-cyclotron frequency in the plasma frame, at which point perpendicular ion heating through cyclotron-resonant processes (see Sect. 4.2.1) occurs (Arzamasskiy et al. 2019).

In their stochastic-heating model (see Sect. 4.2.3), Chandran et al. (2010) determine the proton heating rate for stochastic heating by KAWs in low-$\beta _{\mathrm {p}}$ plasma to be

166 $$\begin{aligned} Q_{\perp }=c_1\frac{\left( \delta v_{\rho }\right) ^3}{\rho _{\mathrm {p}}}\exp \left( -\frac{c_2}{{\bar{\epsilon }}}\right) , \end{aligned}$$

where the empirical factors $c_1$ and $c_2$ are constants, $\delta v_{\rho }$ is the amplitude of the gyro-scale fluctuations in the $\mathbf{E}\times \mathbf{B}$ velocity, and ${\bar{\epsilon }}\equiv \delta v_{\rho }/w_{\perp \mathrm {p}}$. Test-particle simulations using plasma parameters consistent with low-$\beta _{\mathrm {p}}$ solar-wind streams suggest that $c_1\approx 0.75$ and $c_2\approx 0.34$ (Chandran et al. 2010), while reduced MHD simulations suggest larger values for $c_1$ and smaller values for $c_2$ (Xia et al. 2013).

In intermediate- to high-$\beta _{\mathrm {p}}$ plasma ($1\lesssim \beta _{\mathrm {p}}\lesssim 30$), the stochastic KAW proton heating rate is given by (Hoppock et al. 2018)

167 $$\begin{aligned} Q_{\perp }=\sigma _1\frac{\left( \delta v_{\rho }\right) ^3}{\rho _{\mathrm {p}}}\sqrt{\beta _{\mathrm {p}}}\exp \left( -\frac{\sigma _2}{{\bar{\delta }}}\right) , \end{aligned}$$

where $\sigma _1$ and $\sigma _2$ are constants, ${\bar{\delta }}\equiv \delta B_{\rho }/B_0$, and $\delta B_{\rho }$ is the amplitude of gyro-scale fluctuations in the magnetic field. Test-particle simulations suggest that $\sigma _1=5$ and $\sigma _2=0.21$.^16^

#### Alfvén/ion-cyclotron waves

Alfvén/ion-cyclotron (A/IC) waves are the short-wavelength extension of the Alfvén-wave branch for $k_{\parallel }\gg k_{\perp }$. The anisotropic Alfvénic turbulent cascade on its own cannot generate A/IC waves. However, A/IC waves have received considerable attention due to their ability to heat ions preferentially in the direction perpendicular to $\mathbf{B}_0$ through cyclotron resonance (see Sect. 4.2.1; Dusenbery and Hollweg 1981; Isenberg and Hollweg 1983; Gomberoff and Elgueta 1991; Hollweg 1999; Araneda et al. 2009; Rudakov et al. 2012).

The linear dispersion relation for quasi-parallel A/IC waves in the cold-plasma limit (i.e., $\beta _j\rightarrow 0$) is given by (Verscharen 2012)

168 $$\begin{aligned} \frac{\omega _{\mathrm {r}}}{\varOmega _{\mathrm {p}}}=\pm \frac{k^2d_{\mathrm {p}}^2}{2}\left( \sqrt{1+\frac{4}{k^2d_{\mathrm {p}}^2}}-1\right) . \end{aligned}$$

In this regime, the A/IC wave is also known as the *L-mode*. The frequency is always less than $\varOmega _{\mathrm {p}}$, and the quasi-parallel A/IC wave is almost fully left-circularly polarized—the same sense of rotation as the cyclotron motion of positively charged particles. This polarization accounts for the frequency cutoff at the proton cyclotron frequency, above which plasmas are opaque to A/IC waves. For finite-temperature plasmas, $\omega _{\mathrm {r}}$ asymptotes to an even smaller value than $\varOmega _{\mathrm {p}}$ since, with increasing temperature, an increasing number of particles resonate with the Doppler-shifted wave frequency in their reference frame.

The amplitudes of the perpendicular components of the fluctuating proton and electron bulk velocities are equal in the limit of $k\rightarrow 0$. The amplitude of the perpendicular proton bulk velocity then increases as $\omega _{\mathrm {r}}\rightarrow \varOmega _{\mathrm {p}}$, while the amplitude of the perpendicular electron bulk velocity remains approximately constant. Therefore, the proton contribution to the polarization current increases with $\omega _{\mathrm {r}}$, until the protons carry most of the current.

The inherent ambiguities of single-spacecraft measurements (see Sect. 2.6) complicate the identification of A/IC waves within background solar-wind turbulence. However, *A/IC-storms* have been observed as enhancements in the magnetic-field power spectrum at $\omega _{\mathrm {r}}\lesssim \varOmega _{\mathrm {p}}$ with predominantly left-handed polarization (Jian et al. 2009, 2010; He et al. 2011; Jian et al. 2014; Boardsen et al. 2015; Wicks et al. 2016).

A/IC waves damp on particles that fulfill the cyclotron-resonance condition according to Eq. (157) in Sect. 4.2.1 with $n=+\,1$,

169 $$\begin{aligned} \omega _{\mathrm {r}}=k_{\parallel } v_{\parallel } + \varOmega _{\mathrm {p}}. \end{aligned}$$

This effect heats ions very efficiently in the perpendicular direction. More specifically, the quasilinear pitch-angle diffusion through the $n=+\,1$ resonance creates a characteristic *plateau* along pitch-angle gradients, which has often been observed in the fast solar wind (Cranmer 2001; Isenberg 2001; Marsch and Tu 2001; Tu and Marsch 2001; Hollweg and Isenberg 2002; Gary et al. 2005; Kasper et al. 2013; Cranmer 2014; Woodham et al. 2018). These observations strongly support the A/IC-heating scenario, but difficulties remain in explaining the origin of these waves in the solar wind. Microinstabilities may play an important role in the generation of A/IC waves as we discuss in Sect. 6.

#### Slow modes

Although most solar-wind fluctuations are non-compressive, about 2% of the fluctuating power is in compressive modes in the inertial range (Chen 2016; Šafránková et al. 2019). Due to its polarization properties, the slow mode is a major candidate to explain these compressive fluctuations.

The linear dispersion relation of slow modes in the MHD limit is given by

170 $$\begin{aligned} \omega _{\mathrm {r}}=\pm \, kC_{-}, \end{aligned}$$

where

171 $$\begin{aligned} C_{\pm }\equiv v_{\mathrm {A}}^{*} \left[ \frac{1}{2}\left( 1+\frac{\kappa }{2}\beta _{\mathrm {p}}\right) \pm \frac{1}{2}\sqrt{\left( 1+\frac{\kappa }{2}\beta _{\mathrm {p}}\right) ^2-2\kappa \beta _{\mathrm {p}}\cos ^2\theta }\right] ^{1/2} \end{aligned}$$

is the fast (upper sign; see Sect. 4.3.5) and slow (lower sign) magnetosonic speed, $\kappa$ is the polytropic index, and $\theta$ is the angle between $\mathbf{k}$ and $\mathbf{B}_0$. Oblique MHD slow modes at $\beta _{\mathrm {p}}<2/\kappa$ are characterized by an anti-correlation between fluctuations in density $\delta n_{j}$ and magnetic-field strength $\delta |\mathbf{B}|$. In this limit, the mode is largely acoustic in nature, and the mode’s velocity perturbation is closely aligned with $\mathbf{B}_0$. In the high-$\beta _{\mathrm {p}}$ limit, the MHD slow mode is largely tensional in nature, and the mode’s velocity perturbation $\delta \mathbf{U}$ is predominantly (anti-)parallel to $\mathbf{B}_0$. In both of these limits of the MHD slow wave, the vector $\delta \mathbf{B}$ lies in the $\mathbf{k}{-}\mathbf{B}_0$ plane. In the limit of $\theta =0^{\circ }$, the MHD slow wave is either a pure acoustic wave with $\delta \mathbf{B}=0$ when $\beta _{\mathrm {p}}<2\kappa$ or degenerate with the Alfvén wave when $\beta _{\mathrm {p}}>2\kappa$. In the limit of $\theta =90^{\circ }$, the slow mode does not propagate.

Polarization properties are often more useful than phase speeds in defining the type of plasma wave. Therefore, we more generally define slow modes as the solutions to the dispersion relation that exhibit the anti-correlation between $\delta n_{j}$ and $\delta |\mathbf{B}|$ that characterizes the MHD slow mode’s low-$\beta _{\mathrm {p}}$ limit. In kinetic theory, two solutions exhibit this anti-correlation.^17^ We consequently identify both of them with the *kinetic slow mode* (Verscharen et al. 2017).

The first solution is the *ion-acoustic wave* (Narita and Marsch 2015), which obeys the linear dispersion relation

172 $$\begin{aligned} \omega _{\mathrm {r}}=\pm \, |k_{\parallel }|\sqrt{\frac{3k_{\mathrm {B}}T_{\parallel \mathrm {p}}+k_{\mathrm {B}}T_{\parallel \mathrm {e}}}{m_{\mathrm {p}}}} \end{aligned}$$

which can be obtained in the gyrokinetic limit (Verscharen et al. 2017). The phase speed of this wave is the ion-acoustic speed, which indicates that the parallel pressures of protons and electrons provide this mode’s restoring force, while the proton mass provides its inertial force. The protons behave like a one-dimensional adiabatic fluid since $\kappa _{\mathrm {p}}=3$, while the electrons behave like an isothermal fluid since $\kappa _{\mathrm {e}}=1$, where $\kappa _j$ is the polytropic index of species *j*.

The second type of kinetic slow mode is the *non-propagating mode*,^18^ which obeys the linear dispersion relation

173 $$\begin{aligned} \omega _{\mathrm {r}}=0. \end{aligned}$$

If any plasma species has a sufficiently strong temperature anisotropy with $T_{\perp j}>T_{\parallel j}$, the non-propagating mode can become unstable and then gives rise to the mirror-mode instability (see Sect. 6.1.1).

**Fig. 18:**
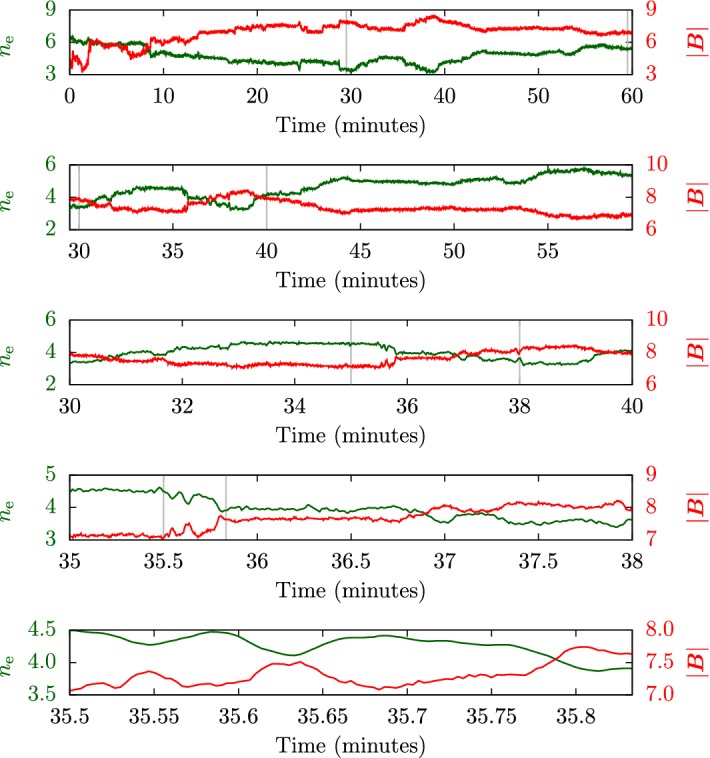
Time series of $n_{\mathrm {e}}$ ($\hbox {cm}^{-3}$; green) and $|\mathbf{B}|$ (nT; red) in the solar wind on multiple scales, each of which has fluctuations that clearly exhibit the anti-correlation between $\delta n_{\mathrm {e}}$ and $\delta |\mathbf{B}|$ that characterizes slow waves. These panels show data from the Cluster EFW and FGM instruments measured for 1 h starting at 22:30:00 on 2001-04-05. Following the technique by Yao et al. (2011), we show from top to bottom decreasing interval lengths. The gray lines in each plot indicate the start and end points of the interval shown in the plot immediately below it. We use a running average to filter the spacecraft spin tones from the data

The anti-correlation of $\delta n_j$ and $\delta |\mathbf{B}|$, which defines slow modes, is frequently observed in the solar wind (Yao et al. 2011; Kellogg and Horbury 2005; Chen et al. 2012b; Howes et al. 2012; Klein et al. 2012; Roberts et al. 2017; Yang et al. 2017a; Roberts et al. 2018). Figure 18 shows a period of solar-wind measurements that exemplify this anti-correlation over a wide range of scales.

Ion-acoustic waves mainly damp through Landau damping (Barnes 1966). Since the mode’s phase speed is of order the proton thermal speed (unless $T_{\parallel \mathrm {e}}\gg T_{\parallel \mathrm {p}}$), the ion-acoustic mode predominantly heats ions in the field-parallel direction. We note that the damping rate of slow modes is significant even at scales $\gg d_{\mathrm {p}}$. On this basis, slow modes have at times been rejected as candidates for the compressive fluctuations in the solar wind. Nevertheless, at very large angles between $\mathbf{k}$ and $\mathbf{B}_0$, the damping rate decreases significantly, and the ion-acoustic wave and the MHD slow wave no longer propagate. Instead, they become non-propagating structures that exhibit pressure balance,

174 $$\begin{aligned} P_{\mathrm {tot}}\equiv P+\frac{B^2}{8\pi }=\mathrm {constant}. \end{aligned}$$

These *pressure-balanced structures* have been observed often and across many scales both in the solar wind and in plasma simulations (Burlaga and Ogilvie 1970; Marsch and Tu 1990b, 1993; Tu and Marsch 1994; Bavassano et al. 2004; Verscharen et al. 2012a; Yao et al. 2013a, b). A recent study suggests that slow modes also play an important role in how low-frequency, low-$\beta _j$ plasma turbulence partitions heating between ions and electrons (Schekochihin et al. 2019).

#### Fast modes

Fast modes are another type of compressive fluctuation, although they are non-compressive in parallel propagation. Their linear dispersion relation in the MHD approximation is given by

175 $$\begin{aligned} \omega _{\mathrm {r}}=\pm \, kC_+, \end{aligned}$$

where $C_+$ is the fast magnetosonic speed according to Eq. (171). Oblique MHD fast modes at $\beta _{\mathrm {p}}<2/\kappa$ are characterized by a positive correlation between fluctuations in density $\delta n_{j}$ and magnetic-field strength $\delta |\mathbf{B}|$. In this limit, the mode’s restoring force is a combination of the total-pressure-gradient force and the magnetic-tension force, and its velocity perturbation $\delta \mathbf{U}$ lies in the $\mathbf{k}{-}\mathbf{B}_0$ plane. In the high-$\beta _{\mathrm {p}}$ limit, the MHD fast mode is largely acoustic in nature, and the mode’s velocity perturbation $\delta \mathbf{U}$ is mainly parallel to $\mathbf{k}$. In the limit of $\theta =0^{\circ }$, the MHD fast wave is either degenerate with the Alfvén wave when $\beta _{\mathrm {p}}<2\kappa$ or a purely acoustic wave with its velocity perturbation $\delta \mathbf{U}$ parallel to $\mathbf{k}$ when $\beta _{\mathrm {p}}>2\kappa$. In the limit of $\theta =90^{\circ }$, the MHD fast mode is a magnetoacoustic pressure wave. In the MHD fast wave, the vector $\delta \mathbf{B}$ lies in the $\mathbf{k}{-}\mathbf{B}_0$ plane. Analogous to the case of generalized slow modes, we define fast modes as the solutions to the linear dispersion relation that exhibit a characteristic positive correlation between $\delta n_j$ and $\delta |\mathbf{B}|$ known from the low-$\beta _{\mathrm {p}}$ limit of the MHD fast mode.

On smaller scales, the fast-mode family includes the *whistler mode*, the *lower-hybrid mode*, and the *kinetic magnetosonic mode*. We refer to all modes of this family as *fast-magnetosonic/whistler (FM/W) waves*. In the limit $k d_{\mathrm {e}}\ll 1$ in a cold plasma with quasi-parallel direction of propagation, the linear FM/W-wave dispersion relation is approximately given by

176 $$\begin{aligned} \frac{\omega _{\mathrm {r}}}{\varOmega _{\mathrm {p}}}=\pm \frac{k^2d_{\mathrm {p}}^2}{2}\left( \sqrt{1+\frac{4}{k^2d_{\mathrm {p}}^2}}+1\right) , \end{aligned}$$

which connects to the Alfvén-wave branch at small *k* as in Eq. (168). The quasi-parallel FM/W wave is also known as the *R-mode*. In the limit $kd_{\mathrm {p}}\gg 1$ and allowing for oblique propagation with $\cos ^2 \theta \gtrsim m_{\mathrm {e}}/m_{\mathrm {p}}$, the cold-plasma FM/W-wave dispersion relation can be approximated by

177 $$\begin{aligned} \frac{\omega _{\mathrm {r}}}{|\varOmega _{\mathrm {e}}|}\approx \pm \frac{k|k_{\parallel }|d_{\mathrm {e}}^2}{1+k^2d_{\mathrm {e}}^2}. \end{aligned}$$

In the limit $k\rightarrow \infty$, this dispersion relation asymptotes toward $\sim |\varOmega _{\mathrm {e}}|\cos \theta$. In this regime, the FM/W wave is known as the whistler wave. The amplitudes of the perpendicular components of the fluctuating proton and electron bulk velocities are equal in the limit of $k\rightarrow 0$. The amplitude of the fluctuations in the perpendicular electron bulk velocity then increases as $\omega _{\mathrm {r}}\rightarrow |\varOmega _{\mathrm {e}}|$ while the amplitude of the fluctuations in the perpendicular proton bulk velocity decreases until the proton bulk velocity is almost zero. Therefore, the electron contribution to the polarization current increases with $\omega _{\mathrm {r}}$ until the electrons carry most of the current. The electrons remain magnetized at these frequencies, while the protons are unmagnetized. The phase speed of whistler waves is proportional to *k*, so waves with a higher frequency travel faster than waves with a lower frequency. This strongly dispersive behavior of whistler waves is responsible for their name since they were first discovered as whistling sounds with decreasing pitch in radio measurements of ionospheric disturbances caused by lightning (Barkhausen 1919; Storey 1953).

In the highly-oblique limit ($\cos ^2 \theta \lesssim m_{\mathrm {e}}/m_{\mathrm {p}}$), the FM/W wave corresponds to the lower-hybrid wave. A useful approximation for its linear dispersion relation in the cold-plasma limit is (Verdon et al. 2009)

178 $$\begin{aligned} \frac{\omega _{\mathrm {r}}^2}{\omega _{\mathrm {LH}}^2}\approx \frac{1}{1+\omega _{\mathrm {e}}^2/k^2c^2}\left( 1+\frac{m_{\mathrm {p}}}{m_{\mathrm {e}}}\frac{\cos ^2\theta }{1+\omega _{\mathrm {pe}}^2/k^2c^2}\right) , \end{aligned}$$

where

179 $$\begin{aligned} \omega _{\mathrm {LH}}\equiv \frac{\omega _{\mathrm {pp}}}{\sqrt{1+\displaystyle \frac{\omega _{\mathrm {pe}}^2}{\varOmega _{\mathrm {e}}^2}}} \end{aligned}$$

is the *lower-hybrid frequency*. Under typical solar-wind conditions, $\beta _{\mathrm {p}}\gtrsim 10^{-3}$, and the lower-hybrid wave is very strongly Landau-damped. However, this mode may be driven unstable by certain electron configurations and thus account for some of the electrostatic noise observed in the solar wind (Marsch and Chang 1982; Lakhina 1985; Migliuolo 1985; McMillan and Cairns 2006).

Quasi-parallel FM/W waves are right-hand polarized—the same sense of rotation as the cyclotron motion of electrons. This polarization results in a frequency cutoff at the electron gyro-frequency. FM/W waves are almost undamped at ion scales ($kd_{\mathrm {e}}\ll 1$). When they reach the electron scales, they cyclotron-resonate with thermal electrons very efficiently through the $n=-\,1$ resonance (see Sect. 4.2.1). This leads to efficient perpendicular electron heating. Oblique FM/W modes can resonate with ions through other resonances, including the Landau resonance with $n=0$.

Quasi-perpendicular FM/W waves have been an alternative candidate to KAWs for explaining the observed solar-wind fluctuations at $k_{\perp }\rho _{\mathrm {p}}\gtrsim 1$ (Coroniti et al. 1982; He et al. 2012a; Sahraoui et al. 2012; Narita et al. 2016). However, their existence is unlikely to result from the large-scale Alfvénic cascade since this scenario would necessitate a transition from Alfvénic modes to fast modes at some point in the cascade. The solar wind only rarely exhibits pronounced time intervals with a positive correlation between $\delta n_j$ and $\delta |\mathbf{B}|$ at large scales (Klein et al. 2012). However, a number of observations of polarization properties of fluctuations reveal occasional consistency with the predictions for FM/W waves (Beinroth and Neubauer 1981; Marsch and Bourouaine 2011; Chang et al. 2014; Gary et al. 2016a; Narita et al. 2016). FM/W modes may be the result of a class of microinstabilities (see Sects. 6.1.1, 6.1.2) and thus may be important for the thermodynamics of the solar wind beyond the turbulent cascade.

## Plasma turbulence

After a brief introduction to the phenomenology of plasma turbulence in Sect. 5.1, we discuss the important concepts of wave turbulence in Sect. 5.2 and critical balance in Sect. 5.3. Section 5.4 closes our description of turbulence with a brief discussion of more advanced topics. There are many excellent textbooks and review articles on plasma turbulence (e.g., Tu and Marsch 1995; Bavassano 1996; Petrosyan et al. 2010; Bruno and Carbone 2013). We refer the reader to this literature for a deeper discussion of the topic.

### Phenomenology of plasma turbulence in the solar wind

Turbulence is a state of fluids in which their characteristic quantities such as their velocity or density fluctuate in an effectively unpredictable way.^19^ Fluids with low viscosity transition easily into a turbulent flow pattern. Turbulence is inherently a multi-scale phenomenon. Energy enters the system at large scales. Nonlinear interactions between fluctuations on comparable scales then transfer the energy to fluctuations on different scales with a net transfer of energy to smaller and smaller scales. This cascade of energy occurs through the interaction of neighboring eddies in the fluid that break up into smaller eddies. At the smallest scales, the fluctuations eventually dissipate into heat through collisions and raise the medium’s entropy. In a neutral fluid, the injection at large scales may represent a slow (compared to the characteristic time associated with the turbulent cascade) stirring mechanism. The dissipation is a consequence of the viscous interaction, which strengthens with decreasing scale. Turbulence in a plasma, however, is different from turbulence in a neutral fluid due to the additional, electromagnetic interactions and the presence of additional, non-viscous dissipation channels at the characteristic plasma scales ($\rho _j$, $d_j$, $\lambda _j$, etc.). The solar wind, due to its low collisionality, exemplifies such a turbulent plasma.

**Fig. 19:**
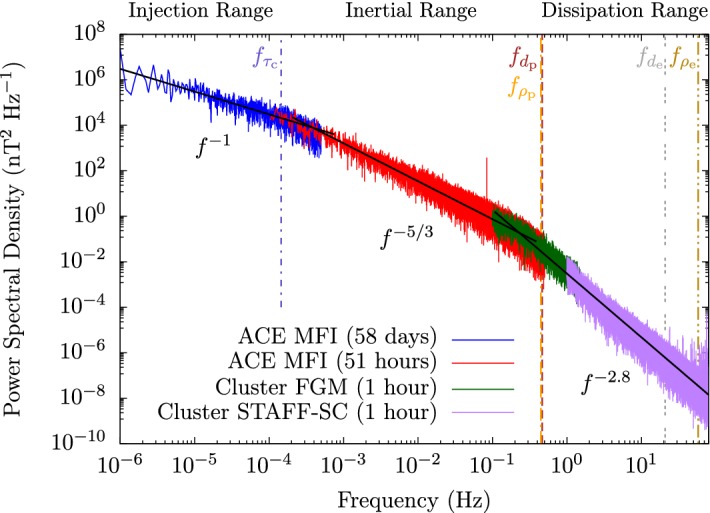
Power spectral density of magnetic-field fluctuations in the solar wind during a time interval with $\beta _{\mathrm {p}}\sim 1$. The black lines show power laws with the power indices ${-}$ 1, ${-}$ 5/3, and ${-}$ 2.8, which are characteristic of the injection, inertial, and dissipation ranges, respectively. The frequency is measured in the spacecraft reference frame. The average plasma parameters are $B=4.528\,\mathrm {nT}$, $n_{\mathrm {p}}=1.02\,\mathrm {cm}^{-3}$, $n_{\mathrm {e}}=1.12\,\mathrm {cm}^{-3}$, $T_{\mathrm {p}}=1.26\,\mathrm {MK}$, $T_{\mathrm {e}}=0.138\,\mathrm {MK}$, and $U_{\mathrm {p}}=658\,\mathrm {km/s}$. After Kiyani et al. (2015)

The multi-scale nature of turbulence leads to a broad power-law in the power spectral density of the fluctuating quantities. For fluid turbulence, a dimensional scale analysis shows that the power spectral density in the *inertial range*, which is the range of scales between the large *injection scales* and the small *dissipation scales*, follows a power law in wavenumber *k* (see also Fig. 19). Kolmogorov (1941a, b) estimates the power index of the power spectral density of the fluid velocity fluctuations by employing the following dimensional analysis. He identifies the dissipation rate with the constant rate of energy transfer $\epsilon$ in the inertial range under steady-state conditions. For an eddy of size $\ell$ and velocity difference $\delta U_{\ell }$ across its extent, the characteristic time to turn over is approximately $\tau _{\mathrm {nl}}\sim \ell /\delta U_{\ell }$. The transfer rate of energy density for this eddy, on the other hand, is related to the energy density ${\mathcal {E}}$ through $\epsilon \sim {\mathcal {E}}/\tau _{\mathrm {nl}}=\mathrm {constant}$, where ${\mathcal {E}}\sim \left( \delta U_{\ell }\right) ^2$. Combining these relations, we find ${\mathcal {E}}\sim \left( \epsilon \ell \right) ^{2/3}$. Relating scale and wavenumber through $\ell \sim 1/k$ and defining the power spectral density as $E(k)\sim {\mathcal {E}}/k$ then leads to

180 $$\begin{aligned} E(k)\sim \epsilon ^{2/3}k^{-5/3}. \end{aligned}$$

Such a power law in *k* is characteristic of turbulent fluids. Indeed, spectra of the solar wind’s magnetic field, which have been measured in progressively greater detail for decades, often exhibit this power law (Coleman 1968; Kiyani et al. 2015). We show an exemplar power spectrum of solar-wind magnetic fluctuations in frequency in Fig. 19, which spans almost eight orders of magnitude in frequency (for other examples, see Leamon et al. 1998; Alexandrova et al. 2009; Sahraoui et al. 2010b; Bruno et al. 2017). We use the same instruments and data intervals in January and February of 2007 as Kiyani et al. (2015) and compose a spectrum based on a direct fast Fourier analysis of a 58-day interval from ACE MFI, a 51-h interval from ACE MFI, a 1-h interval from Cluster 4 FGM, and the same 1-h interval from Cluster 4 STAFF-SC. These time intervals are nested: each interval lies within the next longer time interval.

When a single spacecraft measures a time series of a fluctuating quantity, it cannot distinguish between local temporal variations and variations due to the convection of spatial structures over the spacecraft with the solar-wind speed. Even purely spatial variations appear as temporal variations, so a power spectrum in frequency reflects the combined effects of temporal and spatial variations (Taylor 1938). More precisely, the Doppler shift connects the observed frequency $f_{\mathrm {sc}}$ of fluctuations in the spacecraft frame to the wavevector $\mathbf{k}$ and the frequency $f_{0}$ of the fluctuations in the plasma frame through

181 $$\begin{aligned} f_{\mathrm {sc}}=f_0+\frac{1}{2\pi }\mathbf{k}\cdot \varDelta \mathbf{U}, \end{aligned}$$

where $\varDelta \mathbf{U}$ is the velocity difference between the spacecraft frame and the plasma frame. For low-frequency fluctuations (i.e., $f_0\ll \mathbf{k}\cdot \varDelta \mathbf{U}$), *Taylor’s hypothesis* simplifies the Doppler-shift relationship in Eq. (181) to

182 $$\begin{aligned} f_{\mathrm {sc}}\approx \frac{1}{2\pi } \mathbf{k}\cdot \varDelta \mathbf{U}, \end{aligned}$$

which is often used in the analysis of solar-wind fluctuations (for a more detailed discussion of its applicability, see Howes et al. 2014b; Klein et al. 2014a, 2015; Bourouaine and Perez 2018). In Fig. 19, we use Taylor’s hypothesis to convert the convected frequencies associated with the scales $d_{j}$ and $\rho _j$ as $f_{d_j}\equiv U_{\mathrm {p}}/2 \pi d_j$ and $f_{\rho _j}\equiv U_{\mathrm {p}}/2 \pi \rho _j$, respectively, based on the average Cluster 4 FGM, CIS, and PEACE measurements during the 1-h time interval used in this analysis.

Figure 19 shows all three of the typical ranges observed in the solar wind. At the lowest frequencies ($f_{\mathrm {sc}}\lesssim 10^{-4}\,\mathrm {Hz}$), is the injection range, which follows a power law with $f_{\mathrm {sc}}^{-1}$. For comparison, we note that the expansion time of $\tau =2.4\,\mathrm {d}$ corresponds to a frequency of about $5\times 10^{-6}\,\mathrm {Hz}$, while the solar rotation period $\tau _{\mathrm {rot}}=25\,\mathrm {d}$ corresponds to a frequency of about $5\times 10^{-7}\,\mathrm {Hz}$ (see Sect. 1.1). The nature and origin of fluctuations in the injection range are not well understood (Matthaeus and Goldstein 1986; Verdini et al. 2012; Consolini et al. 2015). The fluctuations exhibit Alfvénic polarization properties (see Sect. 4.3.1) and $B\approx \mathrm {constant}$ (Matteini et al. 2018; Bruno et al. 2019).

At intermediate frequencies ($10^{-4}\,\mathrm {Hz}\lesssim f _{\mathrm {sc}}\lesssim 1\,\mathrm {Hz}$), the inertial range of magnetic fluctuations approximately follows a power law with $f_{\mathrm {sc}}^{-5/3}$, which roughly agrees with Kolmogorov’s theory according to Eq. (180). Fluctuations in other quantities, such as bulk velocity (Boldyrev et al. 2011) and density (Kellogg and Horbury 2005), have similar but not identical spectral indices compared to the magnetic fluctuations. The differences between the magnetic-field and velocity spectra are interpreted as resulting from significant residual energy being generated at large scales. At high frequencies ($f_{\mathrm {sc}}\sim 1\,\mathrm {Hz}$), the magnetic-field spectrum steepens again toward a power law approximately following $f_{\mathrm {sc}}^{-2.8}$, which may indicate the beginning of the *dissipation range*. The power index at small scales varies, however, and the origin of this break is still unclear. Recent work suggests that there is a further transition at the electron scales toward an even steeper slope of the spectrum (Alexandrova et al. 2009; Sahraoui et al. 2009). The e-folding de-correlation time of the 51-h time interval is $\tau _{\mathrm {c}}=18.3\,\mathrm {min}$, and we define $f_{\tau _{\mathrm {c}}}\equiv 1/ 2\pi \tau _{\mathrm {c}}$ as the spacecraft frequency associated with the e-folding de-correlation length. Like most properties of the solar wind, the fluctuations change with distance from the Sun. For instance, solar-wind expansion causes the overall level of fluctuation amplitudes to decrease with distance (Bavassano et al. 1982; Burlaga and Goldstein 1984). The power of the large-scale magnetic-field fluctuations beyond a few tens of $R_{\odot }$ decreases approximately $\propto r^{-3}$ as predicted by WKB theory (Belcher and Burchsted 1974; Hollweg 1974). Moreover, the positions of the spectral breakpoints vary with distance (Matthaeus and Goldstein 1982; Bavassano and Smith 1986; Roberts et al. 1987). The spacecraft-frame frequency $f_{\mathrm {b}1}$ of the breakpoint between the injection range and the inertial range decreases with distance *r* from the Sun as $f_{\mathrm {b}1}\propto r^{-1.5}$ (Bruno et al. 2009), while the frequency $f_{\mathrm {b}2}$ of the breakpoint between the inertial range and the dissipation range decreases as $f_{\mathrm {b}2}\propto r^{-1.09}$ (Bruno and Trenchi 2014).

The importance of damping and dissipation of plasma turbulence in the solar wind is underlined by the finding that the energy cascade rate through the inertial range in solar-wind turbulence (e.g., MacBride et al. 2008) is typically sufficient to explain the observed heating of the solar wind (see Sect. 1.4.6). These studies are based on the relationship found by Politano and Pouquet (1998), which estimates the energy transfer rate assuming isotropy, incompressibility, homogeneity, and equipartition between magnetic and kinetic energies. However, it is as yet unclear what underlying physics mechanisms heat the plasma through the damping and dissipation of the turbulent fluctuations.

### Wave turbulence and its composition

In order to understand the effects of solar-wind turbulence on the multi-scale evolution of the plasma, we must determine the nature of the fluctuations. Iroshnikov (1963) and Kraichnan (1965) suggest that *MHD turbulence* in a strongly magnetized medium is a manifestation of nonlinear collisions between counter-propagating Alfvén-wave packets. According to their statistically isotropic theory, the Alfvén-wave-collision mechanism leads to a power law of the magnetic-field spectrum with

183 $$\begin{aligned} E(k)\sim k^{-3/2} \end{aligned}$$

in the inertial range. This work introduced the framework of *wave turbulence* (see also Howes et al. 2014a) into plasma-turbulence research. Wave turbulence accounts for the fact that a plasma, unlike a neutral fluid, carries plasma waves as linear normal modes for the system (see Sect. 4.1). The linear response of the system still plays a role in the dynamics of the turbulence, even though the evolution of the turbulence is nonlinear. Therefore, fluctuations in wave turbulence retain certain characteristics of the plasma’s linear normal modes such as propagation and polarization properties. In the wave-turbulence framework, the identification of the nature of plasma turbulence is thus informed by the identification of the dominant wave modes of the turbulence. As a caveat to this picture, we note that nonlinear interactions may generate fluctuations that are not (linear) normal modes of the system as those described in Sect. 4.3. These driven modes may behave unexpectedly, and linear theory does not predict their properties.

There are two important timescales associated with fluctuations in wave turbulence: the *linear time* $\tau _{\mathrm {lin}}$ and the *nonlinear time* $\tau _{\mathrm {nl}}$. The linear time is associated with the evolution of the plasma’s dominant wave modes due to propagation along $\mathbf{B}_0$. It is related to the wave frequency through

184 $$\begin{aligned} \tau _{\mathrm {lin}}\sim \frac{1}{\omega _{{\mathrm {r}}}}. \end{aligned}$$

The nonlinear time is associated with the nonlinear interaction between the modes perpendicular to the field direction, which leads to the nonlinear cascade process. It is related to the perpendicular wavenumber $k_{\perp }$ and the perpendicular fluctuations in velocity $\delta U_{\perp }$ through

185 $$\begin{aligned} \tau _{\mathrm {nl}}\sim \frac{1}{k_{\perp }\,\delta U_{\perp }}. \end{aligned}$$

Turbulence is called *strong* when $\tau _{\mathrm {lin}} \gtrsim \tau _{\mathrm {nl}}$ and *weak* when $\tau _{\mathrm {lin}}\ll \tau _{\mathrm {nl}}$. Wave turbulence can exist in the strong and in the weak regime, and we emphasize that the terms *wave turbulence* and *weak turbulence* are not interchangeable.

In the weak-turbulence paradigm, the collision of two waves with frequencies $\omega _1$ and $\omega _2$ and with wavevectors $\mathbf{k}_1$ and $\mathbf{k}_2$ most efficiently leads to a resultant wave with frequency (Montgomery and Turner 1981; Shebalin et al. 1983; Montgomery and Matthaeus 1995)

186 $$\begin{aligned} \omega _3=\omega _1+\omega _2 \end{aligned}$$

and wavevector

187 $$\begin{aligned} \mathbf{k}_3=\mathbf{k}_1+\mathbf{k}_2. \end{aligned}$$

Assuming Alfvén waves with $\omega =\pm \, k_{\parallel }v_{\mathrm {A}}^{*}$ (see Sect. 4.3.1), where $k_{\parallel }\equiv \mathbf{k} \cdot \mathbf{B}_0/B_0$, these wave–wave resonances cannot feed an MHD Alfvén-wave triad with $\omega _3\ne 0$. Although $k_{\perp }$ can increase, these triads lead to a situation with $k_{\parallel }\rightarrow 0$, where $k_{\perp }\equiv |\mathbf{k}-k_{\parallel }\mathbf{B}_0/B_0|$. This weak-turbulence process plays an important role in the onset of plasma turbulence because it creates increasingly perpendicular wavevectors. Indeed, spacecraft observations show a strong wavevector anisotropy with $k_{\perp }\gg k_{\parallel }$ in the solar wind for the majority of turbulent fluctuations (Dasso et al. 2005; Hamilton et al. 2008; Tessein et al. 2009; MacBride et al. 2010; Wicks et al. 2010; Chen et al. 2011a; Ruiz et al. 2011; Chen et al. 2012a; Horbury et al. 2012; Oughton et al. 2015; Lacombe et al. 2017).

Indirect measurements of the *two-point correlation function*

188 $$\begin{aligned} R(\mathbf{r})\equiv \left\langle \mathbf{B}(\mathbf{x})\cdot \mathbf{B}(\mathbf{x}+\mathbf{r}) \right\rangle \end{aligned}$$

and the *magnetic helicity*

189 $$\begin{aligned} H\equiv \int \mathbf{A}\cdot \mathbf{B}\,\mathrm {d}^3\mathbf{x}, \end{aligned}$$

where $\langle \cdots \rangle$ indicates the average over many positions $\mathbf{x}$, and $\mathbf{A}$ is the magnetic vector potential, independently reveal the existence of two highly-anisotropic components of turbulence (Matthaeus et al. 1990; Tu and Marsch 1993; Bieber et al. 1996; Podesta and Gary 2011b; He et al. 2012b). The first component consists of highly-oblique fluctuations with $k_{\perp }\gg k_{\parallel }$. The second component consists of fluctuations that are more field-aligned ($k_{\perp }\ll k_{\parallel }$) and have lower amplitudes. This discovery led to the notion of the simultaneous existence of two-dimensional ($k_{\parallel }\simeq 0$) turbulent fluctuations and slab ($k_{\perp }\simeq 0$) wave-like fluctuations. Although this *slab+2D model* successfully reproduces the bimodal nature of the fluctuations in the solar wind, it does not account for a broader distribution of power in three-dimensional wavevector space.

Since waves and turbulence are interlinked through the concept of wave turbulence, a good understanding of the linear properties of plasma waves (Sect. 4.3) is important to understand the nature of the fluctuations and their dissipation mechanisms. By combining these concepts, we achieve a deeper insight into the dissipation mechanisms of turbulence. Working in the framework of wave turbulence, however, we emphasize again that we refer to waves as both the classical linear wave modes and the carriers of the turbulent fluctuations in wave turbulence.

### The concept of critical balance

*Critical balance* describes the state of strong wave turbulence in which the linear and the nonlinear timescales from Eqs. (184) and (185) are of the same order (Sridhar and Goldreich 1994; Goldreich and Sridhar 1995; Lithwick et al. 2007):

190 $$\begin{aligned} \omega _{{\mathrm {r}}}(k_{\parallel },k_{\perp })\sim k_{\perp }\,\delta U_{\perp }. \end{aligned}$$

The physics justification for critical balance is based on a causality argument (Howes 2015). Initially, a weak-turbulence interaction of two counter-propagating plasma waves as quantified in Eqs. (186) and (187) generates a pseudo-wave packet with $k_{\parallel }\simeq 0$ and with $k_{\perp }$ greater than that of either of the first two waves. However, causality forbids the final state of the turbulence from being completely two-dimensional. If it were, two planes at different locations along the background magnetic field would have to be identical if truly $k_{\parallel }=0$, which precludes any structure along $\mathbf{B}_0$ (Montgomery and Turner 1982). These two arbitrary planes, though, can only be identical if they are able to causally communicate with each other, which occurs via the exchange of Alfvén waves between them. This interplay between the generation of smaller $k_{\parallel }$ through weak-turbulence interactions and the requirement of causal connection along $\mathbf{B}_0$ creates a situation in which the timescale of the nonlinear interactions in one plane (i.e., $\tau _{\mathrm {nl}}$) is of order the timescale of the communication between the two planes (i.e., $\tau _{\mathrm {lin}}$). This describes the critical-balance condition in Eq. (190). In this model, the wave collision creates a pseudo-wave packet with $k_{\parallel }\simeq 0$, which then interacts with another propagating wave from the pool of fluctuations. This results in a new propagating wave with an even higher $k_{\perp }$. This multi-wave process, mediated by pseudo-wave packets and propagating wave packets, generates anisotropy while still satisfying causality through the field-parallel propagating waves. This process fills the *critical-balance cone*, which is the wavevector space satisfying Eq. (190), as it distributes power in three-dimensional wavevector space at increasing wavenumbers. Turbulence in the critical-balance state is still strong turbulence (rather than weak), notwithstanding that it retains properties of the associated plasma normal modes according to the wave-turbulence paradigm.

Although the justification of critical balance is still under debate (Matthaeus et al. 2014; Zank et al. 2017), there is a growing body of evidence from spacecraft measurements for the existence of conditions consistent with critical balance and wave turbulence in the solar wind (for a summary, see Chen 2016). We note, however, that the fluctuations in the solar wind do not consist of only one prescribed type of fluctuations (quasi-parallel waves, non-propagating structures and vortices, critically balanced wave turbulence, etc.) but rather a combination of these.

The concept of critical balance can be further illustrated in the MHD approximation (see Sect. 1.4.2), which has a long and successful history in plasma-turbulence research. For *incompressible MHD turbulence* ($\nabla \cdot \mathbf{U}=0$) consisting of transverse ($\delta \mathbf{B}\perp \mathbf{B}_0$ and $\delta \mathbf{U}\perp \mathbf{B}_0$) fluctuations, the Elsasser (1950) formulation of the MHD equations is a useful parameterization, which has been applied successfully to solar-wind measurements (Grappin et al. 1990; Marsch and Tu 1990a). We define the *Elsasser variables*

191 $$\begin{aligned} \mathbf{z}^{\pm }\equiv \delta \mathbf{U}\mp \frac{\delta \mathbf{B}}{\sqrt{4\pi \rho }} \end{aligned}$$

for forward (upper sign) and backward (lower sign) propagating Alfvén waves with respect to the background field $\mathbf{B}_0$. Using these variables, we rewrite the MHD momentum equation (51) and Faraday’s law (52) as

192 $$\begin{aligned} \frac{\partial \mathbf{z}^{\pm }}{\partial t}\pm \left( \mathbf{v}_{\mathrm {A}}^{*}\cdot \nabla \right) \mathbf{z}^{\pm }=-\left( \mathbf{z}^{\mp }\cdot \nabla \right) \mathbf{z}^{\pm }-\frac{1}{\rho }\nabla P_{\mathrm {tot}}, \end{aligned}$$

where $\mathbf{v}_{\mathrm {A}}^{*}\equiv \mathbf{B}_0/\sqrt{4\pi \rho }$ is the MHD Alfvén speed and $P_{\mathrm {tot}}\equiv P+B^2/8\pi$. The terms on the left-hand side of Eq. (192) represent the linear behavior of $\mathbf{z}^{\pm }$, while the terms on the right-hand side represent their nonlinear behavior. The linear terms are responsible for propagation effects, while the nonlinear terms are responsible for the cross-scale interactions, which are the building blocks of Alfvén-wave turbulence. Using Eqs. (184) and (185), we estimate the frequencies associated with the linear timescale $\tau _{\mathrm {lin}}$ and the nonlinear timescale $\tau _{\mathrm {nl}}$ from the spatial operators on $\mathbf{z}^{\pm }$ in Eq. (192) as

193 $$\begin{aligned} \frac{1}{\tau _{\mathrm {lin}}} \sim \left( \mathbf{v}_{\mathrm {A}}^{*}\cdot \nabla \right) \sim \frac{v_{\mathrm {A}}^{*}}{\ell _{\parallel }} \end{aligned}$$

and

194 $$\begin{aligned} \frac{1}{\tau _{\mathrm {nl}}}\sim \left( \mathbf{z}^{\mp }\cdot \nabla \right) \sim \frac{\delta U}{\ell _{\perp }}, \end{aligned}$$

where we define the characteristic scales $\ell _{\parallel }$ and $\ell _{\perp }$ parallel and perpendicular with respect to $\mathbf{B}_0$. In critical balance, $\tau _{\mathrm {lin}}\sim \tau _{\mathrm {nl}}$ so that

195 $$\begin{aligned} \frac{\delta U}{\ell _{\perp }}\sim \frac{v_{\mathrm {A}}^{*}}{\ell _{\parallel }}, \end{aligned}$$

which corresponds to $k_{\perp }\delta U\sim k_{\parallel }v_{\mathrm {A}}^{*}$ as in Eq. (190). Critical balance predicts that the inertial-range power spectrum of magnetic-field fluctuations in the direction perpendicular to $\mathbf{B}_0$ follows the Kolmogorov slope given by Eq. (180), where *k* is replaced by $k_{\perp }$. The inertial-range power spectrum of magnetic fluctuations in the direction parallel to $\mathbf{B}_0$ then follows $E(k_{\parallel })\sim k_{\parallel }^{-2}$.

The phenomenological model of *dynamic alignment* describes an extension of critical balance (Boldyrev 2005, 2006; Mallet et al. 2015). In this model, the turbulent velocity fluctuations $\delta \mathbf{U}$ increasingly align their directions with the directions of the mangetic-field fluctuations $\delta \mathbf{B}$ as the energy cascades toward smaller scales. This framework predicts two limits depending on the strength of the background magnetic field. If the background field is strong, the turbulent spectrum follows the Iroshnikov–Kraichnan slope given by Eq. (183), where *k* is replaced by $k_{\perp }$, in the perpendicular direction. Conversely, if the background field is weak, the perpendicular spectrum follows the Kolmogorov slope given by Eq. (180), where *k* is replaced by $k_{\perp }$. This prediction is consistent with MHD simulations of driven turbulence (Müller et al. 2003). In the fully aligned state, either $\mathbf{z}^+$ or $\mathbf{z}^-$ is exactly zero, so nonlinear interactions cease.

### Advanced topics

We briefly address three topics of great importance for solar-wind turbulence research that go beyond the direct focus of our review on the multi-scale nature of the solar wind: intermittency, reconnection, and anti-phase-mixing.

#### Intermittency

The two-point speed increment is defined as $\delta u(r)\equiv \langle U(x+r)-U(x)\rangle$, where *x* is the distance along a straight path through a volume of plasma and $\langle \cdots \rangle$ is the average over many *x*. Though the probability distribution of $\delta u(r)$ in the solar wind has a Gaussian distribution at larger scales *r*, it exhibits non-Gaussian features at smaller *r* (Marsch and Tu 1994; Sorriso-Valvo et al. 1999, 2001; Osman et al. 2014a). Specifically, the distribution develops enhanced tails, which indicate that sharp changes in velocity occur more frequently than predicted by Gaussian statistics. The increments in the magnetic field also exhibit this statistical property. These findings suggest that the solar-wind turbulence is *intermittent* (i.e., exhibiting bursty patches of increased turbulence) and forms localized regions of enhanced fluctuations.

The diagnostic called *Partial Variance of Increments (PVI)* is defined as (Greco et al. 2008)

196 $$\begin{aligned} \mathrm {PVI}\equiv \frac{\left| \delta \mathbf{B}(t,\tau )\right| }{\sqrt{\left\langle \left| \delta \mathbf{B}(t,\tau )\right| ^2\right\rangle }}, \end{aligned}$$

where $\delta \mathbf{B}(t,\tau )\equiv \mathbf{B}(t+\tau )-\mathbf{B}(t)$ is the magnetic-field increment in a time-series measurement of $\mathbf{B}(t)$ (Greco et al. 2018). PVI enables the identification of intermittency and allows for the statistical comparison of intermittency in plasma simulations and solar-wind observations (Wang et al. 2013; Greco et al. 2016). Large PVI values indicate *coherent structures*, which are organized and persistent turbulent flow patterns and are believed to be the building blocks of intermittency. Because non-linearities are locally quenched inside these coherent structures, they survive longer than the surrounding turbulence. The slow solar wind exhibits greater enhancements in PVI values than the fast solar wind (Servidio et al. 2011; Greco et al. 2012), which demonstrates that the slow solar wind contains a greater density of coherent structures than the fast solar wind (see also Bruno et al. 2003). Regions of increased plasma heating and non-Maxwellian features in the particle distribution functions tend to occur in and around coherent structures (Osman et al. 2011; Wan et al. 2012; Karimabadi et al. 2013; Wu et al. 2013; Wan et al. 2015; Parashar and Matthaeus 2016; Yang et al. 2017b).

Intermittency is a general feature known from fluid turbulence (McComb 1990). However, it remains unclear how intermittency and wave turbulence interact in the solar wind and what role intermittency plays in the dissipation of turbulence (Wang et al. 2014; Wan et al. 2015, 2016; Zhdankin et al. 2016; Perrone et al. 2017; Howes et al. 2018; Mallet et al. 2019).

#### Magnetic reconnection

*Magnetic reconnection* refers to the rearrangement of the magnetic field in a highly-conducting fluid through resistive diffusion, which leads to a conversion of magnetic-field energy into particle energy. In regard to plasma turbulence, magnetic reconnection is a process that is closely related to intermittency. Intermittency is associated with localized large gradients in the magnetic field, which, according to Ampère’s law in Eq. (23), corresponds to *current sheets*: localized regions of enhanced current $\mathbf{j}$, which are a type of coherent structure as introduced in Sect. 5.4.1 (Karimabadi et al. 2013; TenBarge and Howes 2013; Howes 2016). Current sheets are candidate regions for magnetic reconnection, which demonstrates the direct link between turbulence and reconnection (Matthaeus et al. 1984; Servidio et al. 2009, 2010; Osman et al. 2014b), and reconnection acts as a dissipation channel for the turbulent fluctuations (Retinò et al. 2007; Sundkvist et al. 2007; Cerri and Califano 2017; Shay et al. 2018). On the other hand, reconnection sites are inherently unstable to the *tearing instability*, which progressively fragments them into smaller and smaller current sheets (Loureiro et al. 2007; Lapenta 2008; Loureiro and Uzdensky 2016; Tenerani et al. 2016). In this way, reconnection sites generate a cascade to smaller scales by themselves and thus drive turbulence. In these progressively fragmented current sheets, the reconnection time gradually becomes faster than any other timescale, including the nonlinear time (Pucci and Velli 2014). When this condition is established, reconnection is able to interrupt the cascade of Alfvén-wave turbulence (Boldyrev and Loureiro 2017; Loureiro and Boldyrev 2017; Mallet et al. 2017). Therefore, reconnection must be considered when studying turbulence dynamics at small scales.

For further information on the connection between turbulence, coherent structures, and reconnection, we recommend the review article by Matthaeus and Velli (2011) and the comprehensive textbook by Frisch (1995).

#### Anti-phase-mixing

In Sects. 4.2.1 and 4.2.2, we discuss the formation of smaller velocity-space structure in the particle distribution function through linear and nonlinear phase mixing. *Anti-phase-mixing*, which is a stochastic variant of the *plasma echo* effect (Gould et al. 1967), is a process by which small-scale structure is removed from the distribution function in a turbulent plasma. For electrostatic turbulence, Parker et al. (2016) and Schekochihin et al. (2016) describe phase mixing and anti-phase-mixing in terms of the flux of energy in Hermite space of the particle distribution function. Phase mixing creates a transfer of energy from small to large Hermite moments. In a turbulent plasma with a low collision rate, a stochastic plasma echo creates a transfer of energy from large to small Hermite moments: effectively from small-scale structure to large-scale structure in velocity space. It therefore suppresses small-scale structure in the distribution function and thus non-Maxwellian features that may have otherwise led to collisional damping after ongoing phase mixing as described in Sect. 4.2.2. Anti-phase-mixing not only counteracts collisionless damping mechanisms but also leads to a fluid-like behavior of fluctuations even at low collisionality because higher-order-moment closures become unnecessary (Meyrand et al. 2019). This process is potentially responsible for the observed fluid-like behavior of compressive and KAW-like fluctuations in space plasmas (Verscharen et al. 2017; Wu et al. 2019).

## Kinetic microinstabilities

*Instabilities* are mechanisms that transfer energy from free-energy sources, such as the non-equilibrium particle distributions described in Sects. 1.4.4 and 1.4.5 or large-amplitude waves, to plasma normal modes that initially have amplitudes at the thermal-noise level (Rosenbluth 1965). The amplitude of these normal modes then grows exponentially with time as shown in Eq. (138),

197 $$\begin{aligned} A(\mathbf{x},t)\propto e^{\gamma t}, \end{aligned}$$

where $\gamma >0$ is the growth rate of the instability, out of the thermal noise during the linear phase of the instability, while it extracts energy from its free-energy source. After the linear phase, the normal-mode amplitude reaches some saturation level, at which point nonlinear behavior occurs that limits the exponential growth of the instability.

In this section, we focus on small-scale instabilities that have characteristic wavelengths of order the particle kinetic scales $d_j$ and $\rho _j$ and that affect the large-scale dynamic evolution of the solar wind. We divide these instabilities into two categories. First, we discuss those associated with non-thermal structure in the particle velocity distributions, including temperature anisotropies and beams. These instabilities lead to *wave–particle interactions* that drive unstable growth. Second, we discuss those instabilities caused by large-amplitude fluctuations, producing *wave–wave interactions* that drive unstable growth. This taxonomy provides the organizational structure for this section.

Generically, both types of instabilities generate small-scale fluctuations in the electric and/or magnetic field. While the turbulent cascade is dominated by interactions that are local in wavevector space (see Sect. 5.1), instabilities directly inject energy into the fluctuation spectrum at small scales. The scattering of particles on these small-scale field structures acts as an effective viscosity for the large-scale plasma behavior and thereby influences the thermodynamic evolution of the solar wind (Kunz et al. 2011, 2014; Rincon et al. 2015; Riquelme et al. 2015, 2016, 2017, 2018). As we focus on the effects of small-scale structure on larger-scale behavior, we point the interested reader to the complementary review by Matteini et al. (2012) on the complementary effects of large-scale solar-wind behavior on kinetic-scale phenomena. In particular, the discussion of the effects of background inhomogeneities at larger scales are left for later editions of this review.

### Wave–particle instabilities

Wave–particle instabilities are driven by departures of velocity distribution functions from the Maxwellian equilibrium given in Eq. (59). Such departures are frequently observed in the solar wind (see Sect. 1.4.4, 1.4.5), but not all of the associated energy is available to drive the system unstable. For instance, unequal temperatures between different plasma species are not known by themselves to drive wave–particle instabilities, which has major implications for accretion-disk dynamics in astrophysics (Begelman and Chiueh 1988; Narayan and McClintock 2008; Sironi and Narayan 2015). A non-Maxwellian velocity-space structure must conform to specific conditions in order to drive an instability: i.e., to transfer energy from the particles to the electric and magnetic fields. This process simultaneously leads to an exponentially growing mode and drives the system closer to *local thermodynamic equilibrium*. Once the system no longer meets the conditions for instability, the march toward equilibrium halts, and the system lingers in a state of *marginal stability*; i.e., the conditions for which $\gamma =0$. This effect has been identified in numerical simulations (Matteini et al. 2006; Hellinger and Trávníček 2008), but recent work suggests that dynamic interactions between the ions and electrons may modify the stability threshold conditions (Yoon and Sarfraz 2017). Gary (1993) and Yoon (2017) offer more details into the theory of unstable wave–particle interactions in the solar wind.

A variety of different schemes are used to classify wave–particle instabilities (Krall and Trivelpiece 1973; Treumann and Baumjohann 1997; Schekochihin et al. 2010; Klein and Howes 2015). Most focus on the spatial scales at which unstable modes are driven: *macroinstabilities* and *microinstabilities* respectively drive unstable modes with wavelengths much greater than and comparable to kinetic scales. Other classifications focus on the mechanisms that drive the unstable modes: *configuration-space instabilities* are driven by the departure of macroscopic quantities from thermodynamic equilibrium and thus can be modeled by fluid equations, and *kinetic* or *velocity-space instabilities* are driven by resonant interactions with structures in the particle velocity distributions.

A prototypical macroscopic configuration-space instability is the Chew–Goldberger–Low (CGL) firehose instability (Chew et al. 1956), in which the pressure $p_{\perp }$ perpendicular to the magnetic field becomes insufficient to counteract the centrifugal force experienced by the particles along a bend in the magnetic field. Without a sufficiently robust restoring force, initial magnetic perturbations are not damped but in fact amplified, leading to the growth of a large-scale unstable Alfvén mode.^20^

**Fig. 20:**
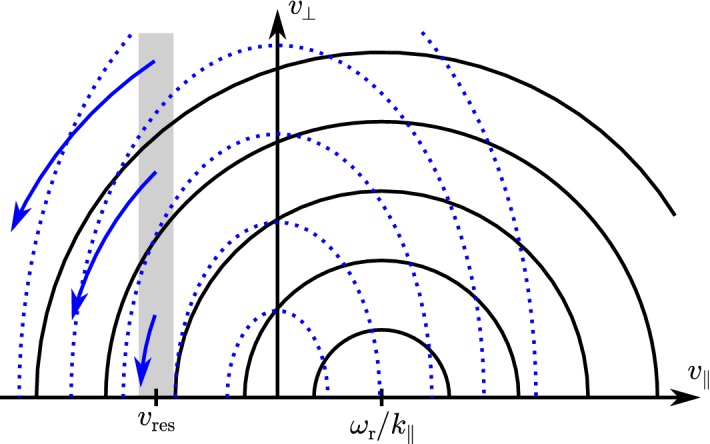
Quasilinear diffusion for an anisotropic particle distribution $f_{0j}$ (isocontours shown as blue dashed lines) unstable to left-hand circularly polarized ion-cyclotron waves with frequency $\omega _{\mathrm {r}}$ and parallel wavenumber $k_\parallel$. Unlike the cyclotron-resonant damping case (Fig. 15), the velocity-space diffusion along the pitch-angle gradients of $f_{0j}$ (black semi-circles) at $v_{\parallel }=v_{\text {res}}$ (gray shaded area) causes resonant particles to lose kinetic energy (i.e., to decrease in $v_{\perp }^2+v_{\parallel }^2$), which is transferred to the growing electromagnetic wave. This mechanism drives the kinetic ion-cyclotron instability

A typical microscopic kinetic instability is the *ion-cyclotron instability*, which is physically very similar to the cyclotron-resonant damping of A/IC waves discussed in Sect. 4.2.1 but with $\gamma >0$. A left-hand circularly polarized wave with finite $k_{\parallel }$ may resonantly interact with particles from a narrow range of parallel velocities $\approx v_{\mathrm {res}}$ that satisfy the resonance condition in Eq. (157) for $n=+\,1$. These resonant particles diffuse according to the quasilinear diffusion relation in Eq. (154) along trajectories tangent to semi-circles defined by Eq. (158) around the point $(v_{\perp },v_{\parallel })=(0,\omega _{\mathrm {r}}/k_{\parallel })$ in velocity space. At the same time, quasilinear diffusion demands that the particles diffuse from higher $f_{0j}$ toward lower $f_{0j}$. We discuss the differences between the damped and the unstable cases with the help of Fig. 20, which shows the same situation as Fig. 15 but a different shape of $f_{0j}$ (blue dashed lines). This new shape of $f_{0j}$ now exhibits a temperature anisotropy with $T_{\perp \mathrm {p}}>T_{\parallel \mathrm {p}}$, which causes particles to diffuse toward smaller $v_{\perp }$ in Fig. 20 rather than toward larger $v_{\perp }$ as in Fig. 15. This change in behavior is a direct consequence of the altered alignment between the diffusion paths (black semi-circles) and the contours of $f_{0j}$ (blue dashed lines). The diffusive particle motion now causes the resonant particles to lose kinetic energy (i.e., a decrease in $v_{\perp }^2+v_{\parallel }^2$), which is transferred to growing field fluctuations. Importantly, the direction of the energy flow between the fields and the particle distribution depends on the local sign of the *pitch-angle gradient* of $f_{0j}$ at the resonance speed according to Eq. (155). In addition to temperature anisotropies, drifting populations and other non-Maxwellian features can lead to pitch-angle gradients that drive resonant instabilities.

Despite their apparent similarity, the macro/micro and configuration/kinetic schemes are not synonymous. Some instabilities occur at large spatial scales but are driven by velocity-space effects. For example, the mirror-mode instability (Southwood and Kivelson 1993) is driven by the interaction between the slow-mode-like anti-phase response of bulk thermal and magnetic fluctuations, $\delta p$ and $\delta |\mathbf{B}|$, and the in-phase response felt by particles with $v_\parallel \sim 0$. This latter population is approximately stationary along the background magnetic field and gains or loses energy with changes in the magnetic-field strength. On the other hand, the bulk population, which does move parallel to the magnetic field in a slow-mode-like polarized wave (see Sect. 4.3.4), is able to effectively conserve energy via transfer between parallel and perpendicular degrees of freedom.

The numerical evaluation of linear instabilities in kinetic theory follows the same procedure as the numerical evaluation of wave dispersion relations described in Sect. 4.1: the linearized Vlasov equation is used to calculate the dielectric tensor $\varvec{\epsilon }$. Solutions to the dispersion relation in Eq. (152) with $\gamma >0$ for a particular wavevector $\mathbf{k}$ represent linear kinetic instabilities, which grow with time according to Eq. (197). Following from the linear set of Vlasov–Maxwell equations, these solutions are independent of the fluctuation amplitude. In contrast, the wave–wave instabilities discussed in Sect. 6.2 depend on fluctuation amplitude.

The behavior of instabilities in the inhomogeneous and turbulent solar wind as well as the nonlinear evolution of plasma instabilities are important matters of ongoing research. Most numerical evaluations of linear instabilities assume homogeneous plasma conditions, which are not fulfilled in the solar wind in general. For instance, the expansion of the plasma, the interaction of different plasma streams, and the ubiquitous turbulence create inhomogeneities and temporal variability that call into question the assumption of homogeneity. Nevertheless, the solar wind’s parameter space is often observed to be restricted by the linear-instability thresholds, which suggests that linear theory bears some applicability to the solar wind.

We define the *marginal stability threshold* as a contour of constant maximum growth rate $\gamma _{\mathrm {m}}$ at any $\mathbf{k}$ through parameter space for a given instability. The choice of the relevant $\gamma _{\mathrm {m}}$ is somewhat arbitrary. Assuming that only a couple of parameters (e.g., $\beta _{\parallel j}$ and $T_{\perp j}/T_{\parallel j}$) have a significant impact on the growth rate of a specific instability, it is possible to construct a parametric model for the instability threshold. The inverse relation between a species’ temperature anisotropy and $\beta _{\parallel j}$ serves as the prototypical example of such a threshold model, given for instance by Gary et al. (1994a, b), Gary and Lee (1994), and Hellinger et al. (2006):

198 $$\begin{aligned} \frac{T_{\perp j}}{T_{\parallel j}} = 1 + \frac{a}{\left( \beta _{\parallel j}-c\right) ^b}, \end{aligned}$$

where *a*, *b*, and *c* are constant parameters calculated from fits to solutions of the hot-plasma dispersion relation. This form for the inverse relation is introduced by Hellinger et al. (2006) for a bi-Maxwellian proton background distribution function according to Eq. (61) and an isotropic Maxwellian electron distribution. The values of *a*, *b*, and *c* are different for the four unstable modes that can be driven by proton temperature anisotropies (i.e., the ion-cyclotron, parallel firehose, mirror-mode, or oblique firehose instability), as well as the desired maximum growth rates. Verscharen et al. (2016) compare the parameters *a*, *b*, and *c* for thresholds depending on maximum growth rates. Table 3 lists best-fit values for these parameters for three different $\gamma _{\mathrm {m}}/\varOmega _{\mathrm {p}}$-values for each of the four instabilities driven by proton temperature anisotropy. The growth rates have been calculated for a quasi-neutral plasma consisting of bi-Maxwellian protons and Maxwellian electrons with $T_{\mathrm {e}}=T_{\parallel \mathrm {p}}$ and $v_{\mathrm {Ap}}/c=10^{-4}$. The values of *a*, *b*, and *c* change in the presence of other plasma components, including beams and minor ion components, which may act as additional sources of free energy or may stabilize unstable growth (Price et al. 1986; Podesta and Gary 2011a; Maruca et al. 2012; Matteini et al. 2015a). If the underlying distribution has a shape other than bi-Maxwellian—e.g., if the particles have a $\kappa$-distribution according to Eq. (62) or a bi-$\kappa$-distribution according to Eq. (63)—these threshold curves can be significantly different (Summers and Thorne 1991; Xue et al. 1993; Summers et al. 1994; Xue et al. 1996; Astfalk et al. 2015; Astfalk and Jenko 2016). The exploration of more general phase-space densities requires direct numerical integration of the dispersion relation (Dum et al. 1980; Matsuda and Smith 1992; Astfalk and Jenko 2017; Horaites et al. 2018a; Verscharen et al. 2018). Such general distributions produce instabilities that are either enhanced or suppressed relative to those associated with bi-Maxwellian particle distributions.

**Table 3:** Fit parameters for isocontours of constant maximum growth rate $\gamma _{\mathrm {m}}=10^{-2}\varOmega _{\mathrm {p}}$, $\gamma _{\mathrm {m}}=10^{-3}\varOmega _{\mathrm {p}}$, and $\gamma _{\mathrm {m}}=10^{-4}\varOmega _{\mathrm {p}}$ in the $\beta _{\parallel \mathrm {p}}{-}T_{\perp \mathrm {p}}/T_{\parallel \mathrm {p}}$ plane for use in Eq. (198)

Instability	*a*	*b*	*c*
$$\gamma _{\mathrm {m}}=10^{-2}\varOmega _{\mathrm {p}}$$			
Ion-cyclotron	0.649	0.400	0.000
Mirror-mode	1.040	0.633	$-$ 0.012
Parallel firehose	$-$ 0.647	0.583	0.713
Oblique firehose	$-$ 1.447	1.000	$-$ 0.148
$$\gamma _{\mathrm {m}}=10^{-3}\varOmega _{\mathrm {p}}$$			
Ion-cyclotron	0.437	0.428	$-$ 0.003
Mirror-mode	0.801	0.763	$-$ 0.063
Parallel firehose	$-$ 0.497	0.566	0.543
Oblique firehose	$-$ 1.390	1.005	$-$ 0.111
$$\gamma _{\mathrm {m}}=10^{-4}\varOmega _{\mathrm {p}}$$			
Ion-cyclotron	0.367	0.364	0.011
Mirror-mode	0.702	0.674	$-$ 0.009
Parallel firehose	$-$ 0.408	0.529	0.410
Oblique firehose	$-$ 1.454	1.023	$-$ 0.178

Calculated with the NHDS code (Verscharen and Chandran 2018) and adapted from Verscharen et al. (2016)

**Table 4:** Wave–particle instabilities relevant to the solar wind organized by free-energy source

Instability	Classification	Unstable normal mode	References
$T_{\perp i}/T_{\parallel i}>1$ ${}^{\mathrm{a}}$			
Ion-cyclotron	Micro/resonant	Parallel A/IC	Kennel and Petschek (1966) and Davidson and Ogden (1975)
Mirror-mode	Macro/resonant	Non-propagating oblique kinetic slow mode	Tajiri (1967), Southwood and Kivelson (1993) and Kivelson and Southwood (1996)
$$T_{\perp i}/T_{\parallel i}<1$$			
Parallel firehose	Micro/resonant	Parallel FM/W	Quest and Shapiro (1996) and Gary et al. (1998)
Oblique firehose	Micro/resonant	Non-propagating oblique Alfvén	Hellinger and Matsumoto (2000)
$$T_{\perp \mathrm {e}}/T_{\parallel \mathrm {e}}<1$$			
Parallel electron firehose	Micro/resonant	Parallel FM/W	Hollweg and Völk (1970) and Gary and Madland (1985)
Oblique electron firehose	Micro/configuration	Oblique non-propagating Alfvén	Li and Habbal (2000) and Kunz et al. (2018)
$$T_{\perp \mathrm {e}}/T_{\parallel \mathrm {e}}>1$$			
Whistler anisotropy	Micro/resonant	Parallel FM/W	Kennel and Petschek (1966) and Scharer and Trivelpiece (1967)
$P_{\perp }/P_{\parallel }<1$ ${}^{\mathrm{b}}$			
CGL firehose	Macro/configuration	Non-propagating oblique Alfvén	Chew et al. (1956)
Electromagnetic beam			
Ion/ion RH resonant	Micro/resonant	Parallel FM/W	Barnes (1970)
Ion/ion nonresonant	Macro/configuration	Backward propagating firehose-like	Sentman et al. (1981) and Winske and Gary (1986)
Ion/ion LH resonant	Micro/resonant	Parallel A/IC	Sentman et al. (1981)
Electron/ion	Micro/resonant	FM/W and A/IC modes	Akimoto et al. (1987)
Electron heat flux	Micro/resonant	Parallel FM/W	Gary et al. (1975, 1994c, 1999), Gary and Li (2000), Horaites et al. (2018a) and Tong et al. (2018)
Ion drift	Micro/resonant	Parallel and oblique FM/W	Verscharen and Chandran (2013)
Ion drift	Micro/resonant	Parallel and oblique A/IC	Verscharen and Chandran (2013)
Ion drift and anisotropy	Micro/resonant	Parallel FM/W and A/IC	Verscharen et al. (2013a) and Bourouaine et al. (2013)

For each instability, we list its name, classification, name of the unstable normal mode, and further references

${}^{\mathrm{a}}$Resonant instabilities due to temperature anisotropies can arise for each ion species (index *i*; see Maruca et al. 2012)

${}^{\mathrm{b}}$ Configuration-space instabilities are triggered by contributions to the total excess pressure from each plasma species (Kunz et al. 2015; Chen et al. 2016)

Table 4 lists the wave–particle instabilities that are most important in regulating the large-scale dynamics of the solar wind. Many foundational publications (e.g., Hollweg 1975; Schwartz and Roxburgh 1980; Gary 1993) provide more complete catalogues.

Two of the most common free-energy sources are distinct temperatures or pressures perpendicular and parallel to the background magnetic field and the presence of faster populations that form a shoulder on or a beam distinct from the core population (Fig. 4). These two specific cases are considered in Sects. 6.1.1 and 6.1.2, with particular emphasis on their impact on the macroscale behavior of the solar wind. Significant work has been done on the effects of instabilities in other space environments such as the magnetosphere and magnetosheath (Maruca et al. 2018, and references therein), but these results lie beyond the scope of this work.

#### Temperature anisotropy

**Fig. 21:**
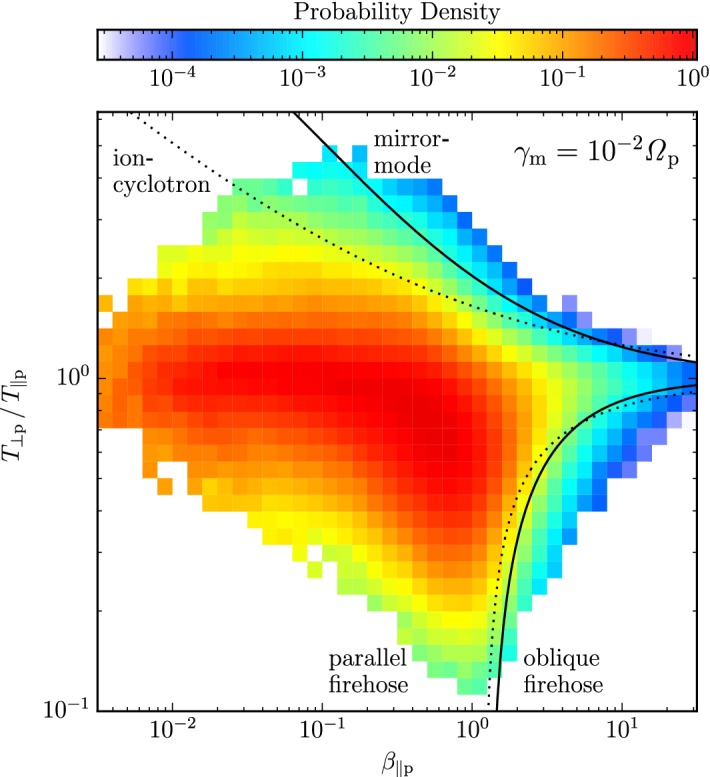
Probability distribution of the pristine solar wind in the $\beta _{\parallel \mathrm {p}}$–$T_{\perp \mathrm {p}}/T_{\parallel \mathrm {p}}$ plane. The instability thresholds for the four instabilities associated with proton temperature anisotropy according to Eq. (198) and Table 3 with $\gamma _{\mathrm {m}}=10^{-2}\varOmega _{\mathrm {p}}$ are plotted for comparison. We only plot bins containing at least 25 counts. A significant fraction of the distribution exceeds the two resonant thresholds (ion-cyclotron and parallel firehose), while the non-resonant mirror-mode and oblique-firehose thresholds set more precise boundaries to the data distribution

Wave–particle instabilities associated with *temperature anisotropies* serve as a canonical example for the effects of wave–particle instabilities on the solar wind’s large-scale evolution. Initial investigations of instability limits on solar-wind proton temperature anisotropy address either the $T_{\perp \mathrm {p}}>T_{\parallel \mathrm {p}}$ limit or the $T_{\perp \mathrm {p}}<T_{\parallel \mathrm {p}}$ limit separately. For the former, Gary et al. (2001) find that the ion-cyclotron stability threshold limits the maximum anisotropy of observations from the ACE spacecraft. For the latter limit, Kasper et al. (2002) find that the Wind spacecraft’s temperature-anisotropy values are mostly bounded by the parallel firehose instability threshold. Subsequent work (Hellinger et al. 2006) shows that, for the slow solar wind, the distribution of temperature anisotropies is well constrained for $T_{\perp \mathrm {p}}/T_{\parallel \mathrm {p}}>1$ and $T_{\perp \mathrm {p}}/T_{\parallel \mathrm {p}}<1$ by the threshold of each of the configuration-space instabilities: i.e., the mirror-mode and oblique firehose instabilities. The probability distribution of data in the $\beta _{\parallel \mathrm {p}}{-}T_{\perp \mathrm {p}}/T_{\parallel \mathrm {p}}$ plane using measurements from the Wind spacecraft is illustrated in Fig. 21.^21^ We use the same dataset as described by Maruca and Kasper (2013).

Interestingly, as seen in Fig. 21, the solar wind is not constrained by all possible temperature-anisotropy thresholds: a significant portion of the $\beta _{\parallel \mathrm {p}}{-}T_{\perp \mathrm {p}}/T_{\parallel \mathrm {p}}$ distribution extends beyond the ion-cyclotron threshold, which, for $\beta _{\parallel \mathrm {p}}\lesssim 1$, sets a stricter limit on the departure from isotropy than the mirror-mode instability threshold, as is pointed out by Hellinger et al. (2006). Several justifications for this apparent inactivity of the ion-cyclotron instability have been proposed: low efficiency of energy extraction (Shoji et al. 2009), stabilizing effects of minor ions and/or drifts (Maruca 2012; Maruca et al. 2012), or quasilinear flattening of the resonant region (Isenberg et al. 2013).

A naïve model for the expanding solar wind would have $T_{\perp j}$ and $T_{\parallel j}$ follow the double-adiabatic prediction [see Eqs. (44) and (45) in Sect. 1.4.1]. Using data from Helios and Ulysses at different heliocentric distances, Matteini et al. (2007) show that the distribution in $\beta _{\parallel \mathrm {p}}{-}T_{\perp \mathrm {p}}/T_{\parallel \mathrm {p}}$ space follows a radial trend, albeit one with a smaller radial gradient than that predicted by double-adiabatic expansion, until the system encounters the instability thresholds. Then, the distribution’s anisotropy is constrained by the parametric thresholds to the stable parameter space.

Identifying polarization and other linear quantities associated with the predicted instabilities allows us to infer the presence of modes driven by temperature-anisotropy instabilities. For instance, the signal of strongly peaked magnetic helicity near parallel ion-kinetic scales (He et al. 2011; Podesta and Gary 2011b; Klein et al. 2014b) indicates the presence of parallel-propagating FM/W or A/IC waves associated with proton temperature-anisotropy instabilities. Wind observations provide evidence for enhanced magnetic fluctuations near threshold boundaries (Bale et al. 2009), suggesting that instabilities are active near these thresholds in generating unstable modes which are associated with such fluctuations. Ion temperature (Maruca et al. 2011; Bourouaine et al. 2013) and intermittency (Osman et al. 2012; Servidio et al. 2014) are also found to be enhanced in marginally unstable parameter regions. Calculating polarization as a function of $T_{\perp \mathrm {p}}/T_{\parallel \mathrm {p}}$ and $\beta _{\parallel \mathrm {p}}$ reveals the presence of a population of A/IC waves in the region in which they are expected to become unstable (Telloni and Bruno 2016). The identification of parallel-propagating A/IC waves (e.g., Jian et al. 2009, 2010, 2014; Gary et al. 2016b) that do not naturally arise from critically balanced turbulence (see Sect. 5.3) serves as further, indirect evidence for the action of these instabilities.

We emphasize that caution must be exercised in the analysis of $\beta _{\parallel j}{-}T_{\perp j}/T_{\parallel j}$ plots. Hellinger and Trávníček (2014) raise concerns about the effects of projecting the distribution of quantities onto any reduced parameter space. By partitioning the data into different temperature quartiles and studying the temperature-anisotropy distribution of each, they find that enhanced quantities near the instability thresholds may primarily result from underlying correlations between solar-wind temperatures and speeds. Moreover, it is important to carefully account for the blurring of temperature-anisotropy observations due to the finite time required to construct a velocity distribution measurement (Verscharen and Marsch 2011; Maruca and Kasper 2013).

In addition to instabilities triggered by the temperature anisotropy of the core proton velocity distribution, anisotropic distributions of the other plasma components, including the electrons (Hollweg and Völk 1970; Gary and Madland 1985; Li and Habbal 2000; Kunz et al. 2018) and heavy ions (Ofman et al. 2001; Maruca et al. 2012; Bourouaine et al. 2013) can lead to resonant instabilities. We discuss the combined effect of these sources of free energy in Sect. 6.1.3.

#### Beams and heat flux

The *relative drift* between plasma components is another common source of free energy that can drive wave–particle instabilities. The velocity difference between the two components (of the same or different species) can contribute to excess parallel pressure or induce non-zero currents, and the drifting distributions themselves may resonate with unstable waves (e.g., the parallel propagating beam instability described by Verscharen et al. 2013b). As with temperature anisotropies, some thresholds associated with drifts and beams constrain the observed data distributions in parameter space.

Beam and heat-flux instabilities regulate non-thermal features in the electron distribution function. For instance, Tong et al. (2018) find compelling evidence that the heat-flux-driven Alfvén-wave instability limits the electron core drift with respect to the halo and the protons. To some degree, this result contradicts the earlier work of Bale et al. (2013), who find that the collisional transport rather than a heat-flux instability is more active in limiting the electron-core drift (see also Sect. 3.3.2). However, collisions and kinetic instabilities can co-exist in the solar wind and simultaneously regulate the heat flux. The electron-strahl heat flux can drive oblique instabilities of the lower-hybrid and the oblique FM/W wave (Omelchenko et al. 1994; Shevchenko and Galinsky 2010; Vasko et al. 2019; Verscharen et al. 2019a).

**Fig. 22:**
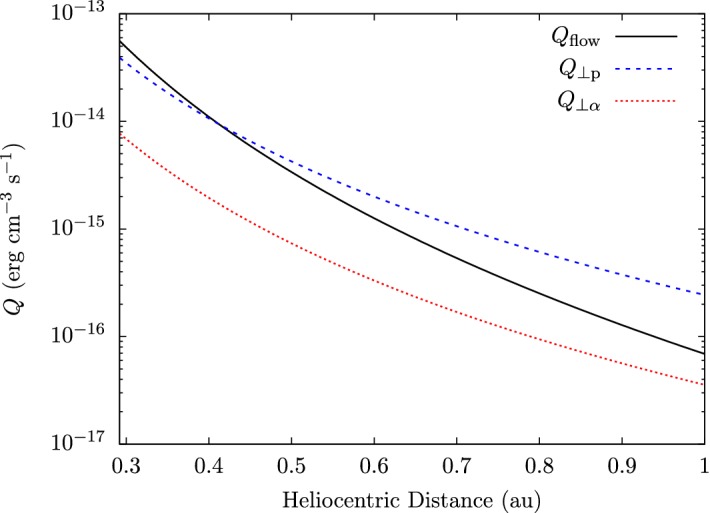
Rate of energy release $Q_{\mathrm {flow}}$ from the deceleration of $\alpha$-particles through kinetic microinstabilities as a function of distance in the inner heliosphere. We assume that the $\alpha$-particle drift speed is always fixed to the local threshold for the FM/W instability based on average fast-solar-wind measurements from Helios. $Q_{\mathrm {flow}}$ then follows from energy conservation. $Q_{\perp \mathrm {p}}$ and $Q_{\perp \alpha }$ are calculated based on Eq. (44), setting $\mathbf{q}_{\perp j}=0$ and the right-hand side to $Q_{\perp j}$. Using empirical profiles for *B*, $p_{\perp j}$, $n_j$, and $\mathbf{U}_j$ for $j=\mathrm {p}$ and $j=\alpha$ then gives the empirical heating rates $Q_{\perp \mathrm {p}}$ and $Q_{\perp \alpha }$. Adapted from Verscharen et al. (2015)

Likewise, ion beams can drive plasma instabilities. Bourouaine et al. (2013) report constraints on the drift of $\alpha$-particles relative to protons through parallel-propagating A/IC and FM/W instabilities. These ion-beam instabilities result in a quasi-continuous deceleration of the $\alpha$-particles, which leads to a quasi-continuous release of energy from the $\alpha$-particle kinetic energy into field fluctuations (Verscharen et al. 2015). Figure 22 shows, as functions of distance from the Sun, the rate of energy-density release $Q_{\mathrm {flow}}$ derived from energy conservation as well as the empirical perpendicular heating rates $Q_{\perp \mathrm {p}}$ for protons and $Q_{\perp \alpha }$ for $\alpha$-particles. $Q_{\mathrm {flow}}>Q_{\perp \alpha }$ at distances between 0.3 and 1 au, and $Q_{\mathrm {flow}}>Q_{\perp \mathrm {p}}$ at distances between 0.3 and 0.4 au. This finding suggests that the energy release through $\alpha$-particle instabilities comprises a significant fraction of the solar wind’s overall energy, and that large-scale solar-wind models must account for $\alpha$-particle thermodynamics. Due to the lack of in-situ measurements at smaller heliocentric distances, we are unable to compare $Q_{\mathrm {flow}}$ with $Q_{\perp \mathrm {p}}$ or $Q_{\perp \alpha }$ closer to the Sun yet; however, we expect this trend to continue toward the acceleration region of the solar wind.

#### Multiple sources of free energy

Under typical solar-wind conditions, *multiple sources of free energy* are simultaneously available to drive distinct unstable modes. For example, beams, temperature anisotropies, and anisothermal temperatures between species are all frequently and simultaneously present in solar-wind plasma (Kasper et al. 2008, 2017). The introduction of an additional source of free energy can act either to enhance an instability’s growth rate or act to stabilize the system.

The thresholds of configuration-space instabilities (i.e., the mirror-mode and the oblique firehose instabilities) depend on the total free energy in the system (Chen et al. 2016). The threshold of the oblique firehose instability limits the observed plasma to the stable parameter space, when the combined effects of ion and electron anisotropies as well as relative drifts between the plasma species are considered. Less than $1\%$ of the observations exceed this threshold, and, for these intervals, the proton, electron, and $\alpha$-particle components all significantly contribute to the system’s unstable growth.

According to an analytical model of the coupling between the effects of temperature anisotropy and drifts (Ibscher and Schlickeiser 2014), the combined effects of these free-energy sources yield a threshold in the region of parameter space with $\beta _{\parallel \mathrm {p}}<1$ and $T_{\perp \mathrm {p}}<T_{\parallel \mathrm {p}}$. This is consistent with the lack of solar-wind observations in this region of parameter space (see Fig. 21). However, Bale et al. (2009) do not find enhanced fluctuations or other indications of unstable-mode generation in this region, and Vafin et al. (2019) explain the lack of data in this region through collisional effects. The coupling of temperature anisotropy and beams has been incorporated into an improved threshold model for limiting proton-temperature-anisotropy observations (Vafin et al. 2018), which may be tested in future in-situ observations of low-$\beta _{\parallel \mathrm {p}}$ systems such as the near-Sun solar wind. Verscharen et al. (2013a) provide testable limits on temperature anisotropy and $\alpha$-particle drifts, which Bourouaine et al. (2013) find to largely agree with solar-wind observations. Numerical simulations (e.g., by Maneva and Poedts 2018) are also used to study the simultaneous impact of drifts and temperature anisotropies. The coupling between electrons and ions modifies the solar-wind expansion, preventing a uniform progression of the bulk thermodynamic properties toward the firehose threshold (Yoon and Sarfraz 2017). This effect occurs in addition to the effects of collisions on drawing the solar wind toward isotropy (see Sect. 3.3), which is found to be important but insufficient for a complete description of the solar wind’s observed state (Yoon 2016).

**Fig. 23:**
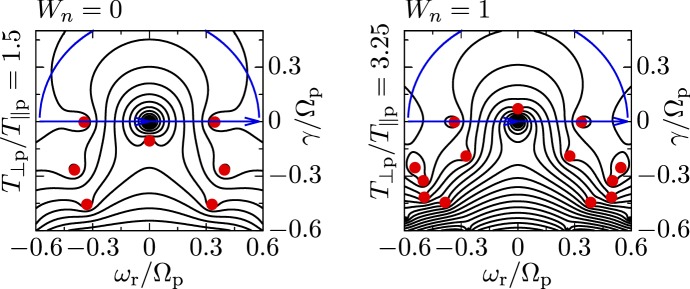
Illustration of the *Nyquist instability criterion*. Black lines indicate isocontours of $\mathrm {det}\,\left[ {{\mathcal {{\varvec{D}}}}}(\mathbf{k},\omega _{\text {r}}+i\gamma )\right]$ for a stable (left) and unstable (right) system, with the normal-mode solutions indicated with red dots. The contour integral is performed over the entire upper half plane, symbolized by the blue curve (which would formally extend out to $\omega _{\mathrm r}\rightarrow \pm \infty$). Applying the residue theorem yields a non-negative integer $W_n$ equal to the number of unstable modes supported by the system

Instead of relying solely on analytical threshold models, which are formally valid for low-dimensional sub-spaces (e.g., $\beta _{\parallel \mathrm {p}}$ and $T_{\perp \mathrm {p}}/T_{\parallel \mathrm {p}}$ only) of the full parameter space that characterizes the solar wind, the *Nyquist instability criterion* accounts for the simultaneous effects of all wave–particle free-energy sources (Nyquist 1932). This method determines whether a system supports any growing modes at a particular given wavevector $\mathbf{k}$ by performing a complex contour integration, which is illustrated in Fig. 23. The normal modes of a system are the solutions to $\mathrm {det}\,\left[ \mathbf{{\mathcal {D}}}(\mathbf{k},\omega )\right] =0$ according to Eq. (152), where $\mathbf{{\mathcal {D}}}$ is the system’s dispersion tensor. As described in Sect. 4.1, the form of $\mathbf{{\mathcal {D}}}$ depends on the set of system parameters such as temperature, density, and drift of each plasma component. The number of modes satisfying $\gamma >0$ can be ascertained by applying the residue theorem to the integral

199 $$\begin{aligned} W_n = \frac{1}{2 \pi i} \oint \frac{\mathrm {d} \omega }{\mathrm {det}\,\left[ \mathbf{{\mathcal {D}}}(\mathbf{k},\omega )\right] }, \end{aligned}$$

where the contour is taken over the upper half plane of complex frequency space $\omega =\omega _{\mathrm {r}}+i\gamma$. The integration in Eq. (199) is much easier to compute than the determination of the dispersion relation for all individual potentially unstable modes. This method has more than half a century of productive use in the study of plasma stability (Jackson 1958; Buneman 1959; Penrose 1960; Gardner 1963).

Klein et al. (2017) present a modern automatic implementation of the Nyquist instability criterion for the case of an arbitrary number of drifting bi-Maxwellian components. The application of this criterion to a statistically random set of solar-wind observations modeled as a collection of proton core, proton beam, and $\alpha$-particle components (each with distinct anisotropies, densities, and drifts) finds that a majority of intervals are unstable (Klein et al. 2018). Most of the unstable modes are resonant instabilities at ion-kinetic scales and with growth rates less than the instrument integration time and convected kinetic scales. About $10\%$ of the intervals have instabilities with growth rates of order the nonlinear turbulent cascade rate $1/\tau _{\mathrm {nl}}$ at proton-kinetic scales, which indicates that they may grow quickly enough to compete with the background turbulence.

### Wave–wave instabilities

Wave–wave instabilities, in contrast to wave–particle instabilities, depend sensitively on the amplitudes of the plasma fluctuations. The finite amplitudes of fluctuating waves lead to violations of the linearization used to derive the wave–particle instabilities discussed in Sect. 6.1. Instead, nonlinear effects allow for wave–wave coupling to lead to unstable wave growth, which places limits on the amplitudes of magnetic and velocity fluctuations.

#### Parametric-decay instability

The *parametric-decay instability* (PDI) is a classic wave–wave instability first described by Galeev and Oraevskii (1963) and Sagdeev and Galeev (1969) for a three-wave interaction. It belongs to a broader class of parametric instabilities that also includes beat and modulational instabilities (Hollweg 1994). In the low-$\beta _{\mathrm {p}}$ limit, the PDI causes a finite-amplitude forward-propagating Alfvén wave, known as the *pump mode*, to decay into a backward-propagating Alfvén wave and a forward-propagating acoustic wave. Goldstein (1978) provides a generalization of this instability for circularly-polarized Alfvén waves in finite-$\beta _{\mathrm {p}}$ plasmas. The dynamics of such instabilities are important for the evolution of the solar wind. As described in Sect. 4.3.4, the compressive acoustic mode can efficiently dissipate and thus heat the plasma (Barnes 1966). Furthermore, the generation of counter-propagating Alfvén waves is essential for driving the turbulent cascade (see Sect. 5.2). Malara and Velli (1996) show that, even in the large-amplitude limit and when the pump mode is non-monochromatic, the PDI continues to operate without a significant reduction in its growth rate. Theoretical work suggests that the PDI may develop an inverse cascade near the Sun and, therefore, be essential in driving solar-wind turbulence (Chandran 2018).

A number of numerical simulations investigate the presence and effects of decay instabilities under conditions approximating the solar wind (Matteini et al. 2010; Verscharen et al. 2012b; Tenerani and Velli 2013, 2017; Shoda and Yokoyama 2018; Shoda et al. 2018). A recent analysis of solar-wind observations at 1 au (Bowen et al. 2018) indicates a strong correlation between observed compressive fluctuations and higher estimated PDI growth rates, which is consistent with the parametric decay of Alfvén modes. Parametric instabilities are also observed in laboratory plasma experiments (Dorfman and Carter 2016).

#### Limits on large-amplitude magnetic fluctuations

In addition to decay instabilities, finite-amplitude waves are capable of *self-destabilization*. Linearly polarized, large-amplitude Alfvén waves drive compressions in the plasma, which reduce the amplitude of the Alfvénic fluctuations if $\delta |\mathbf{B}|\ne 0$ (see also Sect. 4.3.1 of this review; Hollweg 1971). This effect may lead to the observed preference for Alfvénic fluctuations with $B=\mathrm {constant}$. A related example of such behavior occurs if the amplitude $\delta B_\perp /B_0$ of the perpendicular magnetic fluctuations exceeds the threshold $\sim \beta _{\mathrm {p}}^{-1/2}$ (Squire et al. 2016). Beyond this limit, the pressure anisotropy associated with the wave fluctuations exceeds the parallel-firehose limit and destroys the restoring force associated with the magnetic tension, which destabilizes the wave. Numerical simulations confirm signatures of this instability, which are currently also being sought in solar-wind observations under high-$\beta _{\mathrm {p}}$ conditions (Squire et al. 2017a, b; Tenerani and Velli 2018).

### The fluctuating-anisotropy effect

Large-scale compressive fluctuations with finite amplitudes and $\omega _{\mathrm {r}}\ll \varOmega _{\mathrm {p}}$ modify the plasma moments, including $\beta _j$ and $T_{\perp j}/T_{\parallel j}$ according to Eqs. (44) and (45). These and potentially other plasma moments (like the relative drifts between species) fluctuate with the large-scale compressive fluctuations (Squire et al. 2017a, b; Tenerani and Velli 2018). If the amplitude of these fluctuations is sufficiently large, these modifications can move the system from a stable to an unstable configuration with respect to anisotropy-driven kinetic microinstabilities (Verscharen et al. 2016). The instability then acts to modify the velocity distribution, e.g., by pitch-angle scattering particles. It suppresses further growth of the anisotropy, which leads to a reduction in the amplitude of the large-scale compressive fluctuations and an isotropization of the particles. Whether this process occurs depends on the polarization and amplitude of the large-scale compressive mode. Compressive ion-acoustic modes (see Sect. 4.3.4) with reasonable magnetic fluctuation amplitudes ($\delta |\mathbf{B}|/B_0 \gtrsim 0.04$) can trigger this effect with temperature-anisotropy-driven instabilities under typical solar-wind conditions at 1 au. This *fluctuating-anisotropy effect* can be generalized to a *fluctuating-moment effect*, which includes, for instance, variations in relative drift speeds that may trigger additional instabilities.

## Conclusions

We briefly summarize our discussion of the multi-scale nature of the solar wind, give an outlook on future developments in the field, and outline the broader impact of this research topic.

### Summary

**Fig. 24:**
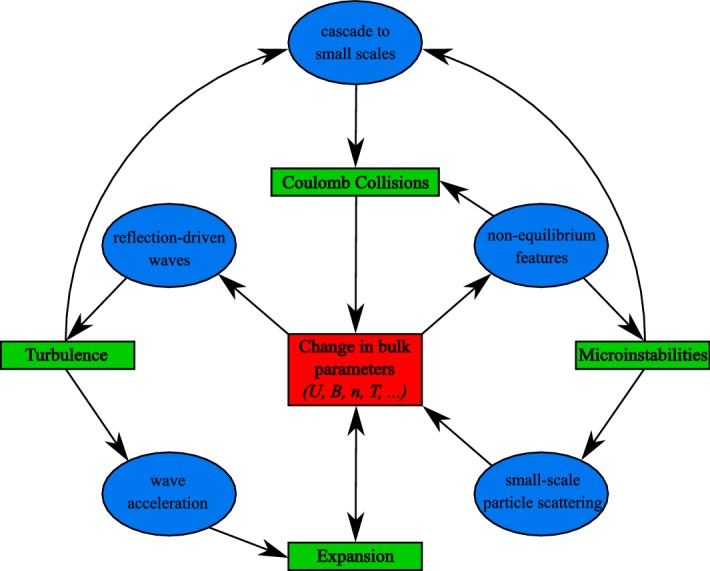
Summary of the multi-scale couplings in the solar wind. We describe the effects of collisions in Sect. 3, the effects of waves in Sect. 4, the effects of turbulence in Sect. 5, and the effects of microinstabilities in Sect. 6. The arrows illustrate the connections and interactions discussed in this review article

As we summarize in Fig. 24, the solar wind’s dynamics and thermodynamics result from an intricate multi-scale coupling between global expansion effects and local kinetic processes. The global expansion shapes particle distribution functions slowly compared to most of the collective plasma timescales and creates the ubiquitous non-equilibrium features of solar-wind particles. It also generates gradients in the plasma bulk parameters that drive Sunward-propagating waves, which subsequently interact with anti-Sunward-propagating waves to generate turbulence. By creating microphysical features and turbulence, the expansion couples to small scales and sets the stage for collisional relaxation, the dissipation of waves and turbulence, and kinetic microinstabilities to act locally. On the other hand, these local processes couple to the global scales and modify the large-scale plasma flow by, for example, accelerating the plasma, changing the plasma temperatures, introducing temperature anisotropies, regulating heat flux, or generating electromagnetic structures for particles to scatter on. These effects then modify the expansion. Figure 24 includes some processes (e.g., reflection-driven waves) that we will discuss in the next major update of this Living Review.

We derive our understanding of the solar wind’s multi-scale evolution from detailed measurements of its particles and fields. In-situ observations provide perspective on small-scale processes, while remote observations provide perspective on large-scale processes. Therefore, we rely on the combination of in-situ and remote observations, in concert with theoretical modeling efforts and numerical simulations to elucidate the multi-scale evolution of the solar wind. This review describes the current state of the art of the field based on a combination of observational discoveries and fundamental plasma physics.

### Future outlook

Major new space missions such as Parker Solar Probe (PSP; Fox et al. 2016) and Solar Orbiter (SO; Müller et al. 2013) are dedicated to the study of the processes at the heart of this review.

PSP, which launched in August 2018 and achieved its first perihelion in November 2018, is beginning to measure in-situ plasma properties with unprecedented energy and temporal resolution and at unexplored heliocentric distances (see Fig. 8). New findings derived from PSP will transform our understanding of plasma processes near the Sun. PSP is expected to provide our first in-situ observations of the corona, which are anticipated to draw together the heliospheric and solar communities and to enable novel combinations of in-situ and remote observations.

SO will measure the solar-wind properties through both in-situ measurements of the local plasma conditions and remote observations of the Sun’s surface. A major goal for SO is *linkage science*: connecting processes in and near the Sun with the behavior of solar-wind plasma across all relevant scales. SO’s inclined orbit will carry it out of the ecliptic plane and enable it to sample solar wind from polar coronal holes with its more extensive instrumentation package compared to PSP. Both PSP and SO will drive research into the multi-scale nature of the solar wind for decades.

Other heliospheric missions that are currently being developed and proposed will directly address the topics of this review. These include mission concepts to investigate the nature of waves and turbulence through *multi-point* and *multi-scale measurements* as well as mission concepts to resolve the smallest natural plasma scales in the solar wind (e.g., National Academy of Sciences, Engineering, and Medicine 2016; Klein et al. 2019; Matthaeus et al. 2019; TenBarge et al. 2019; Verscharen et al. 2019b). These efforts demonstrate that the heliophysics community understands the need to investigate the multi-scale couplings of plasma processes and their impact on the dynamics and thermodynamics of the solar wind.

We also anticipate major advances in modeling in the near future. Previously, numerical simulations of processes that connect over large scale separations required computational resources too great for them to be practical. Therefore, most models either focused on global expansion dynamics (e.g., global MHD simulations) or on local plasma processes (e.g., homogeneous-box particle-in-cell simulations).^22^ However, our increasing numerical capabilities will allow us to simulate self-consistently the coupling across scales of global and local processes in the near future. Even though a full particle-in-cell model of the heliosphere with realistic properties may still lie decades in the future, the ongoing improvement in our modeling capabilities will advance our understanding of the multi-scale nature of the solar wind.

### Broader impact

All magnetized plasmas exhibit a broad range of characteristic length scales and timescales. These span from the largest scales of the system to its microscopic scales: those of plasma oscillations, particle gyration, and electrostatic and electromagnetic shielding. The vast system sizes of space and astrophysical plasmas lead to especially large separations among these characteristic plasma scales. The solar wind exemplifies such a multi-scale astrophysical plasma, and the combination of solar-wind observations with fundamental plasma physics has improved our understanding of astrophysical plasma throughout the Universe. The solar wind’s expansion through the heliosphere introduces additional global scales that couple to the small-scale plasma processes. We anticipate that, in the coming years, the connection of small-scale kinetic processes with the large-scale thermodynamics of astrophysical plasmas will be a major research focus not only in heliophysics but throughout the astrophysics community.

The solar wind is the ideal place to study the multi-scale nature of astrophysical plasmas. The conditions of space and astrophysical plasmas cannot be reproduced and sampled with comparable accuracy in laboratories. With the notable exception of the very local interstellar medium, the only astrophysical plasmas that have been observed in situ are in the heliosphere.

Research into this topic serves a broader impact beyond the purely academic understanding of space and astrophysical plasmas. The study of the solar wind’s multi-scale nature enables a better understanding of its dynamics and thermodynamics based on first principles. This knowledge will be invaluable to the design of physics-based models for space weather and to guiding our efforts toward the successful prediction of space hazards for our increasingly technological and spacefaring society.

## Electronic supplementary material

Below is the link to the electronic supplementary material.

Movie of Fig. 5 (avi 9,996 KB) Interpolated isosurface in velocity space of the proton distribution function measured by Helios 2 on 1972-02-04 at 10:21:43 UTC. The z-axis indicates the direction of the background magnetic field. The elongation of the isosurface along the magnetic-field direction represents the proton beam.

Movie of Fig. 5 (avi 9,835 KB) Interpolated isosurface in velocity space of the proton distribution function measured by Helios 2 on 1976-04-16 at 07:50:54 UTC. The z-axis indicates the direction of the background magnetic field. The oblate structure of the distribution function represents a temperature anisotropy.

## Footnotes

1 Following the prevalent convention in space plasma physics, we adopt the metric system of Gaussian-cgs units. The NRL Plasma Formulary (Huba 2016) includes a guide to converting formulæ between cgs and SI units. In some figures, we plot magnetic field in nT for consistency with the published plots on which they are based.

2 We refrain from discussing the multiple ways of deriving the Boltzmann equation such as the closure of the BBGKY hierarchy (Bogoliubov 1946) or the Klimontovich–Dupree formalism (Dupree 1961; Klimontovich 1967). Instead, we express the Boltzmann equation in terms of Liouville’s theorem and subsume all higher-order particle interactions in the collision term on the right-hand side of Eq. (19). For more details, see also Sect. 3.2.

3 Interestingly, the inclusion of the pressure-gradient term from Eq. (55) in Eq. (56) does not affect the frozen-in condition since it cancels when taking the curl in Eq. (52).

4 The proton beam may be directed toward the Sun or be bi-directional if the local radial component of the magnetic field changed its sign during the passage of the plasma parcel from the Sun to the location of the measurement.

5 From *strahl*—the German word for “beam”.

6 Typically, the function $A(\theta ,\phi )$ is calculated from the Faraday cup’s geometry and/or is measured in ground testing. The value of $A(\theta ,\phi )$ is generally largest for $\theta = 0$, when particles flow straight into the cup, and then falls off as $\theta$ increases and less of the collector is “illuminated” by inflowing particles. If a Faraday cup has an asymmetric shape and/or multiple collectors, $A(\theta ,\phi )$ will also depend on $\phi$.

7 For example, *gold black* was used on the Wind/3DP ESAs (Lin et al. 1995).

8 For example, a carbon foil supported by a nickel mesh was used on Ulysses/SWICS (Gloeckler et al. 1992), ACE/SWICS (Gloeckler et al. 1998), and STEREO/IMPACT/PLASTIC (Galvin et al. 2008).

9 For example, the SWICS instruments on both Ulysses and ACE (Gloeckler et al. 1992, 1998) use a gold foil applied directly to the top of the detectors.

10 Unfortunately, no widely accepted term for $\mathbf{H}$ exists. Some authors (e.g., Jackson 1975) refer to it as the “magnetic field” and use another term for $\mathbf{B}$. Although there is some historical precedent for this naming convention, Sommerfeld (1952) and Griffiths (2013) strongly criticize it and contend that $\mathbf{B}$ is the more fundamental parameter. We follow the convention used widely in modern space physics of referring to $\mathbf{B}$ as the “magnetic field.” For $\mathbf{H}$, we choose the term “auxiliary field” from Griffiths (2013).

11 We note that *j* and *i* may refer to two different components of the same particle species (e.g., the proton core and proton beam, or the electron core and the electron halo).

12 We adopt the new terminology of Kasper et al. (2017). We note, however, that some earlier publications use the term “collisional age” for $N_{\mathrm {c}}$ (Kasper et al. 2008; Bale et al. 2009; Maruca et al. 2013).

13 These largely reversible fluctuations in entropy do not violate the second law of thermodynamics which only applies to the total entropy of a closed system.

14 In gyrokinetic theory, the collision frequency and $\omega _{\mathrm {r}}$ are both ordered to the intermediate timescale. This ordering does not prevent us from considering the collisionless and collisional limits and justifies the assumption of a Maxwellian $f_{0j}=f_{\mathrm {M}}$ (Howes et al. 2006; Schekochihin et al. 2008).

15 Although Eq. (161) was derived under the assumption of a Maxwellian background distribution, Kunz et al. (2018) derive an expression for $\mathrm {d}W/\mathrm {d}t$ assuming a drifting bi-Maxwellian $f_{0j}=f_{\mathrm {bM}}$.

16 The use of ${\bar{\epsilon }}$ in Eq. (166) and ${\bar{\delta }}$ in Eq. (167) reflects the importance of the two different stochastization mechanisms discussed in Sect. 4.2.3: the electrostatic potential in low-$\beta _{\mathrm {p}}$ plasmas and the magnetic field in high-$\beta _{\mathrm {p}}$ plasmas.

17 In fact, kinetic linear theory has an infinite number of solutions with this anti-correlation. However, almost all of them are so heavily damped with $|\gamma |\gg |\omega _{\mathrm {r}}|$ that they are irrelevant for all practical purposes to the solar wind.

18 The non-propagating kinetic slow mode is sometimes called the kinetic *entropy mode* in reference to the non-propagating MHD entropy mode. Although both modes share this non-propagating behavior, the MHD entropy mode is different from the kinetic slow mode in the sense that it does not exhibit variations in $\delta |\mathbf{B}|$.

19 We use the term “unpredictable” here to refer to the statistic nature of turbulence and the notion of randomness (Leslie 1973). The fluctuations in these quantities are still bound within certain limits and exhibit correlations.

20 The CGL marginal stability threshold arises at larger pressure anisotropies than those derived from kinetic theory (Klein and Howes 2015; Hunana and Zank 2017), which, combined with the limited relevance of a fluid theory to a weakly collisionless system, limits this instability’s relevance to the solar wind.

21 Plots of the data distribution in the $\beta _{\parallel \mathrm {p}}{-}T_{\perp \mathrm {p}}/T_{\parallel \mathrm {p}}$ plane have become colloquially known as “Brazil plots” due to the characteristic shape of the data distribution for near-Earth solar wind.

22 Notable exceptions to this dichotomy in global and local scales include expanding-box models and ad-hoc inclusions of kinetic processes through effective transport coefficients in global models.
